# Biodiversity Inventory and Distribution of Metriorrhynchina Net-Winged Beetles (Coleoptera: Lycidae), with the Identification of Generic Ranges

**DOI:** 10.3390/insects11100710

**Published:** 2020-10-16

**Authors:** Ladislav Bocak, Michal Motyka, Dominik Kusy, Renata Bilkova

**Affiliations:** Laboratory of Diversity and Molecular Evolution, Palacky University, 17. Listopadu 50, 771 46 Olomouc, Czech Republic; michal.motyka@upol.cz (M.M.); dominik.kusy@upol.cz (D.K.); renata.bilkova@upol.cz (R.B.)

**Keywords:** taxonomy, Australian region, distribution, diversity centers, endemism, new names, new combinations

## Abstract

**Simple Summary:**

We reviewed the classification of ~900 Metriorrhynchina net-winged beetles, and modified the alpha-taxonomy to reflect recently proposed phylogenetic hypotheses. There were >200 species transferred between subtribes and genera. The resulting checklist enabled us to identify New Guinea as the hotspot of Metriorrhynchina diversification. The centers of endemism include Sulawesi and New Guinea (in total 14 genera) in contrast with a single endemic genus in continental Australia. Most genera are diverse in New Guinea and the Wallacea, and only certain species crossed zoogeographic lines and colonized South East Asia. The study should boost the biodiversity research in the group and support biogeographic and evolutionary studies.

**Abstract:**

We reviewed the species-level classification of Metriorrhynchina net-winged beetles to make the group accessible for further studies. Altogether, 876 valid species are listed in a checklist along with known synonyms, combinations, and distribution data. The compilation of geographic distribution showed that Metriorrhynchina is distributed mainly in the Australian region with very high diversity in the islands at the northern edge of the Australian craton, i.e., in the Moluccas and New Guinea (54 and 423 spp. respectively). The neighboring northern part of the Australian continent houses a majority of known Australian species (112 spp.) and the diversity of net-winged beetles gradually decreases to the south (43 spp.). The fauna of Sulawesi is highly endemic at the generic level (4 of 10 genera, 67 of 84 spp.). Less Metriorrhynchina occur in the Solomon Islands and Oceania (in total 22 spp.). The Oriental Metriorrhynchina fauna consists of a few genera and a limited number of species, and most of these are known from the Philippines (51 of 94 Oriental spp.). We identified a high species level turn-over between all neighboring landmasses. The genus-level endemism is high in Sulawesi (4 genera) and New Guinea (11 genera), but only a single genus is endemic to Australia. During the compilation of the checklist, we identified some homonyms, and we propose the following replacement names and a new synonym: *Metriorrhynchus pseudobasalis*, nom. nov. for *M. basalis* Lea, 1921 nec *M. basalis* Bourgeois, 1911; *Metriorrhynchus pseudofunestus*, nom. nov. for *M. funestus* Lea, 1921 nec *M. funestus* (Guérin-Méneville, 1838), *Trichalus pseudoternatensis*, nom. nov. for *T. ternatensis* Kleine, 1930 nec *T. ternatensis* Bourgeois, 1900, *Procautires subparallelus*, nom. nov. for *P. parallelus* (Pic, 1926) nec *P. parallelus* (Bourgeois, 1883), and *Cautires pseudocorporaali*, nom. nov. for *C. corporaali* (Pic, 1921: 12), (formerly *Odontocerus* and *Cladophorus*) nec *C. corporaali* (Pic, 1921) (formerly *Bulenides*, later *Cautires*). *Diatrichalus biroi* Kleine, 1943, syn. nov. is proposed as a junior subjective synonym of *D. subarcuatithorax* (Pic, 1926). Altogether, 161 new combinations are proposed, and 47 species earlier placed in *Xylobanus* Waterhouse, 1879 transferred from Cautirina to Metriorrhynchina *incertae sedis*. The study clarifies the taxonomy of Metriorrhynchini and should serve as a restarting point for further taxonomic, evolutionary, and biogeographic studies.

## 1. Introduction

Net-winged beetles (Elateroidea: Lycidae) are among the major elateroid lineages, and their diversity is surpassed only by click and soldier beetles (10,000 and 5000 versus 4300 species; [1,2]). The Metriorrhynchini is the most diverse lycid tribe with over 1500 species known from the Old World [2]. Further species are continually described, sometimes with earlier described taxa representing only a minority if the local fauna is thoroughly revised [3,4,5,6,7,8,9,10]; Zoological Records Database. The rank and placement of the tribe Metriorrhynchini had been unstable [11,12,13,14,15] until, based on the phylogenomic analysis, it was merged with Oriental Libnetini, Dihammatini, Dilophotini, and Lycoprogenthini into the redefined Metriorrhynchinae [16].

**Figure 1:**
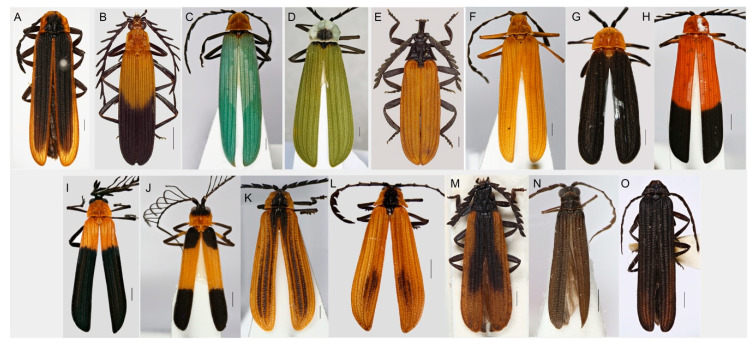
The representatives of the subtribe Metriorrhynchina, general appearance. (**A**) *Metriorrhynchus eremita* (Blackburn), Australia; (**B**) *M. doleschali* (Redtenbacher), Ceram; (**C**) *Metriorrhynchus* sp., New Guinea; (**D**) *Porrostoma* sp., New Guinea; (**E**) *Porrostoma rhipidium* (Macleay), Australia; (**F**) *Eniclases* sp., New Guinea; (**G**) *Synchonnus testaceithorax* Pic, New Guinea; (**H**) *Ditua* sp., New Guinea; (**I**) *Flabellotrichalus* sp. New Guinea; (**J**) *Pseudodontocerus* sp., New Guinea; (**K**) *Trichalus* sp., Australia; (**L**) *Synchonnus crypticus* Kusy et al., Australia; (**M**) *Porrostoma* sp., Australia; (**N**) *Wakarumbia linearis* Dvorak & Bocak, Sulawesi; (**O**) *Sulabanus* sp., Sulawesi. Scales 0.5 mm.

The Metriorrhynchini are net-winged beetles with the characteristic arrangement of pronotal carinae that form seven areoles (Figure 1A–O). All species have four or nine longitudinal and transverse elytral costae, although some of these can be substantially shortened and are present only in the humeral part of the elytra. The male genitalia do not have parameres, most species have the characteristic rounded phallobase, and female genitalia have a unique unpaired gland in the apical part of the vaginal sac [11,14,17]. The identification of Metriorrhynchini is typically intuitive; however, some species have reduced pronotal carinae and then the male genitalia provide the most reliable diagnostic character. 

The subtribe Metriorrhynchina was redefined based mostly on the molecular phylogeny and the only reliable characteristics for the subtribe were found in larvae, which have longitudinally divided meso- and metathoracic terga, and many have paired spines at the frontal margin of the pronotum [18,19,20]. The body size varies between 3 and 24 mm and the large-bodied species in particular are brightly colored and form extensive mimicry complexes [21,22,23]. 

All lycid larvae use unique larval mouthparts to suck liquids from decaying organic material [18,24]. The morphology of the larval mouthpart is so unique that it had been proposed that Coleoptera is polyphyletic and forms one of the deepest lineages of Neoptera [25], but such a hypothesis was quickly rejected [26]. The modified mouthparts limit the occurrence of net-winged beetles to at least seasonally humid habitats. The highest abundance was reported from tropical mountain ecosystems with constant high humidity, such as the mountain ranges of the Malay Peninsula, the Great Sundas, and New Guinea. The diversity is much lower in the temperate zone and net-winged beetles are absent in all arid areas without woody plants [2].

The studies on Metriorrhynchina cope with high diversity, poorly based placements of species due to nomenclatural problems and unclear concepts of genera [27], uninformative descriptions [28,29], and disrespect to the species described by other authors when new species are proposed. All these factors stand behind the chaotic taxonomy of the group. The situation is complicated also by the pitiful condition of some collections holding primary types. To clarify the classification of the groups and make the Metriorrhynchini accessible for the evolutionary studies, the tribe was recently studied using the DNA data, and, after comparing the molecular hypotheses with the morphology, three subtribes were proposed: Metanoeina Sklenarova et al., 2014, Cautirina Sklenarova et al., 2014, and Metriorrhynchina Kleine, 1926, several genus concepts were revised [20], and the generic limits were investigated [30,31,32].

In continuing the studies on Metriorrhynchina, first, we must review the results of previous studies. Due to the enormous diversity of net-winged beetles, the fauna of the Wallacea, New Guinea, and Australia has not been cataloged since the publication of Kleine’s work published as a part of the series “Coleopterorum Catalogus”, edited by W. Junk & S. Schenkling [3], except the chapter Lycidae in the Zoological Catalogue of Australia [27]. Since the compilation of both catalogs, the classification of the Metriorrhynchine net-winged beetles has substantially changed. The tribes Cladophorini, Dilolycini, and Trichalini were merged with Metriorrhynchini [11], and along with the later erected subtribe Hemiconderina Bocak & Bocakova, 1990, were synonymized with Metriorrhynchina [20]. 

The Conderini, for some time considered as a sister-group of Metriorrhynchini, is now placed in Lycinae [16]. The nomenclatural uncertainty of *Metriorhynchus* Guérin-Méneville, 1938 affected the classification presented in the Zoological Catalogue of Australia [27], and most Australian species were placed in *Porrostoma* Laporte, 1838. The identity and nomenclature of the genus-group names in the *Porrostoma*-*Metriorrhynchus* complex were later studied in detail, the original concept of *Metriorhynchus* was resurrected, and *Metriorrhynchus* Gemminger et Harold, 1869 was used as an available name to replace *Metriorhynchus* Guérin-Méneville, 1838. The type species of *Metriorrhynchus* and *Porrostoma* represent distant lineages and both genera were considered valid although the limits of these genera still require further investigation [7,33]. In the course of taxonomic studies, several genera were synonymized, e.g., *Hemiconderis* Kleine, 1926; *Mimotrichalus* Pic, 1930, *Samanga* Pic, 1921 [34,35,36], and numerous species were described [4,5,6,7,8,9,37,38,39,40,41,42,43,44,45].

The alpha-taxonomic research of the Metriorrhynchini has a checkered history in all regions. The Australian region is the center of Metriorrhynchina diversity [3,12,18]. The first Australian species were reported from the coastal areas in the early 19th century [46,47]. Later, the collection of the British Museum was studied, and several Australian genera and species were described by C. O. Waterhouse [48,49,50]. All these studies were fragmentary due to the limited numbers of available samples. The majority of Australian fauna was described by T. Blackburn [51,52,53], W. M. Macleay [54,55], and A. M. Lea [56,57,58,59,60,61,62,63,64]. Since then, only a few new species have been described [10,29,65]. 

Similarly, the first New Guinean Metriorrhynchina species were reported already from collections brought to Europe by the first explorers of the Pacific region [66,67], and the majority of currently known species were described in the period of a single decade [68,69,70]. Further alpha-taxonomic research of the New Guinea fauna was conducted by L. Bocak and M. Bocakova [39,45], S. V. Kazantsev [6,7,8,9], and several other authors [71,72,73,74,75,76]. 

The first species of Metriorrhynchina from the Wallacea was described already by Fabricius [46], and other species were added from samples collected by A. R. Wallace; however, information on the local fauna remained anecdotic for a long time. The fauna of Wallacea was mostly studied by R. Kleine and M. Pic before WWII [28,69,77] and later by further entomologists dealing with net-winged beetles [4,5,30,35]. 

The Metriorrhynchina fauna is insufficiently studied, and several genera are used as typological collective taxa for a provisional placement of newly described species, e.g., some Metriorrhynchina species have been even recently described in *Xylobanus* Waterhouse, 1879 even though *Xylobanus* belongs to the subtribe Cautirina, which does not occur in the Australian region [6]. 

The fauna of the transitional zone between the Asian and Australian cratons is extremely diverse [78,79,80] and has served as a model for zoogeographical studies since the pioneering work of A.R. Wallace [81,82,83,84]. The net-winged beetles with their limited dispersal propensity are a promising model for further zoogeographical studies [85,86,87,88,89,90], but serious investigations should be based on the critical inventory of the alpha diversity and phylogenetically based classification.

Therefore, we present the checklist of Metriorrhynchina that represents the majority of the species placed in the tribe and a substantial portion of Metriorrhynchini genera. The work reflects the current state of the knowledge as many original generic placements have been changed, the traditional generic concept did not hold, and additional species have been described [16]. As a result, the checklist is not a simple update of those published earlier [3,27]. The present review of the Metriorrhynchina diversity should represent a re-starting point for further research on this group Along with the checklist, we propose new replacement names, revise the placements of over two hundred of species, and we exclude from Metriorrhynchina certain species that do not belong to this group. The study additionally summarizes the current knowledge on the distribution of Metriorrhynchina genera, the defined centers of diversity, and the structure of regional faunas.

## 2. Methods

The primary descriptions, catalogs [3,27,91], and the Zoological Records database (Thompson Scientific) were searched for original species and genus descriptions of the Metriorrhynchina and all taxonomical acts affecting their validity, generic placement, and species concepts. Further, earlier published studies were considered for the identification of the Metriorrhynchina and Cautirina distribution ranges [2,89]. 

**Figure 2:**
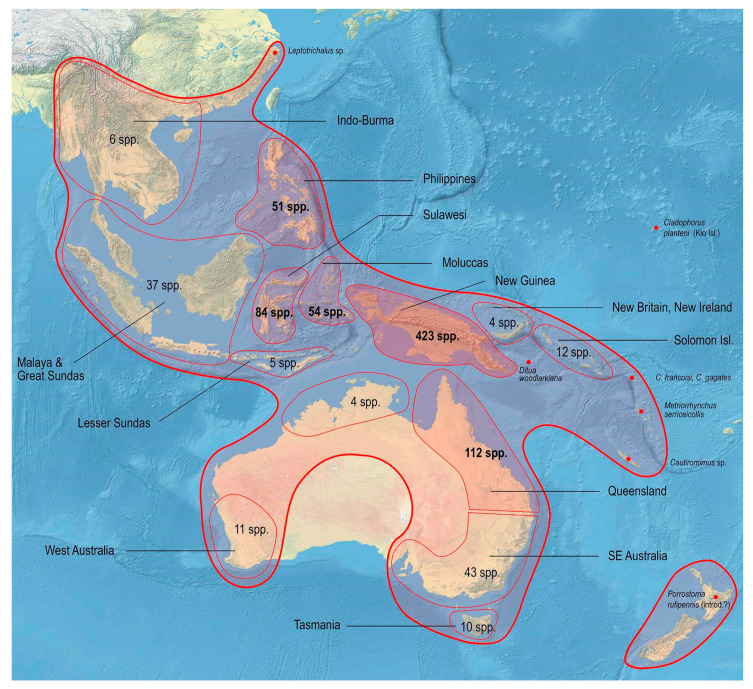
Distribution and diversity of the subtribe Metriorrhynchina.

**Figure 3:**
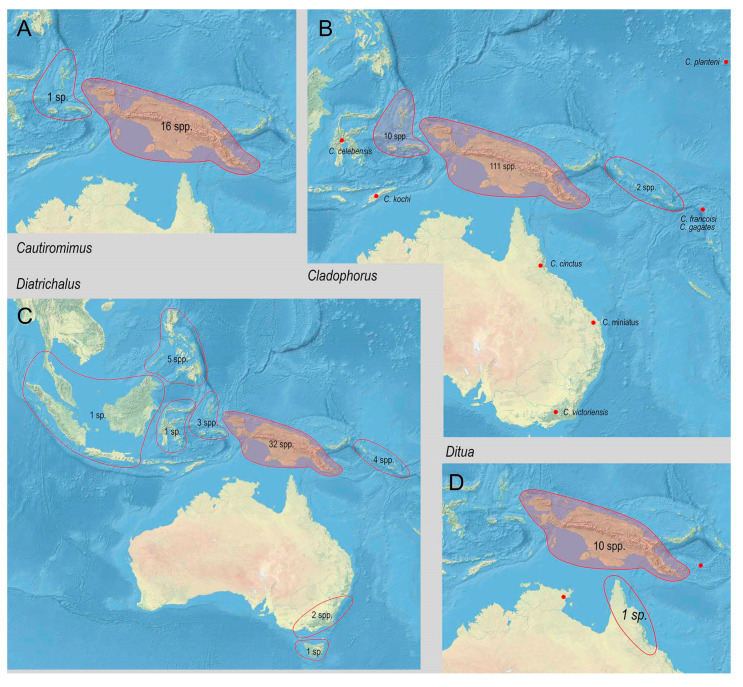
Distribution and diversity of Metriorrhynchina genera. (**A**) Cautiromimus Pic, 1926; (**B**) Cladophorus Guérin-Méneville, 1830; (**C**) Diatrichalus Kleine, 1926; (**D**) Ditua Waterhouse, 1979.

**Figure 4:**
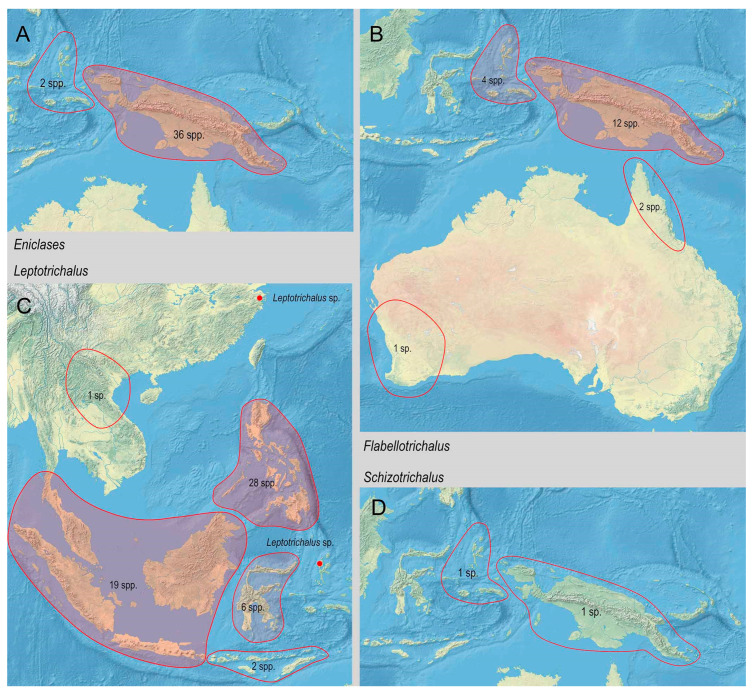
Distribution and diversity of Metriorrhynchina genera. (**A**) *Eniclases* Waterhouse, 1979; (**B**) *Flabellotrichalus* Pic, 1921; (**C**) *Leptotrichalus* Kleine, 1925; (**D**) *Schizotrichalus* Waterhouse, 1879.

**Figure 5:**
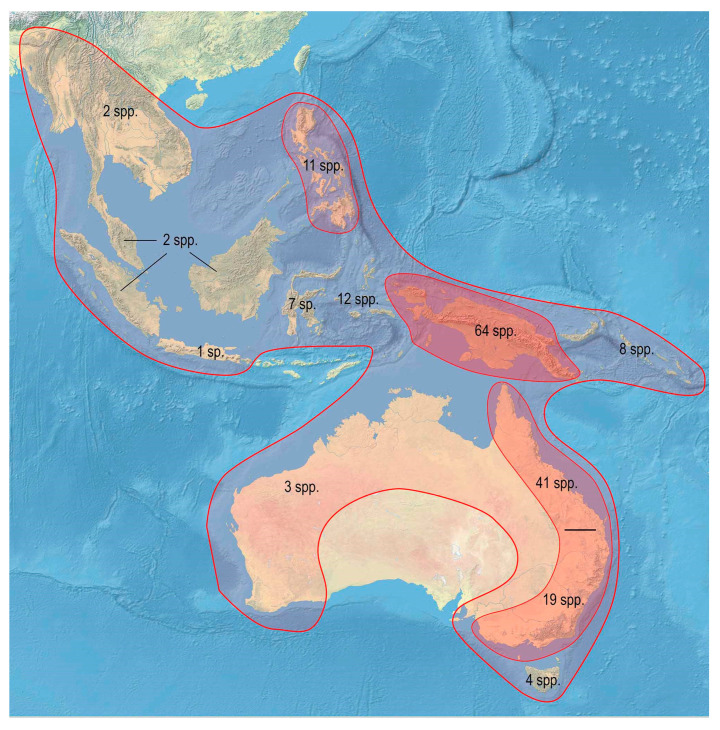
Distribution and diversity of *Metriorrhynchus* Gemminger & Harold, 1869.

**Figure 6:**
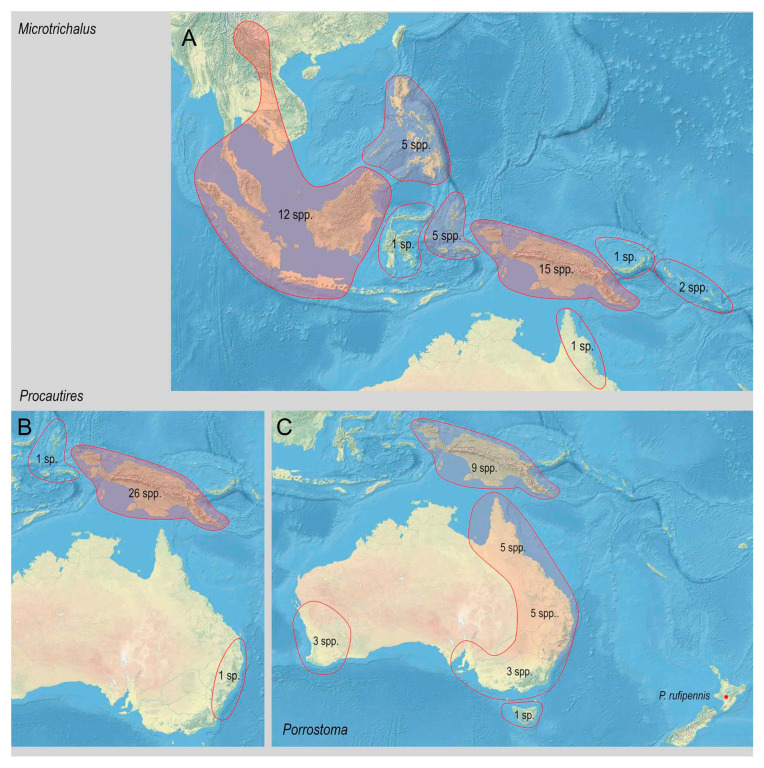
Distribution and diversity of Metriorrhynchina genera. (**A**) *Microtrichalus* Pic, 1921; (**B**) *Procautires* Kleine, 1926; (**C**) *Porrostoma* Laporte, 1838.

**Figure 7:**
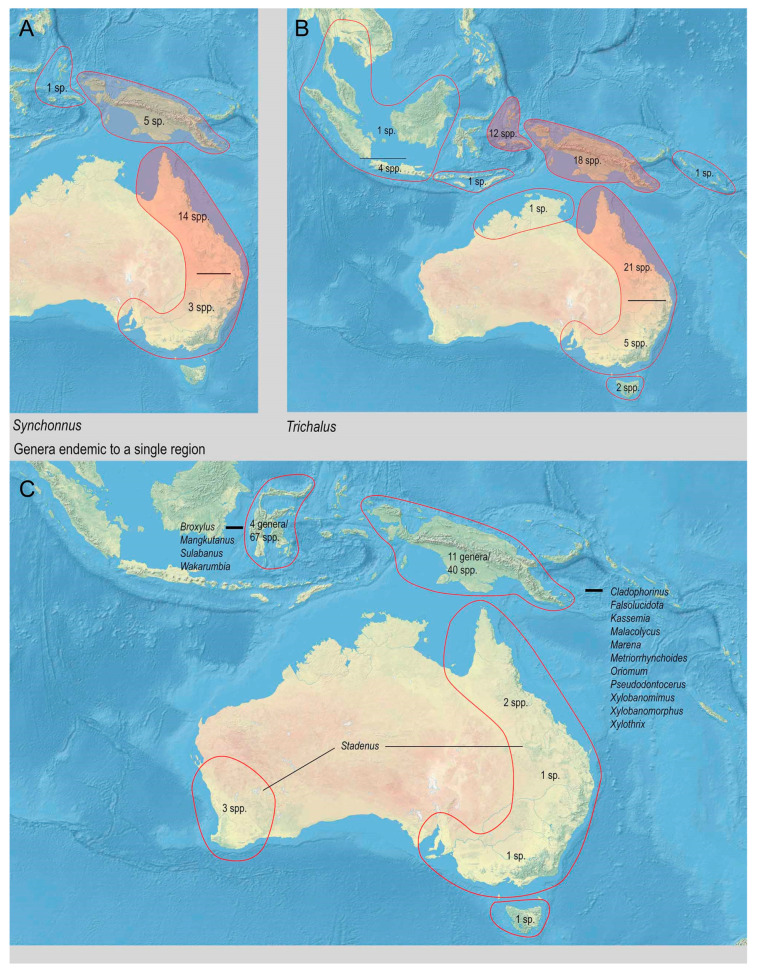
Distribution and diversity of Metriorrhynchina genera. (**A**) *Synchonnus* Waterhouse, 1879; (**B**) *Trichalus* Waterhouse, 1877; (**C**) The genera endemic to a single region.

The range of Metriorrhynchina was divided into following regions, which are characterized by almost complete species turnover: Australia: (1) West, (2) south-eastern (Victoria, New South Wales, and Australian Capital Territory), (3) Tasmania, (4) Queensland, and (5) the Northern Territory (only six Australian species occur in two or more regions, all species are assigned to here defined subregions whenever possible, but some records are limited to the locality data “Australia” without any further information); (6) New Guinea and the surrounding islands separated by shallow sea (Aru islands, Raja Ampat; and Japen, Biak; no species shared with Australia, a single species shared with New Britain, but its identity not confirmed); (7) New Britain and New Ireland; (8) the Solomon Islands and Oceania (Woodlark, Marshal Islands, Vanikoro, and Vanuatu; all species endemic to a single island); (9) the Moluccas (a single shared species with Sulawesi); (10) Sulawesi and surrounding islands (Buton, Muna, Banggai, etc.); (11) the Lesser Sundas (Lombok, Sumbawa, Sumba, Flores, Timor, and Wetar Isl.) and Tanimbar Isl.; (12) the Philippines including Palawan; (13) the Great Sundas and the Malay Peninsula up to the Isthmus of Kra; and (14) Indo-Burma (Figure 2, Figure 3, Figure 4, Figure 5, Figure 6 and Figure 7). 

The numbers of species and genera were recorded for these regions and are presented in the maps for each genus. The species composition similarity is not evaluated in detail as only two questionable records are available for species that putatively occur in two regions. As a result, most indexes quantifying the similarity of regional faunas would have zero value and no conclusions could be made. The generic composition of regional faunas is also evaluated without quantification with similarity indexes as our study is based on the compilation of primary taxonomic literature and distribution records and we do not have robust phylogenetic hypotheses to identify the centers of origins and dispersal routes for individual genera. Instead, we show the numbers of species in the respective regions, the proportion of endemic genera, and the number of species belonging to endemic genera. These data approximately indicate where the centers of diversification are located and should serve as guidance for further studies.

The main collections housing primary types were studied in the course of the last 30 years by the first and second authors. Further, we studied the assembled material of the voucher collection of the Laboratory of Biodiversity and Molecular Evolution of the Palacky University in Olomouc. Approximately 4000 specimens were available from the Oriental region, 1000 from Sulawesi, 1000 from the Moluccas, 18,000 from New Guinea, and 1000 from Australia. Additionally, we studied about 10,000 specimens collected by the expeditions of the Bernice Pauhu Bishop Museum in Honolulu, USA. These data were assembled during a long-time search in collections, which enabled us to identify the ranges of Metriorrhynchina genera and to compare them with literature data, particularly with recent molecular analyses that identified the centers of the origin for Metriorrhynchine subtribes [2,20,89].

The list of studied collections housing Metriorrhynchina collections: Museum and Institute of Zoology in Warsaw (coll. R. Kleine), Poland; National Museum of Natural History in Paris (coll. M. Pic, J. Bourgeois, and L. Fairmaire), France; Natural History Museum in London (the species described by C.O. Waterhouse), United Kingdom; Naturalis Museum, Leiden (including the recently transferred collection of the Amsterdam University), the Netherlands; Museum of Natural History in Brussels (coll. Guérin-Méneville), Belgium; Museum Alexander Koenig in Bonn, Germany; Bavarian State Collection of Zoology, Munich, Germany; Senckenberg Natural History Collections, Dresden (coll. Kirsch), Germany; Natural History Museum in Vienna, Austria; Natural History Museum in Basel, Switzerland; Natural History Museum in Geneva, Switzerland; Queensland Museum in Brisbane, Queensland, Australia; Natural History Museum in Berlin, Germany; Stuttgart State Museum of Natural History, Germany; National Museum in Prague, Czech Republic; Hungarian Natural History Museum, Budapest (coll. Biró), Hungary; Entomological Collection of the University of the Philippines, Los Baños (coll. C.F. Baker), the Philippines; Indonesian Institute of Sciences (LIPI) in Cibinong; and Zoological Museum of the National University of Singapore, Singapore.

## 3. Results and Discussion

Altogether, 876 valid species are listed in a checklist along with known synonyms, combinations, and distribution data (see Supplementary Materials). The compilation of geographic distribution shows that Metriorrhynchina is distributed mainly in the Australian region. Only a limited number of genera and species reached the eastern part of Asia and the southernmost part of the Palearctic region (Figure 2).

### 3.1. The Justification of Nomenclatural Acts Proposed in this Study

Metriorrhynchina has been recently redefined [20], and they are a sister group of Cautirina. These species can be placed to respective subtribes often only based on the morphology of genitalia as the minute differences in the shape of the pronotal carinae and elytral costae are difficult to define [10,17]. Further, these groups substantially differ in their distribution [89]. Cautirina occurs in the Oriental, Afrotropical, and Palearctic regions in about 650 spp. [3; Zoological Record database], and only a few species have been recorded in Sulawesi but not further to the east [17,87]. The genera *Cautires* Waterhouse,1879, and *Xylobanus* Waterhouse, 1879 (both Cautirina) have been used as basket-taxa by earlier authors [69,92] and this practice has continued until recently when New Guinea species were placed in the cautirine genus *Xylobanus* [6]. 

Here, all Papuan species described in *Cautires* are transferred to *Cautiromimus* Kleine, 1926, a genus with high diversity in New Guinea and highly variable morphology that can be easily misidentified as *Cautires*: *Cautiromimus amabilis* (Waterhouse, 1884), comb. nov., *C. atroscutus* (Pic, 1922), comb. nov., *C. elegans* (Kleine, 1926), comb. nov., *C. facetus* (Kleine, 1926), comb. nov., *C. factus* (Kleine, 1926), comb. nov., *C. fuliginosus* (Kleine, 1926), comb. nov., *C. insulanus* (Kleine, 1926), comb. nov., *C. kristinae* (Kazantsev, 2010), comb. nov., *C. maculosus* (Kleine, 1926), comb. nov., *C. maturus* (Kleine, 1926), comb. nov., *C. mediocris* (Kleine, 1926), comb. nov., *C. mendicus* (Kleine, 1926), comb. nov., *C. mendosus* (Kleine, 1926), comb. nov., *C. mimicus* (Kleine, 1926), comb. nov. (all fourteen originally *Cautires* Waterhouse, 1879). 

The phylogenetically correct placement of the species described in *Xylobanus* is more complicated. The genus is characterized by four primary costae in each elytron; however, there are an additional six Papuan and Australian genera of Metriorrhynchina with absent secondary costae at least in a part of their elytra (*Procautires* Kleine, 1926, *Xylobanomimus* Kleine, 1926, *Malacolycus* Kleine, 1943, *Mimoxylobanus* Pic, 1921, *Xylobanomorphus* Kleine, 1935, and *Xylothrix* Kazantsev, 2015) and their limits currently remain unclear. Additional genera with a similar morphology known from Sulawesi and New Guinea are only distantly related (e.g., *Diatrichalus* Kleine, 1926, *Sulabanus* Dvorak & Bocak, 2007, *Kassemia* Bocak, 1998 [20]. As we are not able to correctly place the species earlier described in *Xylobanus* (Cautirina), we transfer these species to Metriorrhynchina, but we leave them incertae sedis pending future type-based revisions. 

This act affects the placement of 47 species from New Guinea and Australia: *ampliatus* Macleay, 1887, *australianus* Kleine, 1927, *baitetaensis* Kazantsev, 2015, *basiflavus* Lea, 1909, *cancellatus* Lea, 1909, *canus* Kleine, 1927, *confluens* Bourgeois, 1900, *conquisitus* Kleine, 1927, *coenosus* Lea, 1899, *constricticollis* Lea, 1909, *corvus* Kleine, 1935, *culex* Kazantsev, 2015, *densereticulatus* Kleine, 1927, *diminutivus* Lea, 1909, *fakfakensis* Kazantsev, 2015, *flavomarginatus* Kleine, 1933, *fumosus* Macleay, 1887, *hackeri* Kleine, 1933, *heterodoxus* Lea, 1909, *insignipennis* Blackburne, 1900, *longicornis* Macleay, 1887, *meyricki* Blackburne, 1886, *milnei* Pic, 1923, *miniaticollis* Macleay, 1887, *mirabilis* Kleine, 1926, *mobilis* Kleine, 1926, *modestus* Kleine, 1926, *neglectus* Kleine, 1926, *nigronotatus* Pic, 1923, *obscurus* Macleay, 1886, *parvulus* Pic, 1922, *pecten* Kazantsev, 2015, *piceoscutus* Pic, 1922, *ramosus* Lea, 1909, *robustithorax* Kleine, 1927, *rotundeareolatus* Kazantsev, 2015: 109, *simplicicornis* Lea, 1909, *sodalis* Kleine, 1935, *telefominensis* Kazantsev, 2015, *testaceicollis* Macleay, 1887, *testaceoapicalis* Pic, 1923, *testaceohumeralis* Pic, 1923, *testaceoscutus* Pic, 1922, *uniseriatus* Lea, 1909, *venustus* Kleine, 1935, *versicolor* Kleine, 1927, and *waigeoensis* Kazantsev, 2015. Three species are excluded from *Metriorrhynchus* and provisionally placed incertae sedis with other species with four primary costae: *mimicus* (Lea, 1921), *pseudobasalis* (Bocak et al. 2020), and *tamborinensis* (Calder, 1998). 

*Falsolucidota* Pic, 1921 was a single genus not available for the morphology-based generic revision of Metriorrhynchini [17], and the type species *Falsolucidota testaceicollis* Pic, 1921 was located in the disorganized Pic’s collection [36]. *Falsolucidota* was found congeneric with *Hemiconderis* Kleine, 1926, but only *F. testaceicollis* Pic, 1921 and *H. explicatus* Kleine, 1926 were mentioned. Therefore, eight species described in the revision of *Hemiconderis* [45] need to be formally transferred to *Falsolucidota* Pic, 1923: *Falsolucidota anthracina* (Bocak, 1999), comb. nov., *F. bipustulata* (Bocak & Bocakova, 1990), comb. nov., *F. bistriata* (Bocak & Bocakova, 1990), comb. nov., *F. niger* (Bocak & Bocakova, 1990), comb. nov., *F. pallidihumeralis* (Bocak & Bocakova, 1990), comb. nov., *F. samuelsoni* (Bocak & Bocakova, 1990), comb. nov., *F. sedlaceki* (Bocak & Bocakova, 1990), comb. nov., and *F. suturalis* (Bocak & Bocakova, 1990), comb. nov. (all originally in *Hemiconderis* Kleine, 1926).

Calder [27] considered *Metriorhynchus* Guérin-Méneville, 1838 as a junior homonym of *Metriorhynchus* Meyer, 1830 (Crocodylia) and transferred all species placed in this genus to *Porrostoma* Laporte, 1838. Bocak [33] resurrected the *Metriorhynchus* concept using an unjustified emendation by Gemminger and Harold [91] as a valid replacement name and provided diagnostic characteristics for the delimitation of *Metriorrhynchus* and *Porrostoma*. Here, the species transferred to *Porrostoma* [27] are transferred to *Metriorrhynchus.* As the types of these species were not studied, we follow the original placements given in descriptions or later placements that preceded the Calder’s nomenclatural acts. 

Altogether, 79 species are returned to *Metriorrhynchus*: *M. abdominalis* (Waterhouse, 1877), comb. nov., *M. angustus* Lea, 1922, comb. nov., *M. apicivarius* Lea, 1921, comb. nov., *M. apterus* Lea, 1909, comb. nov., *M. atratus* (Fabricius, 1801), comb. nov., *M. batesi* Lea, 1909, comb. nov., *M. breveapicalis* Pic, 1923, comb. nov., *M. brisbanensis* Lea, 1909, comb. nov., *M. centralis* Macleay, 1887, comb. nov., *M. cliens* Blackburn, 1900, comb. nov., *M. compositus* Lea, 1921, comb. nov., *M. connexus* Lea, 1929, comb. nov., *M. costicollis* Lea, 1921, comb. nov., *M. crassipes* Lea, 1921, comb. nov., *M. cryptoleucus* Lea, 1921, comb. nov., *M. decipiens* Lea, 1929, comb. nov., *M. dentipes* Lea, 1921, comb. nov., *M. diffusimaculatus* Kleine, 1928, comb. nov., *M. disconiger* Lea, 1909, comb. nov., *M. elongatus* Macleay, 1887, comb. nov., *M. eremitus* Blackburn, 1900, comb. nov., *M. eucerus* Lea, 1921, comb. nov., *M. femoralis* Macleay, 1872, comb. nov., *M. filirostris* Lea, 1929, comb. nov., *M. flagellatus* Blackburn, 1900, comb. nov., *M. flavipennis* Lea, 1921, comb. nov., *M. flavolimbatus* Lea, 1921, comb. nov., *M. foliatus* Macleay, 1887, comb. nov., *M. franklinmuelleri* Kleine, 1928, comb. nov., *M. frater* Lea, 1921, comb. nov., *M. fuligineus* Lea, 1921, comb. nov., *M. gracilis* Lea, 1909, comb. nov., *M. hackeri* Kleine, 1928, comb. nov., *M. hexastichus* Lea, 1921, comb. nov., *M. hirtipes* Macleay, 1887, comb. nov., *M. insignicornis* Lea, 1921, comb. nov., *M. insignipes* Lea, 1929: 337, comb. nov., *M. kingensis* Lea, 1908, comb. nov., *M. lateratius* Lea, 1921, comb. nov., *M. longepilosus* Kleine, 1928, comb. nov., *M. longicollis* Lea, 1929, comb. nov., *M. macphersonensis* Calder, 1998, comb. nov., *M. marginipennis* Lea, 1899, comb. nov., *M. mentior* Blackburn, 1900, comb. nov., *M. militaris* Lea, 1909, comb. nov., *M. minor* Lea, 1921, comb. nov., *M. minutus* Lea, 1921, comb. nov., *M. mirabilis* (Pic, 1923), comb. nov., *M. moerens* Lea, 1909, comb. nov., *M. modicus* Lea, 1921, comb. nov., *M. mollicollis* Lea, 1929, comb. nov., *M. monticola* Blackburn, 1892, comb. nov., *M. nigricauda* Kleine, 1928, comb. nov., *M. nigripes* Macleay, 1872, comb. nov., *M. occidentalis* Blackburn, 1892, comb. nov., *M. opacus* Lea, 1909, comb. nov., *M. ordinarius* Lea, 1909, comb. nov., *M. pallidominor* Lea, 1921, comb. nov., *M. parvoniger* Lea, 1921, comb. nov., *M. paradoxa* Blackburn, 1900, comb. nov., *M. pectinicornis* Lea, 1929, comb. nov., *M. pertenuis* Lea, 1929, comb. nov., *M. posticalis* Macleay, 1887, comb. nov., *M. pusillus* Kleine, 1928, comb. nov., *M. queenslandicus* Kleine, 1928, comb. nov., *M. quinquecavus* Lea, 1921, comb. nov., *M. ramicornis* Lea, 1922, comb. nov., *M. ruficollis* Lea, 1921, comb. nov., *M. rufirostris* Lea, 1909, comb. nov., *M. rufomarginatus* Lea, 1921, comb. nov., *M. sculpticollis* Lea, 1921, comb. nov., *M. semiflavus* Lea, 1929, comb. nov., *M. semiochraceus* Pic, 1923, comb. nov., *M. sinuaticollis* Pic, 1923, comb. nov., *M. tenebricosus* Kleine, 1928, comb. nov., *M. tibialis* Lea, 1909, comb. nov., *M. tricavicollis* Lea, 1921, comb. nov., *M. trichocerus* Lea, 1921, comb. nov., and *M. uniformis* (Waterhouse, 1877), comb. nov. (see the Appendix for details on earlier transfers of these species between genera of Metriorrhynchina). 

We had an opportunity to study the collection of the National Natural History Museum in the Paris museum, and we found that *Eros woodlarkianus* Montrouzier, 1857 does not belong to Erotinae [3]. Here, we transfer this species to Metriorrhynchinae: Metriorrhynchina and propose the new combination *Ditua wooklarkiana* (Montrouzier, 1857), comb. nov. Several further species were overlooked when generic concepts were revised, and they need to be placed to the genera with their closest relatives. Therefore, *Pseudodontocerus ruficollis* (Guérin-Méneville, 1938), comb. nov. is for the species earlier placed in *Carathrix* Kleine, 1926, which is a junior synonym of *Pseudodontocerus* [17], *Flabellotrichalus crinitus* (Kleine, 1926), comb. nov. is the species transferred from *Villosotrichalus* Pic, 1921, which is a junior synonym of *Flabellotrichalus* Pic, 1921 [17]. 

*Leptotrichalus chapaensis* Pic, 1923, comb. nov. is transferred from *Trichalus* Kleine, 1925, based on the study of the type of this species from Northern Vietnam, which is deposited in Pic’s collection in Paris. The study of the type material of several Australian species resulted in the following nomenclatural acts: *Ditua pectinicornis* Lea, 1929, comb. nov. is proposed for *Porrostoma pectinicornis* (Lea, 1929), and *Porrostoma forticostatum* (Pic, 1925a), comb. nov. is a new combination proposed for Australian *Cladophorus forticostatus* Pic, 1925.

*M. pseudobasalis* Bocak et al., 2020, *P. mimicus* (Lea, 1921), and *P. tamborinensis* Calder, 1998 are transferred from *Metriorrhynchus* and *Porrostoma* to Metriorrhynchina *incertae sedis* (a group of species with four primary costae, most of them earlier placed in *Xylobanus*). *Diatrichalus subarcuatithorax* (Pic, 1926), comb. nov. is transferred from *Trichalus* Waterhouse, 1879, and *D. biroi* Kleine, 1943a: 152, syn. nov. is proposed as its junior subjective synonym based on the study of both holotypes [39]. *Trichalus gorhami* Pic, 1926, comb. nov. is transferred to *Microtrichalus* Pic, 1921 also based on the morphology of the holotype.

The uncertainty in taxonomic concepts of several metriorrhynchine genera resulted also in repeated descriptions of species that belong to Cautirina in the genera now placed in Metriorrhynchina. Already earlier, the concept of *Cladophorus* was narrowed exclusively to the species from the Australian region [17], and the African species described in *Cladophorus* and *Procautires* were cataloged in *Cautires* by Kazantsev [93]. Unlike these, numerous Asian species of *Metriorrhynchus, Cladophorus*, and *Procautires* have not been mentioned in recent studies. Therefore, they formally remain placed in Metriorrhynchina [3] and need to be formally transferred to Cautirina. 

Therefore, we transfer the following fifty Asian species from Metriorrhynchina to Cautirina: *Cautires angusteareolatus* (Pic, 1942), comb. nov., *C. atricolor* (Pic, 1923), comb. nov., *C. atrithorax* (Pic, 1925), comb. nov., *C. atronotatus* (Pic, 1923), comb. nov., *C. atropunctatus* (Pic, 1921), comb. nov., *C. basimaculatus* (Kleine, 1939), comb. nov., *C. bidentatus* (Pic, 1925), comb. nov., *C. binaluanus* (Pic, 1925), comb. nov., *C. binhanus* (Pic, 1926), comb. nov., *C. brevilimbatus* (Pic, 1923), comb. nov., *C. carbonarius* (Bourgeois, 1898), comb. nov., *C. castetsi* (Pic, 1925), comb. nov. *C. congruens* (Pic, 1929), comb. nov., *C. cyaneiceps* (Pic, 1923), comb. nov., *C. fortesculptus* (Pic, 1939), comb. nov., *C. fragilis* (Kleine, 1926), comb. nov., *C. hoanus* (Pic, 1926), comb. nov., *C. inapicalis* (Pic, 1926), comb. nov., *C. incompletus* (Pic, 1939), comb. nov., *C. jeanvoinei* (Pic, 1939), comb. nov., *C. karnyi* (Kleine, 1931), comb. nov., *C. laboisieri* (Pic, 1925), comb. nov., *C. laosensis* (Pic, 1926), comb. nov., *C. latefenestratus* (Pic, 1939), comb. nov., *C. laticollis* (Pic, 1926), comb. nov., *C. monticola* (Kleine, 1928), comb. nov., *C. nigroapicalis* (Kleine, 1931), comb. nov., *C. nigroareolatus* (Pic, 1942), comb. nov., *C. nigropallidus* (Kleine, 1928), comb. nov., *C. notatithorax* (Pic, 1923), comb. nov., *C. obliteratus* (Pic, 1925), comb. nov., *C. orientalis* (Waterhouse, 1879), comb. nov., *C. particularis* (Pic, 1926), comb. nov., *C. particularithorax* (Pic, 1925), comb. nov., *C. planatus* (Kleine, 1926), comb. nov., *C. prominulithorax* (Pic, 1931), comb. nov., *C. rouyeri* (Pic, 1921), comb. nov., *C. rubicundus* (Waterhouse, 1879), comb. nov., *C. rudeplicatus* (Pic, 1925), comb. nov., *C. salvazai* (Pic, 1929), comb. nov., *C. satanas* (Bourgeois, 1906), comb. nov., *C. satrapa* (Bourgeois, 1905), comb. nov., *C. solutus* (Kleine, 1939), comb. nov., *C. testaceopunctatus* (Pic, 1921) (all from *Cladophorus* Guérin-Méneville, 1830); *C. amoenus* (Gorham, 1882), comb. nov., *C. antinorii* (Bourgeois, 1883), comb. nov., *C. dessumi* (Pic, 1949), comb. nov., *C. infuscatus* (Gorham, 1882), comb. nov., *C. rubicundus* (Waterhouse, 1879), comb. nov. (the last five species from *Metriorrhynchus* Gemminger & Harold, 1969); *C. fuscoreticulatus* (Kleine, 1933), comb. nov., and *C. socius* (Kleine, 1926), comb. nov. (both from *Procautires* Kleine, 1926). 

During the compilation of the checklist, we identified some primary and secondary homonyms, and we propose the following replacement names and a new synonym: *Metriorrhynchus pseudobasalis* Bocak et al., 2020, nom. nov. for *M. basalis* Lea, 1921 nec *M. basalis* Bourgeois, 1911; *Metriorrhynchus pseudofunestus* Bocak et al., 2020, nom. nov. for *M. funestus* Lea, 1921 nec *M. funestus* (Guérin-Méneville, 1838), *Trichalus pseudoternatensis* Bocak et al., 2020, nom. nov. for *T. ternatensis* Kleine, 1930 nec *T. ternatensis* Bourgeois, 1900, *Cautiromimus subparallelus* Bocak et al., 2020, nom. nov. for *P. parallelus* (Pic, 1926) nec *P. parallelus* (Bourgeois, 1883), and *Cautires pseudocorporaali* Bocak et al., 2020, nom. nov. for *C. corporaali* (Pic, 1921: 12) (formerly *Odontocerus* [28] and *Cladophorus* [3]), comb. nov. nec *C. corporaali* (Pic, 1921:8) (formerly *Bulenides* [12] and transferred to *Cautires* [31]).

Additionally, *Metriorrhynchus antinorii* Bourgeois (1983) described from Choa in Ethiopia has not been mentioned by Kazantsev (2012), and we propose to transfer this species to Cautirina as *Cautires antinorii* (Bourgeois, 1883), comb. nov. 

The Sri Lankan species *Xylobanomorphus villosus* (Kazantsev, 2006) was recently transferred from *Xylobanus* (Cautirina) to *Xylobanomorphus* Kleine, 1935 (Metriorrhynchina) [94] and it currently remains a single Metriorrhynchina species that occurs in a distant zoogeographic region without any contact with the center of the diversification of the group. Considering the uncertainty expressed by the author of the species when he transferred the species between subtribes, we do not intend to take any formal action, although we believe that the species belongs to *Xylobanus* and is possibly related to other small-bodied Cautirina with erected setae from this area (e.g., *X. hirtus* Kleine, 1928, and *Prometanoeus ochraceus* Kleine, 1925). 

### 3.2. Biodiversity and Zoogeography of the Metriorrhynchina

Metriorrhynchina, with 876 formally species described in 32 genera, is the highly diverse lineage of net-winged beetles [20], and it accounts for about three-quarters of the number of genera presently placed into Metriorrhynchini. It surpasses in species diversity its sister group, the subtribe Cautirina (~650 spp.) as well as all currently defined tribes of net-winged beetles [2]. The range of genera which presently form Metriorrhynchina [16,20] has been constantly overestimated as many Afrotropical and Asian species were incorrectly placed in *Cladophorus* and *Metriorrhynchus* [3,28]. Similarly, some Metriorrhynchina were described in the genera now placed in the Cautirina [69,93]. The range of Metriorrhynchina is critically reconsidered based on the studies of primary types and assembled material from the whole range (Figure 2). 

Unlike other species-rich groups (e.g., Platerodini, Lycini, Calochromini, and Calopterini) [95,96], most of the Metriorrhynchina diversity is concentrated in a relatively small area of New Guinea from where about half of the recognized species are known (Figure 2) [2]. As New Guinea is a tectonically young island [97], we may suppose that the substantial part of the Metriorrhynchina alpha diversity evolved in the recent 10 million years. The area of the highest diversity is tectonically formed by the northern edge of the Australian craton and the islands which formed in the contact zone between the Australian and Philippine plates [97]. These areas include the northern part of New Guinea, the Solomon Islands, most of the Moluccas, and the northern part of the Sulawesi. The exclusion of the species erroneously *Cautires* and *Xylobanus* affects the range of the Cautirina which is limited to the Oriental and Afrotropical region with only three species formally described *Xylobanus* from Sulawesi (see Supplementary Materials) [87].

The Australian fauna is restricted to at least seasonally humid ecosystems along the eastern coast with the diversity gradually decreasing from northern Queensland to the south and west of the continent (112 spp. in Queensland, but only 43 species in the south-eastern part of the continent). The Australian fauna is characterized by the dominance of several genera (*Porrostoma*, *Trichalus*, *Synchonnus*, and *Stadenus*), but only the last genus is endemic to Australia (Table 1). The dominance of these genera stands in contrast with the generic structure of New Guinean fauna, in which *Porrostoma* and *Synchonnus* are uncommon, *Stadenus* is absent, and most New Guinea species belong to *Cladophorus*, the *Cautiromimus*/*Ditua* complex, *Diatrichalus*, *Eniclases*, and *Microtrichalus*. *Metriorrhynchus* is widely distributed in both regions. The net-winged beetle fauna of Western Australia is limited by a pronounced dry season, and there have been reported only ten species from the state. The Northern Territory is also seasonally dry and is characterized by the low habitat diversity due to the absence of high mountains. As a result, lycids are uncommon in the region (four species). There have been 10 species reported from Tasmania, and, except one, all are endemic to the island. One species, very common in coniferous forests, was reported from New Zealand (*Porrostoma rufipennis* (F.)), but it was likely introduced from Australia [2]. The Australian fauna is insufficiently known as the latest taxonomic studies dealing with the local Metriorrhynchini fauna were published about 100 years ago, and, since then, only a single more extensive study brought a dozen new species [10]. 

Due to the absence of modern research, a portion of species is known only from original descriptions and ‘Australia’ or ‘New Hollandia’ are the only records that we have for their distribution (21 spp). Recently, some type specimens deposited in the Natural History Museum, London and Museums Victoria, Melbourne were studied, and the genera earlier known only from New Guinea were identified from Australia, such as *Diatrichalus*, *Microtrichalus*, and *Ditua* [39,98]. 

It is highly probable that further New Guinean genera will be found in north-eastern Australia, which was connected to New Guinea during the latest glacial maximum [99], but the climate was dry in eastern Australia when the sea levels were low [100], and such conditions likely limited the faunal exchange between Australia and New Guinea. The paleoclimate might also have negatively affected the ecosystems of northern Australia [100]. Nevertheless, the Australian Northern Territory likely served as a source area for the dispersal of *Trichalus communis* Waterhouse, 1879, the widespread species in the Asian region, which has its closest relative in Northern Australia and supposedly dispersed via the Lesser Sundas to the Great Sundas and Malaya [90]. 

The highest diversity of the Metriorrhynchina is known from New Guinea, especially the mountain ecosystems of the Central Cordillera, which are humid for the whole year and potentially provide favorable conditions for liquid sucking net-winged beetle larvae [18,25]. Since we found that New Guinea and its surrounding islands support 423 Metriorrhynchina species, it is highly probable that these represent only a fraction of the real diversity that has been shown in the recent taxonomic revisions of *Diatrichalus*, *Falsolucidota*, and *Eniclases* [39,45,71,75,101] and the high number of descriptions of new species from various genera by Kazantsev [6,7,8,9]. 

The New Guinean species are placed into 27 genera, and, of these, eleven genera are endemic to New Guinea. An additional three genera occur only in New Guinea and the Moluccas (Table 1). Despite a high genus-level endemism, the species placed in the endemic genera make up only <10% of the local alpha-diversity. All endemic genera are species-poor (≤4 spp.), except *Falsolucidota* (14 spp.). Further genera are most diverse in New Guinea, but they were able to colonize by a limited number of species the adjacent regions: *Ditua* Waterhouse, 1879 (10 spp. in New Guinea versus 2 sp. in other regions), *Cautiromimus* Kleine, 1926 (16 spp./1sp.), *Diatrichalus* (32 spp./17 spp.), *Flabellotrichalus* (12 spp./7 spp.), and *Eniclases* (36 spp./2 spp.). 

There are two genera that contain the large part of the New Guinean species: *Cladophorus* (111 spp.) and *Metriorrhynchus* (64 spp.). Both are widespread, but New Guinea is the center of their diversification. It is apparent that even such a relatively small landmass as New Guinea can house a high number of closely related species as has been found in earlier studies of other beetle groups [78,79]. 

Much less Metriorrhynchina occur in the Solomon Islands, New Britain, New Ireland, and the islands of Oceania (Woodlark, Marshal Islands, Vanikoro, Vanuatu; in total 21 described spp.) in agreement with other animal groups [81]. This fauna is derived from New Guinea, with which it shares all genera, but differs in the species composition except for a single unverified record of a species from both landmasses (see Supplementary Materials). The island faunas are species-poor as we can expect in poorly interconnected small landmasses [102]. The Oceanian fauna is poorly known, and further species will be surely discovered if new material is available from New Britain and New Ireland, which are close to New Guinea and sufficiently large and mountainous to house net-winged beetle fauna. 

Currently, only four species are known from New Britain and 12 species from the Solomon Islands. The oceanic islands located further to the east are small and very isolated and only a few species were described from them: one species from the Marshal Islands (Kio island in the Kwajalein atoll), one species from the Vanuatu island, two species from the Vanikoro island in the Santa Cruz group, and one species from the Woodlark Island (Papua New Guinea). The species of the region are placed in five genera (*Diatrichalus*, *Microtrichalus*, *Cautiromimus*, *Metriorrhynchus*, and *Cladophorus*), but the latter two genera have not been revised and the generic classification should be confirmed. A high proportion of species, seven of twenty-one species, belong to trichaline genera, which have been shown as effective dispersers [90]. 

One species of *Ditua* was collected in New Caledonia (G. Monteith, personal communication), but the species has not been described. The isolated islands housing lycid species are separated from the closest landmasses that could serve as a source area by up to 600 km of open sea. An outstanding record of a Metriorrhynchine species was reported from the Kio Island *Cladophorus planteni* Pic, 1923. The island lies ~2000 km from the nearest source area. The type of this species has not been found in Pic’s collection and possibly might represent a mislabeled specimen or the ‘Ile Kio’ given in Pic’s description might refer to a locality in a different region. Even if *C. planteni* is not considered, the putative dispersal events to Vanikoro, New Caledonia, and Vanuatu are exceptional compared to other net-winged beetles which seldom crossed the Makassar strait (the width ~100 km during the last glacial maximum, with two dispersal events hypothesized until now) and never crossed the Mozambique Strait (the width 560 km) [2,85,86,87]. 

All thirteen Metriorrhynchina genera recorded from the Moluccas occur also in New Guinea (Table 1), and there are no formally described species belong to endemic genera from the neighboring Sulawesi. We identified only a single species of undescribed *Leptotrichalus* in the entomological collection of the Indonesian Academy of Sciences in Cibinong; however, the material could not be made available for taxonomic study due to administrative restrictions, and the species has remained undescribed. Concerning the range of *Leptotrichalus*, the ancestor of the Halmaheran species very probably colonized the island from Sulawesi which lies about 240 km to the west of Halmahera, and, additionally, two islands, Maju and Gureda, are located in the strait between these landmasses, eventually from the Philippines in the north-west (460 km distance with Talau islands between the landmasses). 

A possible further connection between Sulawesi and Halmahera is represented by the chain of islands Pelei, Banggai, Sula, and Obi separated by a maximum oversea distance of ~100 km. All potential dispersal routes cross Weber’s line, and, as the Halmahera is a recently formed island and its distance from the Sulawesi and the Philippines was higher in the past, these potential dispersal routes have been available only for a short geological time [97]. The Moluccan fauna is highly endemic at the species but not at the generic level. There have been 54 species described from the area. The taxonomic research on these islands was neglected since the 1930′s, and only some species were described from Halmahera in the last decade [73,94]. The number of species will increase if alpha-taxonomic research is resurrected in the region. 

The fauna of Sulawesi is remarkably endemic at the generic level (4 of 10 genera) in agreement with other groups [81], and it is the only region with more endemic than non-endemic species (the Sulawesian endemics species account for >75% of the Metriorrhynchina fauna; 67 of 84 recorded spp.). The Metriorrhynchina of Sulawesi has been intensively studied only in the last two decades when several dozens of new species were reported mainly from mountain regions and three of four endemic genera were described [4,5,40,43,87]. The most diverse Sulawesian genera are *Broxylus*, *Wakarumbia*, and *Sulabanus* (11, 31, and 23 spp, respectively; Table 1), and all of them are endemic to Sulawesi at the current knowledge, although some *Sulabanus* might be identified among the Philippine *Xylobanus* in the future as is indicated in the description of some species [103]. 

The recent collecting activity was limited, and, undoubtedly, the Sulawesi fauna remains mostly unknown. Sulawesian *Leptotrichalus* fauna contains only six described species in Sulawesi, and it is the only Metriorrhynchina genus with the highest diversity east of Wallace’s line. Considering the distribution of the close relatives of *Leptotrichalus* (*Wakarumbia*) [20], which is endemic to Sulawesi, the phylogenetic relationships of Sulawesian *Leptotrichalus* should be studied, and dispersal routes should be investigated based on phylogenetic relationships. The current data on the structure of the Sulawesi fauna indicate partly its uniqueness and partly the function of the island as a stepping stone for the westward dispersal of Australian taxa [81,82,83] 

The Asian fauna consist of five genera and a limited number of species, most of them known from the Philippines (51 of 94 spp.). The earlier studies showed that Metriorrhynchina dispersed to South East Asia only in the last ~15 million years mostly through the Philippines (*Microtrichalus*, possibly *Leptotrichalus*, and the Philippine and Palawan species of *Metriorrhynchus)* [86,90]. The dispersal across the Makassar Strait was hypothesized only for *Metriorrhynchus* from the Great Sundas, Malaya, and Indo-Burma [85,86] in accordance with the earlier reported moderate permeability of this line for other lineages of beetles [82,83] or vertebrates, especially birds [81]. 

*Trichalus communis* is closely related to an Australian species and possibly colonized the Asian region via the Lesser Sundas [90], an area with a short tectonic history and so a less-employed dispersal route [97]. The Asian fauna must be a result of the westward crossing of isolating sea barriers as the biological connection between landmasses has been established quite recently and most genera of Metriorrhynchina occurring in the Oriental region are much more speciose in the regions east of the Wallace’s and Weber’s line. A similar bias for the higher diversity of New Guinean fauna and gradual decrease of diversity in the westward direction was identified also in weevils [82,83]. 

The dispersal in the opposite direction is much rarer, and Sulawesi obtained only a few species of Cautirina from the Great Sundas [87]. Neither *Cautires* nor *Xylobanus* dispersed further to the east as we did not identify a single species of these genera in the very extensive material that we studied [17,89] (the current study). Although Wallace’s line is an effective barrier for many animals [81], the quite frequent westward dispersal events have been identified also in other groups of insects in agreement with the westward direction in the Metriorrhynchina [83,84,90]. The northern limits of the Asian Metriorrhynchina are defined by a single report of an undescribed *Leptotrichalus* from the Shanghai area (unpublished record, the specimen deposited in Yun Li’s collection, Canberra) and by several records of *Microtrichalus* from southernmost China [98]. 

The westernmost record is represented by a *Metriorrhynchus* from India: Sylhet, which was incorrectly reported as *M. sericeus* Waterhouse, 1879, a species endemic to Java [3]. Without the voucher specimen(s), we are not able to identify which of two Indo-Burman *Metriorrhynchus* reached eastern India [86]. Despite the geographical proximity, the fauna of Taiwan does not contain any Metriorrhynchina species. The ~360 km wide Luzon strait was crossed even by the flightless weevils [104] but has prevented the flying and very common Philippine *Metriorrhynchus* and *Leptotrichalus* from dispersing further to the north. The observation indicates the low dispersal propensity of net-winged beetles even when compared with non-flying weevils [104]. 

**Figure 8:**
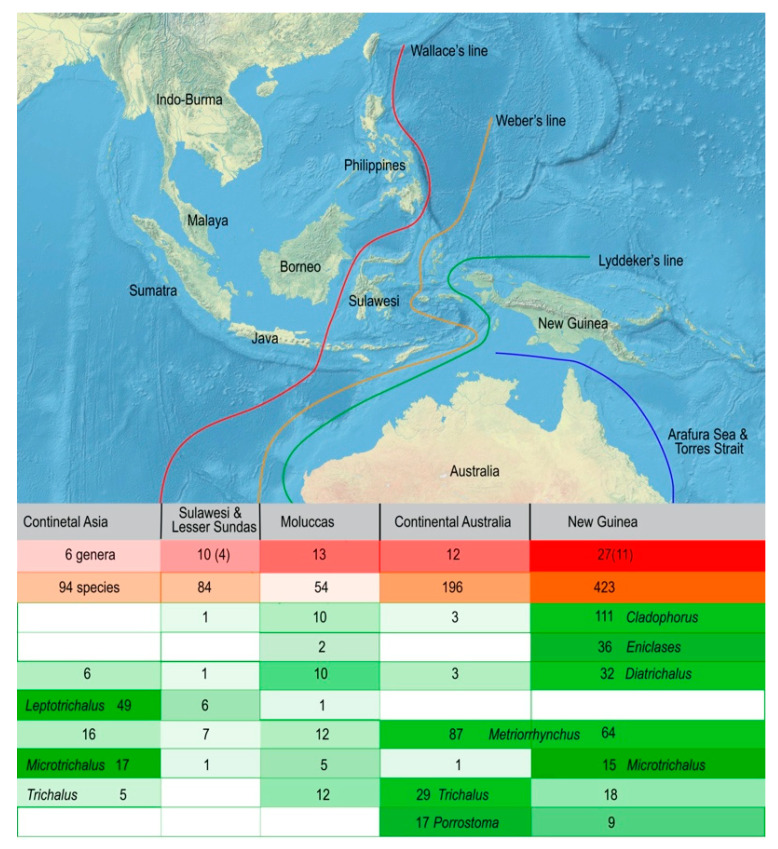
Overview of the dispersal barriers in continental Asia, Malesia, and Australia with the distribution summary of certain Metriorrhynchina genera. Almost all species occur only in a single area; only a single species has been reported from the regions separated by a zoogeographical line (*M. thoracicus*; Bocak & Matsuda 1998); however, the record from the Moluccas has never been confirmed, and it is possibly based on a mislabeled specimens used for the description of *M. cyaniventris* (Redtenbacher, 1867). The numbers of genera in parentheses refer to those endemic to the region.

Based on the distribution data, we can propose some groups of the Metriorrhynchina genera that share similar dispersal propensity and distribution patterns (Figure 8). Most genera are endemic to a single landmass, and, at least based on the current knowledge, they did not colonize neighboring regions. These include genera endemic to New Guinea (*Falsolucidota*, *Cladophorinus*, *Kassemia*, *Malacolycus*, etc.) and Sulawesi (*Broxylus*, *Wakarumbia*, etc.; Figure 6C), or they occur predominantly in one region and a few species (under 10% of the total diversity) colonized also an adjacent area: e.g., *Eniclases*, *Cautiromimus*, *Flabellotrichalus* (Table 1), and possibly *Sulabanus* from Sulawesi if the record from the Philippines is confirmed. 

The poorly dispersing species are highly diversified possibly due to low gene flow between species and populations isolated by a distance and geographical barriers, such as lowlands, and mountain ranges [24,75,105,106,107,108,109]. There are only a few Metriorrhynchina genera that were able to colonize landmasses separated by the sea straits after the distance between the Australian and Asian cratons decreased in the last 20 million years [97] and biological connections were established. *Metriorrhynchus* occurs in the almost whole range of the Metriorrhynchina (Figure 5 and Figure 8), it colonized South East Asia through the dispersal route across the Makassar Strait, and, from the Sulawesi, it also successfully colonized the Philippines [85,86]. 

Several genera from the trichaline clade similarly colonized the Asian region via the Philippines; however, no trichaline species was able to cross the Makassar strait, and the species reached the region either through the Lesser Sundas or the Moluccas-Philippines route [89,90]. These genera can be often collected on flowers in contrast with other net-winged beetles. At least in South East Asia, they often occur in lowlands, in the semidry habitats characterized by less a dense canopy, and they were observed flying in open situations (authors’ observations). 

## 4. Conclusions

Most taxonomic research in Metriorrhynchina was been based on taxonomic revisions, and the data on new species was accumulated as isolated descriptions with little reference to other species in the respective genera. Additionally, the nomenclatural ambiguities and unclear generic concepts caused further chaos [3,27]. Therefore, we reviewed the original descriptions, subsequent generic placements and distributional data, and we compiled the checklist of 876 Metriorrhynchina species. Numerous species earlier placed in Metriorrhynchine genera were transferred to Cautirina, further species were transferred among Metriorrhynchina genera, and new names were proposed to replace junior homonyms. 

The review of the diversity shows that Metriorrhynchina species usually occur in restricted regions, and we identified only a few species with ranges which included two landmasses separated by an open sea. We identified New Guinea and Sulawesi as two principal centers of endemism at the generic level in contrast with a single endemic genus in Australia. 

The diversity of Metriorrhynchina in New Guinea surpasses any other region of comparable size. The inventory of the Metriorrhynchina diversity and distribution data makes this group accessible for further research, both taxonomic and zoogeographic. The high species turnover makes Metriorrhynchina a promising model group for zoogeographical studies, which can identify the general dispersal routes across zoogeographic lines separating the unique faunas of Australia and Asia [110]. Although we attempted to rectify some classification and distribution errors, this is the first step that reduces uncertainty in the classification and zoogeography of Metriorrhynchina, and a good portion of work remains ahead as, in particular, the Australian fauna requires a thorough revision. 

## Figures and Tables

**Table 1:** The diversity and distribution of Metriorrhynchina genera.

	Total	SEA	Phil	Sul	LSun	Mol	NG	Sol	Aus
*Broxylus* Waterhouse, 1879	11			11 *					
*Cautiromimus* Kleine, 1926	17					1	16 #		
*Cladophorinus* Kleine, 1926	1						1 *		
*Cladophorus* Guérin-Méneville, 1830	131			1	1	10	111	5	3
*Diatrichalus* Kleine, 1926	49	1	5	1		3	32	4	3
*Ditua* Waterhouse, 1879	12						10	1	1
*Eniclases* Waterhouse, 1879	38					2	36 #		
*Falsolucidota* Pic, 1921	14						14 *		
*Flabellotrichalus* Pic, 1921	19					4	12		3
*Kassemia* Bocak, 1998	4						4 *		
*Leptotrichalus* Kleine, 1925	57	20	28	6	2				
*Lobatang* Bocak, 1998	7		1	1		1	4		
*Malacolycus* Kleine, 1943	2						2 *		
*Mangkutanus* Kubecek et al., 211	2			2 *					
*Marena* Kazantsev, 2007	3						3 *		
*Metriorrhynchoides* Kleine, 1926	4						4 *		
*Metriorrhynchus* Gemm. & Har., 1869	194	5	11	7		12	64	8	87
*Mimoxylobanus* Pic, 1921	2				1		1		
*Microtrichalus* Pic, 1921	43	12	5	1		5	16	3	1
*Oriomum* Bocak, 1999	1						1 *		
*Porrostoma* Laporte, 1838	26						9		17
*Procautires* Kleine, 1926	28					1	26		1
*Pseudodontocerus* Pic, 1921	4						4 *		
*Schizotrichalus* Waterhouse, 1879	2					1	1		
*Stadenus* Waterhouse, 1879	8								8 *
*Sulabanus* Dvorak & Bocak, 2007	23			23 *					
*Synchonnus* Waterhouse, 1879	22					1	5		16
*Trichalus* Waterhouse, 1877	65				1	12	18	0	29
*Wakarumbia* Bocak, 1999	31			31 *					
*Xylobanomimus* Kleine, 1926	1						1 *		
*Xylobanomorphus* Kleine, 1935	4						4 *		
*Xylothrix* Kazantsev, 2015	2						2 *		
*‘Xylobanus’* (species *incertae sedis*)	50					1	22		27
Total	876	43	51	84	5	54	423	21	196

Abbreviations for delimited regions. SEA—South East Asia; Sul—Sulawesi, LSun—the Lesser Sundas, Mol—the Moluccas, NG—New Guinea, Sol—the Solomon Islands and Oceania, and Aus—Australia. *—endemic to a region; #—over 90% of diversity known from a single region, recorded only from a single additional region.

## Supplementary Materials

The following files are available online at: https://www.mdpi.com/2075-4450/11/10/710/s1, Supplementary Text, The checklist of Metriorrhynchina genera and species.
