# Supplementary material for: Biodiversity Inventory and Distribution of Metriorrhynchina Net-Winged Beetles (Coleoptera: Lycidae), with the Identification of Generic Ranges
Source: Insects. 2020 Oct 16;11(10):710. doi: 10.3390/insects11100710 (PMC7603065; doi:10.3390/insects11100710)
Supplement: Supplementary file 1 [file insects-11-00710-s001.pdf]

The checklist of Metriorrhynchina genera and species

Family Lycidae Laporte, 1838
Subfamily Metriorrhynchinae Kleine, 1926a
Tribe Metriorrhynchini Kleine, 1926a
Subtribe Metriorrhynchina Kleine, 1926a

Metriorrhynchinae Kleine, 1926a: 97 (Type species. *Metriorhynchus* Guérin-Méneville, 1838 (cited as *Metriorrhynchus* in the original publication).

Metriorrhynchini Kleine, 1926a; Kleine, 1933a: 77; Bocak & Bocakova 2008: 716 (in Lycinae).

Metriorrhynchini Kleine, 1926a; Kazantsev, 2005: 77 (in Calochrominae).

Metriorrhynchina Kleine, 1926a; Sklenarova et al. 2014: 47.

=Cladophorinae Kleine, 1928c: 222 (Type genus. *Cladophorus* Guérin-Méneville, 1830); Bocak & Bocakova 1990a: 646.

=Dilolycinae Kleine, 1926a: 186 (Type genus. *Dilolycus* Kleine, 1926a); Bocak & Bocakova 1990a: 646.

=Haplothoracinae Kleine, 1926a: 95, *nomen nudum* (Type genus *Haplothorax* Kleine, 1926a, *nomen nudum*).

=Trichalinae Kleine, 1928c: 222 (Type genus. *Trichalus* Waterhouse, 1877).

=Trichalini Kleine, 1928c; Kleine, 1933a: 69.

=Trichalina Kleine, 1928c; Bocak & Bocakova 1990a: 646, synonymized by Sklenarova et al. 2014: 47.

=Hemiconderina Bocak & Bocakova, 1990: 645 (Type species Hemiconderis Kleine, 1926a); Sklenarova et al. 2014: 47.

Broxylus Waterhouse, 1879

Broxylus Waterhouse, 1879: 20 (Type species. *Calopteron pfeifferi* Waterhouse, 1878: 106; by monotypy).

=*Samanga* Pic, 1921: 9 (Type species. *Samanga fenestrata* Pic, 1921; by monotypy).

Broxylus pfeifferi (Waterhouse, 1878) – Sulawesi

Calopteron pfeifferi Waterhouse, 1878: 106.

Broxylus pfeifferae (Waterhouse, 1878); Waterhouse (1879: 21), Kleine (1933a: 35), incorrect subsequent spelling.

Broxylus pfeifferi (Waterhouse, 1878); Bocak & Jass 2004: 69.

Broxylus fenestratus (Pic, 1921) – Sulawesi

Samanga fenestrata Pic,1921a: 9.

Broxylus fenestratus Pic,1921a: 9; Bocak & Jass (2004: 70).

Broxylus jezeki Bocak & Jass, 2004: 70 – Sulawesi

Broxylus kalamensis Bocak & Jass, 2004: 72 – Sulawesi

Broxylus majeri Bocak & Jass, 2004: 69 – Sulawesi

Broxylus orobuensis Bocak & Jass, 2004: 74 – Sulawesi

Broxylus pendolensis Bocak & Jass, 2004: 72 – Sulawesi

Broxylus pseudofenestratus Bocak & Jass, 2004: 72 – Sulawesi

Broxylus tanatorajensis Bocak & Jass, 2004: 71 – Sulawesi

Broxylus malinensis Bocak & Jass, 2004: 71 – Sulawesi

Broxylus longicollis (Pic, 1922) – Sulawesi

Samanga longicollis Pic, 1922: 22.

Samanga longicollis (Pic, 1922); Bocak & Jass (2004: 74).

Cautiromimus Pic, 1926

Cautiromimus Pic, 1926a: 454 (Type species. *Cladophorus reticulatus* Kleine, 1926; by monotypy).

Cautiromimus amabilis (Waterhouse, 1884), **comb. nov.** – New Guinea: Aru Isl.

Cautires amabilis Waterhouse, 1884: 281.

Cautiromimus amabilis Waterhouse, 1884: 281; hereby proposed.

Cautiromimus atroscutus (Pic, 1922), **comb. nov.** – New Guinea

Cautires atroscutus Pic, 1922: 18.

Cautiromimus atroscutus (Pic, 1922); hereby proposed.

Cautiromimus elegans (Kleine, 1926a), **comb. nov.** – New Guinea

Cautires elegans Kleine, 1926a: 153.

Cautiromimus elegans (Kleine, 1926a); hereby proposed.

Cautiromimus facetus (Kleine, 1926a), **comb. nov.** – New Guinea

Cautires facetus Kleine, 1926a: 152.

Cautiromimus facetus (Kleine, 1926a); hereby proposed.

Cautiromimus factus (Kleine, 1926a), **comb. nov.** – New Guinea

Cautires factus Kleine, 1926a: 153.

Cautiromimus factus (Kleine, 1926a); hereby proposed.

Cautiromimus fuliginosus (Kleine, 1926a), **comb. nov.** – New Guinea

Cautires fuliginosus Kleine, 1926a: 155.

Cautiromimus fuliginosus (Kleine, 1926a); hereby proposed.

Cautiromimus insulanus (Kleine, 1926a), **comb. nov.** – New Britain

Cautires insulanus Kleine, 1926a: 154.

Cautiromimus insulanus (Kleine, 1926a); hereby proposed.

Cautiromimus kristinae (Kazantsev, 2010), **comb. nov.** – New Guinea

Cautires kristinae Kazantsev, 2010: 94.

Cautiromimus kristinae (Kazantsev, 2010); hereby proposed.

Cautiromimus maculosus (Kleine, 1926a), **comb. nov.** – New Guinea

Cautires maculosus Kleine, 1926a: 155.

Cautiromimus maculosus (Kleine, 1926a); hereby proposed.

Cautiromimus maturus (Kleine, 1926a), **comb. nov.** – New Guinea

Cautires maturus Kleine, 1926a: 150.

Cautiromimus maturus (Kleine, 1926a); hereby proposed.

Cautiromimus mediocris (Kleine, 1926a), **comb. nov.** – New Guinea

Cautires mediocris Kleine, 1926a: 151.

Cautiromimus mediocris (Kleine, 1926a); hereby proposed.

Cautiromimus mendicus (Kleine, 1926a), **comb. nov.** – New Guinea

Cautires mendicus Kleine, 1926a: 151.

Cautiromimus mendicus (Kleine, 1926a); hereby proposed.

Cautiromimus mendosus (Kleine, 1926a), **comb. nov.** – New Guinea

Cautires mendosus Kleine, 1926a: 153.

Cautiromimus mendosus (Kleine, 1926a); hereby proposed.

Cautiromimus mimicus (Kleine, 1926a), **comb. nov.** – New Guinea

Cautires mimicus Kleine, 1926a: 152.

Cautiromimus mimicus (Kleine, 1926a); hereby proposed.

Cautiromimus papuanus (Kleine, 1935a) – New Guinea

Bulenides papuanus Kleine, 1935a: 311.

Cautires papuanus Kleine, 1935a: 311; Dudkova & Bocak (2010: 45).

Cautiromimus reticulatus Kleine, 1926a – Moluccas: Bacan

Cladophorus reticulatus Kleine, 1926a: 143.

Cautiromimus reticulatus Kleine, 1926a: 143, Kleine (1933a: 58).

Cautiromimus socius (Kleine, 1935a) – New Guinea

Bulenides socius Kleine, 1935a: 311.

Cautiromimus socius (Kleine, 1935a); Dudkova & Bocak (2010: 45).

Cladophorinus Kleine, 1926

Cladophorinus Kleine, 1926a: 149 (Type species. *Cladophorinus cyaneus* Kleine, 1926a: 150; by original designation).

Cladophorinus cyaneus Kleine, 1926a: 149 – New Guinea

Cladophorus Guérin-Méneville, 1830

Lycus (Cladophorus) Guérin-Méneville, 1830: Plate II, Fig. 9 (Type species. *Cladophorus formosus* Guérin-Méneville, 1830; by subsequent designation).

Cladophorus Guérin-Méneville, 1830: Gemminger & Harold 1869: 1633.

= *Odontocerus* Guérin-Méneville 1838: 72 (Type species. *Cladophorus formosus* Guérin-Méneville, 1830; by subsequent designation).

= *Spacekia* Strand, 1936: 168, a junior objective homonym (Type species. *Cladophorus formosus* Guérin-Méneville, 1830).

Cladophorus (s. str.) aberrans Waterhouse, 1878: 112 –New Guinea: Aru Isl.

Cladophorus (s. str.) acutangulus Bourgeois, 1892: 513 – Moluccas: Obi Isl.; New Guinea: Aru Isl.

Cladophorus (s. str.) albocinctus Kleine, 1926a: 125 – New Guinea

Cladophorus (s. str.) amabilis Kleine, 1926a: 134 – New Guinea

Cladophorus (s. str.) andaiensis Pic, 1923a: 36 – New Guinea

Cladophorus (s. str.) angustifasciatus Kleine, 1935a: 319 – New Guinea

Cladophorus (s. str.) apicalis Macleay, 1886: 152 – New Guinea

Cladophorus (s. str.) argenteus Kleine, 1926a: 125 – New Guinea

Cladophorus (s. str.) atrolineatus (Pic, 1921) – New Guinea

Odontocerus atrolineatus Pic, 1921: 12.

Cladophorus (s. str.) atrolineatus (Pic, 1921); Kleine (1933a: 47).

Cladophorus (s. str.) basalis Kleine, 1926a: 127 – Moluccas: Ceram

Cladophorus (s. str.) basipennis Pic, 1923a: 37 – New Guinea

Cladophorus (s. str.) beccarii Bourgeois, 1892: 498 – New Guinea

Cladophorus (s. str.) bellus (Pic, 1923a) – New Guinea

Odontocerus bellus Pic, 1923a: 38.

Cladophorus bellus (Pic, 1923); Kleine (1933a: 47).

Cladophorus (s. str.) belzebuth Bourgeois, 1892: 502 – New Guinea

Cladophorus (s. str.) bicolor Kleine, 1926a: 129 – New Guinea

Cladophorus (s. str.) boceki Kalousova et al., 2017: 510 – New Guinea

Cladophorus (s. str.) brunneicornis Pic, 1923a: 36 – New Guinea

Cladophorus (s. str.) brunnescens Kleine, 1926a: 142 – New Guinea

Cladophorus (s. str.) buruanus Kleine, 1933b: 5 – Moluccas: Buru

Cladophorus (s. str.) cavifrons Kleine, 1935a: 312 – New Guinea

Cladophorus (s. str.) celebensis (Pic, 1921) – Sulawesi

Odontocerus celebensis Pic, 1921: 12.

Cladophorus celebensis (Pic, 1921); Kleine (1933a: 47).

Cladophorus (s. str.) cephalotes (Pic, 1923a) – New Guinea

Odontocerus cephalotes Pic, 1923a: 36.

Cladophorus cephalotes (Pic, 1923a); Kleine (1933a: 47).

Cladophorus (s. str.) ceramensis Kleine, 1926a: 137 – Moluccas: Ceram; New Guinea

Cladophorus (s. str.) cinctus (Waterhouse, 1877) – Australia: Queensland

Porrostoma cinctum Waterhouse, 1877: 80.

Metriorrhynchus cinctus (Waterhouse, 1879: 58); Lea (1909: 66).

Cladophorus cinctus (Waterhouse, 1877); Kleine (1933a: 47).

Cladophorus cinctum (Waterhouse, 1877); Calder (1998: 150).

=*Metriorrhynchus capucinus* Lea, 1899: 558; Kleine (1933a: 47).

Note. *C. cinctum* occurs only in Australia, the Kleine's (1933a) reports from Aru Isl., New Guinea, and Ternate are false.

Cladophorus (s. str.) collaris Guérin-Méneville, 1838: 73 – New Guinea

Cladophorus (s. str.) craterensis Kalousova et al., 2017: 511 – New Guinea

Cladophorus (s. str.) detractus Waterhouse, 1879: 64 – New Guinea

Cladophorus (s. str.) diabolus Bourgeois, 1892: 502 – Moluccas: Amboina

Cladophorus (s. str.) discobrunneus Pic, 1923a: 37 – New Guinea

Cladophorus (s. str.) diversicornis Pic, 1923a: 37 – New Guinea

Cladophorus (s. str.) divisus Kleine, 1935b: 177 – Solomon Isl.

Cladophorus (s. str.) dohertyi Pic, 1923a: 37 – New Guinea

Cladophorus (s. str.) dorsalis Bourgeois, 1892: 501 New Guinea: Aru Isl.

Cladophorus (s. str.) elegantulus Kleine, 1926a: 124 – New Guinea

Cladophorus (s. str.) emendatus Kleine, 1926a: 130 – New Guinea

Cladophorus (s. str.) exactus Kleine, 1926a: 126 – New Guinea
Cladophorus (s. str.) extraordinarius Kleine, 1926a: 138 – New Britain
Cladophorus (s. str.) fallax Kleine, 1926a: 139 – New Britain
Cladophorus (s. str.) flabellifer Bourgeois, 1892 – New Guinea

Cladophorus flabellifer Bourgeois, 1892: 496.

=*Cladophorus sellatus* Bourgeois, 1892: 498; Kleine, 1933a: 48.

Cladophorus (s. str.) flavofasciatus Kleine, 1939a: 110 – New Guinea
Cladophorus (s. str.) formosus Guérin-Méneville, 1838 – New Guinea

Cladophorus formosus Guérin-Méneville, 1838: 73.

=*Lycus (Cladophorus) dimidiatus* Guérin-Méneville, 1830: pl. 2, figure 9 (a junior objective homonym of *Lycus dimidiatus* Fabricius, 1801: 111).

Cladophorus (s. str.) francoisi Bourgeois, 1897: 72 – Oceania
Cladophorus (s. str.) fryi Kleine, 1930d: 410 – New Guinea
Cladophorus (s. str.) fuscatus Waterhouse, 1879: 64 – New Guinea
Cladophorus (s. str.) gagates Bourgeois, 1897: 72 – Oceania
Cladophorus (s. str.) gestroi Kleine, 1926a: 125 – New Guinea
Cladophorus (s. str.) grandis Kleine, 1939a: 109 – New Guinea
Cladophorus (s. str.) griseus Kleine, 1926a: 143 – New Guinea
Cladophorus (s. str.) haiaensis Kalousova et al., 2017: 511 – New Guinea
Cladophorus (s. str.) hirtus Kleine, 1935a: 314 – New Guinea
Cladophorus (s. str.) hospitus Kleine, 1935a: 312 – New Guinea
Cladophorus (s. str.) humboldti Pic, 1923a: 36 – New Guinea
Cladophorus (s. str.) humeralis Kleine, 1926a: 130 – New Guinea
Cladophorus (s. str.) immundus Kleine, 1926a: 140 – New Guinea
Cladophorus (s. str.) infernalis Bourgeois, 1892: 503 – New Guinea; Moluccas
Cladophorus (s. str.) inflabellatus Pic, 1923a: 36 – New Guinea
Cladophorus (s. str.) infrequens Kleine, 1939a: 110 – New Guinea
Cladophorus (s. str.) ingenuus Waterhouse, 1879: 64 – New Guinea
Cladophorus (s. str.) jobiensis (Pic, 1921) – Moluccas: Obi Isl.

Odontocerus jobiensis Pic, 1921: 12.

Cladophorus jobiensis (Pic, 1921); Kleine (1933a: 48).

Cladophorus (s. str.) kailakiensis Kalousova et al., 2017: 512 – New Guinea
Cladophorus (s. str.) kochi Kleine, 1926a: 137 – New Guinea
Cladophorus (s. str.) latithorax Kleine, 1930a: 343 – Timor
Cladophorus (s. str.) laudatus Kleine, 1935a: 311 – New Guinea
Cladophorus (s. str.) leopoldi Pic, 1932: 85 – New Guinea
Cladophorus (s. str.) longeflabellatus Pic, 1923a: 36 – New Guinea
Cladophorus (s. str.) longesuturalis Pic, 1923a: 37 – New Guinea
Cladophorus (s. str.) longicornis Macleay, 1886: 152 – New Guinea
Cladophorus (s. str.) loriae Kleine, 1926a: 127 – New Guinea
Cladophorus (s. str.) lucidus Kleine, 1926a: 132 – New Guinea
Cladophorus (s. str.) luctuosus Kleine, 1926a: 123 – New Guinea
Cladophorus (s. str.) luculentus Kleine, 1926a: 132 – New Guinea

Cladophorus (s. str.) luridus Kleine, 1926a: 131 – New Guinea

Cladophorus (s. str.) maculatithorax (Pic, 1923) – Moluccas: Obi Isl.

Odontocerus maculatithorax Pic, 1923a: 38.

Cladophorus maculatithorax (Pic, 1923a); Kleine (1933a: 49).

Cladophorus (s. str.) mancus Kleine, 1926a: 141 – Moluccas: Bacan Isl.

Cladophorus (s. str.) mandibularis Bourgeois, 1892: 507 – New Guinea

Cladophorus (s. str.) manokwarensis Kalousova et al., 2017: 513 – New Guinea

Cladophorus (s. str.) melaleucus Kleine, 1935a: 319 – New Guinea

Cladophorus (s. str.) metallicus Kleine, 1926a: 133 – New Guinea

Cladophorus (s. str.) micans Kleine, 1926a: 136 – New Guinea, Roon Isl.

Cladophorus (s. str.) mindikensis Kalousova et al., 2017: 513 – New Guinea

Cladophorus (s. str.) miniatus Macleay, 1887: 235 – Australia: Queensland

Cladophorus (s. str.) motykai Kalousova et al., 2017: 514 – New Guinea

Cladophorus (s. str.) nigrescens McLeay, 1886: 152 – New Guinea

Cladophorus (s. str.) nigriceps Kirsch, 1877: 143 – Moluccas: Halmahera, Buru; New Guinea, Aru Isl.

Cladophorus (s. str.) obsoletus Bourgeois, 1892: 513 – New Guinea

Cladophorus (s. str.) ochraceicollis Fairmaire, 1883: 22 – New Guinea: Duc de York Isl.

Cladophorus (s. str.) oculatus Kleine, 1926a: 126 – New Guinea

Cladophorus (s. str.) ornatus Waterhouse, 1879: 65 – New Guinea

Cladophorus (s. str.) pallens Kleine, 1926a: 124 – New Guinea

Cladophorus (s. str.) pallidulus Bourgeois, 1892: 505 – New Guinea

Cladophorus (s. str.) papuensis Bourgeois, 1892: 512 – New Guinea

Cladophorus (s. str.) piceoscutus Pic, 1923a: 37 – New Guinea, – Moluccas: Buru Isl.

Cladophorus (s. str.) pigidialis (Pic, 1923a) – New Guinea; Moluccas: Bacan Isl.

Odontocerus pigidialis Pic, 1923a: 38.

Cladophorus pigidialis (Pic, 1923a); Kleine (1933a: 50).

Cladophorus (s. str.) pilosus Kleine, 1926a: 136 – New Guinea

Cladophorus (s. str.) planteni Pic, 1923a: 37 – Oceania: Wake Isl., Marshal Isl.

Cladophorus (s. str.) praecipuus Kleine, 1926a: 188 – New Guinea

Cladophorus (s. str.) praemaculatus Kleine, 1939a: 111 – New Guinea

Cladophorus (s. str.) praetor Kleine, 1935b: 178 – New Guinea

Cladophorus (s. str.) prodigiosus Kleine, 1926a: 187 – New Guinea

Cladophorus (s. str.) psittacinus Kleine, 1935a: 312 – New Guinea

Cladophorus (s. str.) quadraticollis Bourgeois, 1892: 500 – New Guinea, Aru Isl.; Moluccas: Key Isl., Buru

Cladophorus (s. str.) restrictus Waterhouse, 1878: 113 – New Guinea, New Britain

=*Cladophorus tibialis* Bourgeois, 1892: 509; Kleine, 1933a: 50.

Cladophorus (s. str.) roonensis (Pic, 1921) – Moluccas: Roon Isl.

Odontocerus roonensis Pic, 1921: 12.

Cladophorus roonensis (Pic, 1921); Kleine (1933a: 50).

Cladophorus (s. str.) rubentipes Bourgeois, 1892: 506 – New Guinea, Aru Isl.

Cladophorus (s. str.) ruber Kleine, 1939a: 111 – New Guinea

Cladophorus (s. str.) rufithorax Bourgeois, 1892: 509 – New Guinea

Cladophorus (s. str.) scapularis Kleine, 1926a: 128 – New Guinea

Cladophorus (s. str.) semimarginatus Pic, 1923a: 37 – New Guinea

Cladophorus (s. str.) semirufus McLeay, 1886: 152 – New Guinea

Cladophorus (s. str.) septemareolatus Bourgeois, 1892: 511 – New Guinea: Aru Isl.

Cladophorus (s. str.) splendidus Kleine, 1935a: 314 – New Guinea

Cladophorus (s. str.) stygius Bourgeois, 1892: 503 – New Guinea, Aru Isl.; Moluccas: Buru

Cladophorus (s. str.) subdilutus (Pic, 1923a) – New Guinea

Odontocerus subdilutus Pic, 1923a: 38.

Cladophorus subdilutus (Pic, 1923a); Kleine (1933a: 51).

Cladophorus (s. str.) subflabellatus Pic, 1923a: 36 – New Guinea

Cladophorus (s. str.) subparallelus Pic, 1923a: 36 – New Guinea

Cladophorus (s. str.) subrutescens (Pic, 1923a) – New Guinea

Odontocerus subrutescens Pic, 1923a: 38.

Cladophorus subrutescens (Pic, 1923a); Kleine (1933a: 51).

Cladophorus (s. str.) tener Kleine, 1933b: 7 – Moluccas: Bacan

Cladophorus (s. str.) terminalis Kleine, 1935a: 313 – New Guinea

Cladophorus (s. str.) testaceipes Pic, 1923a: 37 – New Guinea

Cladophorus (s. str.) testaceocinctus Pic, 1923a: 37 – New Guinea

Cladophorus (s. str.) tricoloratus Kleine, 1943a: 150 – New Guinea

Cladophorus (s. str.) vicinus Pic, 1923a: 36 – New Guinea

Cladophorus (s. str.) victoriensis Pic, 1923b: 2 – Australia: Victoria

Cladophorus (s. str.) pallescens Kalousova et al., 2017: 514 – New Guinea

Cladophorus (s. str.) riedeli Kalousova et al., 2017: 515 – New Guinea

Cladophorus (s. str.) variabilis Kleine, 1935a: 313 – New Guinea

Cladophorus (s. str.) wallacei Kleine, 1933a: 8 – Moluccas

Cladophorus (s. str.) wasiorensis Kalousova et al., 2017: 519 – New Guinea

Subgenus *Falsocautires* Pic, 1926a

Subgenus *Falsocautires* Pic, 1926a: 452 [Type species. *Cladophorus (Falsocautires) batjanensis* Pic, 1926a].

Cladophorus (Falsocautires) batjanensis Pic, 1926a: 452 – Moluccas: Bacan

Diatrichalus **Kleine, 1926a**

Diatrichalus Kleine, 1926a: 167 (Type species *Diatrichalus xylobanoides* Kleine, 1926; by original designation).

=*Mimotrichalus* Pic, 1930: 92. (Type species. *Microtrichalus tenimberensis* Pic, 1930; by monotypy); Bocak, (1998a: 193).

Diatrichalus absonus (Kleine, 1926b: 108) – Philippines

Trichalus absonus Kleine, 1926b: 108.

Diatrichalus absonus (Kleine, 1926b); Bocak (2000a: 16).

Diatrichalus aeneus Bocak, 2001a: 7 – New Guinea

Diatrichalus aptus (Kleine, 1926a) – New Guinea: Aru Isl.

Trichalus aptus Kleine, 1926a: 175.

Diatrichalus aptus (Kleine, 1926a); Bocak (2001a: 30).

Diatrichalus assimilis (Kleine, 1926a) – New Guinea, New Britain

Trichalus assimilis Kleine, 1926a: 172.

Diatrichalus assimilis (Kleine, 1926a); Bocak (2001a: 30).

Diatrichalus bipunctatus Bocak, 2001a: 7 – New Guinea

Diatrichalus buruensis Bocek, 2017: 579 – New Guinea

Diatrichalus cerberus (Bourgeois, 1900) – New Guinea

Trichalus cerberus Bourgeois, 1900: 429.

Diatrichalus cerberus (Bourgeois, 1900); Bocak (2001a: 9).

=*Trichalus completus* Pic, 1923a: 36; Bocak (2001a: 9).

Diatrichalus clypeatus (Kleine, 1926b) – Philippines, Palawan

Trichalus clypeatus Kleine, 1926b: 107.

Diatrichalus clypeatus (Kleine, 1926b); Bocak (2000a: 13).

Diatrichalus cyanescens Bourgeois, 1900: 422 – New Guinea, Aru Isl.

Trichalus cyanescens, 1900: 422.

Diatrichalus cyanescens (Bourgeois, 1900); Bocak (2001a: 10).

Diatrichalus dentatus (Kleine, 1926a) – New Guinea

Trichalus dentatus Kleine, 1926a: 170.

Diatrichalus dentatus (Kleine, 1926a); Bocak (2001a: 11).

Diatrichalus dilatatus Bocak, 2001a: 13 – New Guinea

Diatrichalus emarginatus Bocak, 2001a: 14 – New Guinea

Diatrichalus fasciatus Bocak, 2001a: 14 – New Guinea

Diatrichalus fenestratus Bocak, 2001a: 15 – New Guinea

Diatrichalus funereus (Blackburn, 1900) – Australia: Victoria

Trichalus funereus Blackburn, 1900: 51.

Diatrichalus funereus (Blackburn, 1900); Bocak (2001a: 30).

Diatrichalus habbema Bocak, 2001a: 15 – New Guinea

Diatrichalus humeralis Bocak, 2001a: 17 – New Guinea

Diatrichalus insulanus (Kleine, 1933b) – Moluccas: Bacan

Trichalus insulanus Kleine, 1933b: 12.

Diatrichalus insulanus (Kleine, 1933b); Bocak (2001a: 30).

Diatrichalus kershawi (Lea, 1908) – Australia: Tasmania

Trichalus kershawi Lea, 1908: 159.

Diatrichalus kershawi (Lea, 1908); Bocak (2001a: 30).

Diatrichalus luzonicus (Kleine, 1929) – Philippines

Trichalus luzonicus Kleine, 1929: 485.

Diatrichalus luzonicus (Kleine, 1929); Bocak (2000a: 12).

Diatrichalus malcheri Kleine, 1939b: 590 – Solomon Isl.

Diatrichalus mancus Kleine, 1926a: 176 – New Guinea

Trichalus mancus Kleine, 1926a: 176.

Diatrichalus mancus (Kleine, 1926a); Bocak (2000a: 17).

Diatrichalus manokwarensis Bocek 2017: 580 – New Guinea

Diatrichalus metallicus (Kleine, 1935c) – New Guinea

Trichalus metallicus Kleine, 1935c: 151.

Diatrichalus metallicus Kleine, 1935c: 151; Bocak (2001a: 19).

Diatrichalus mindikensis Bocek 2017: 582 – New Guinea

Diatrichalus niger (Waterhouse, 1879: 71) – Sundas, Malaya

Trichalus niger Waterhouse, 1879: 71.

Diatrichalus niger Waterhouse, 1879: 71; Bocak (2001a: 30).

=*Metriorrhynchus specularis* Schaufuss, 1887: 130.

=*Trichalus specularis* (Schaufuss, 1887); Kleine (1933a: 72).

=*Trichalus concolor* Kleine, 1927a: 312; Bocak (2001a: 30).

=*Trichalus nigricolor* Pic, 1927: 42; Bocak (2001a: 30).

=*Trichalus niger* var. *notaticollis* Pic, 1927: 42; Bocak (2001a: 30).

=*Trichalus niger* var. *angustatus* Pic, 1927: 42; Bocak (2001a: 30).

Diatrichalus pallidihumeralis Bocak, 2001a: 19 – New Guinea

Diatrichalus parallelus Bocak, 2001a: 20 – New Guinea

Diatrichalus piper Bocak, 2001a: 21 – New Guinea

Diatrichalus proprius Bocak, 2001a: 21 – New Guinea

Diatrichalus puerilis (Kleine, 1935b) – Solomon Isl.

Flabellotrichalus puerilis Kleine, 1935b: 180.

Diatrichalus puerilis (Kleine, 1935b); Bocak (2001a: 30).

Diatrichalus rubricostatus Bocak, 2001a: 22 – New Guinea

Diatrichalus robustus Bocek, 2017: 582 – New Guinea

Diatrichalus ruficollis Bocak, 2000a: 15 – Philippines

Diatrichalus salomonensis (Kleine, 1933b) – Solomon Isl.

Flabellotrichalus salomonensis Kleine, 1933b: 13.

Diatrichalus salomonensis (Kleine, 1933b); Bocak (2001a: 30).

Diatrichalus schawalleri Bocak, 2001a: 23 – New Guinea

Diatrichalus secretus (Kleine, 1935a) – New Guinea

Trichalus secretus Kleine, 1935a: 317.

Diatrichalus secretus (Kleine, 1935a); Bocak (2001a: 24).

Diatrichalus sedlacekiae Bocak, 2001a: 24 – New Guinea

Diatrichalus semicostatus (Blackburn, 1892) – Australia: New South Wales Victoria

Trichalus semicostatus Blackburn, 1892: 525.

Diatrichalus semicostatus (Blackburn, 1892); Bocak (2001a: 31).

=*Trichalus raymondi* Lea, 1895: 600; Kleine, 1933a: 73.

Diatrichalus signatus (Kleine, 1939c) – Moluccas: Buru

Trichalus signatus Kleine, 1939c: 19.

Diatrichalus signatus (Kleine, 1939c); Bocak (2001a: 30).

Diatrichalus sinuaticollis (Pic, 1923a) – New Guinea

Trichalus sinuaticollis Pic, 1923a: 36.

Diatrichalus sinuaticollis (Pic, 1923a); Bocak (2001a: 25).

Diatrichalus striatus Bocak, 2001a: 25 – New Guinea

Diatrichalus subarcuatithorax (Pic, 1926), comb. nov. – New Guinea

Trichalus subarcuatithorax Pic, 1926: 454.

Diatrichalus subarcuatithorax Pic, 1926); hereby proposed.

=*Diatrichalus biroi* Kleine, 1943a: 152, **syn. nov.**; hereby proposed.

Diatrichalus tenimberensis (Pic, 1930) – Tanimbar

Mimotrichalus tenimberensis Pic, 1930: 92.

Diatrichalus tenimberensis (Pic, 1930); Bocak (2001a: 26).

Diatrichalus tigoplanus Bocak, 2000a: 13 – Palawan

Diatrichalus typicus (Kleine, 1939d) – Sulawesi

Trichalus typicus Kleine, 1939d: 133.

Diatrichalus typicus (Kleine, 1939d); Bocak (2001a: 31).

Diatrichalus vicarius (Kleine, 1935b) – Solomon Isl.

Flabellotrichalus vicarius Kleine, 1935b: 179.

Diatrichalus vicarius (Kleine, 1935b); Bocak (2001a: 31).

Diatrichalus wauensis Bocak, 2001a: 27 – New Guinea

Diatrichalus xylobanoides Kleine, 1926a: 167 – New Guinea

Ditua Waterhouse, 1879

Ditua Waterhouse, 1879: 33 (Type species. *Lycus* (genus 20) *deplanatus* Waterhouse, 1878: 100, 109; by monotypy).

Ditua alternata Bourgeois, 1892: 515 – New Guinea

Ditua balkei Kazantsev, 2016a: 241 – New Guinea

Ditua dichroma (Boisduval, 1835) – New Guinea, Aru Isl., New Britain

Calopteron dichroma Boisduval, 1835: 123.

Ditua dichroma (Boisduval, 1835); Bourgeois, 1900: 514.

Lycus deplanata Waterhouse, 1878: 100, 109.

Ditua deplanata (Waterhouse, 1878); Waterhouse, 1879: 34;

Ditua nigricornis Bourgeois, 1900: 514.

Ditua alternata Bourgeois, 1900: 515.

Ditua elevaticostata Kazantsev, 2016a: 244 – New Guinea

Ditua flavipes Bourgeois, 1892: 515 – New Guinea

Ditua nigra Kazantsev, 2016a: 241 – New Guinea

Ditua pectinicornis Lea, 1929, **comb. nov.** – Australia: Queensland, Northern Territories

Metriorrhynchus pectinicornis Lea, 1929: 339.

Porrostoma pectinicornis (Lea, 1929); Calder (1998: 160).

Ditua pectinicornis Lea, 1929, hereby transferred to *Ditua* Waterhouse, 1979

Ditua sinaievi Kazantsev, 2016a: 244 – New Guinea

Ditua spinifera Kazantsev, 2016a: 244 – New Guinea

Ditua telnovi Kazantsev, 2016a: 245 – New Guinea

Ditua tuzovi Kazantsev, 2016a: 247 – New Guinea

Ditua wooklarkiana (Montrouzier, 1857), **comb. nov.** – Woodlark Isl.

Eros woodlarkianus Montrouzier, 1857: 16.

Ditua wooklarkiana (Montrouzier, 1857); hereby proposed.

Eniclases **Waterhouse, 1879**

Eniclases Waterhouse, 1879: 66 (Type species. *Lycus luteolus* Waterhouse, 1878).

subgenus *Trichalolus* Pic, 1923a: 36; Bocak & Bocakova (1991: 206). (Type species. *Trichalus* (*Trichalolus*) *apertu*s Pic, 1923).

Eniclases apertus (Pic, 1923a: 36) – New Guinea

Trichalus (*Trichalolus*) *apertus* Pic, 1923a: 36.

= *Eniclases fumosus* Kleine, 1926a: 182; Bocak & Bocakova (1991: 217).

Eniclases bicolor Bocek & Bocak, 2016: 23 – New Guinea

Eniclases bokondinensis Bocek & Bocak, 2016: 26 – New Guinea

Eniclases brancuccii Bocek & Bocak, 2016: 25 – New Guinea

Eniclases divaricatus (Pic, 1921: 10) – New Guinea

Trichalus divaricatus Pic, 1921: 10.

Eniclases divaricatus (Pic, 1921); Bocak & Bocakova (1991: 212).

Eniclases efferatus Kleine, 1926a: 181 – New Guinea

Eniclases egregius Kleine, 1926a: 181 – New Guinea

Eniclases electus Kleine, 1926a: 182 – New Guinea

Eniclases elelimensis Bocek & Bocak, 2016: 26 – New Guinea

Eniclases flabellatus Bocak & Bocakova 1991: 207 – New Guinea

Eniclases flavoscutellaris Bocak & Bocakova 1991: 216 – New Guinea

Eniclases fuscicornis Bocak & Bocakova 1991: 208 – New Guinea

Eniclases infuscatus Bocek & Bocak, 2016: 23 – New Guinea

Eniclases kusyi Bocek & Adamkova 2019: 345 – Moluccas: Halmahera

Eniclases luteolus (Waterhouse, 1878) – New Guinea

Lycus luteolus Waterhouse, 1878: 118.

Eniclases luteolus (Waterhouse, 1878); Waterhouse (1879: 66).

Eniclases lutescens Pic, 1926: 454 – New Guinea

Eniclases moluccanus Kleine, 1930b: 328 – Moluccas: Halmahera

Eniclases nicricornis Bocak & Bocakova 1991: 216 – New Guinea

Eniclases niger Bocek & Bocak, 2016: 29 – New Guinea

Eniclases nigriceps Bocak & Bocakova 1991: 208 – New Guinea

Eniclases nigroruber Kleine, 1935a: 318 – New Guinea

Eniclases pallidus Bocak & Bocakova 1991: 209 – New Guinea

Eniclases papuensis Bocak & Bocakova 1991: 213 – New Guinea

Eniclases pectinicornis Bocak & Bocakova 1991: 211 – New Guinea

Eniclases proximus Bocek & Bocak, 2016: 29 – New Guinea

Eniclases pseudoapertus Bocek & Bocak, 2016: 21 – New Guinea

Eniclases pseudoluteolus Bocak & Bocakova 1991: 209 – New Guinea

Eniclases riedeli Bocak, 1998d: 14 – New Guinea
Eniclases robustus Bocak & Bocakova 1991: 209 – New Guinea
Eniclases sedlaceki Bocak & Bocakova 1991: 212 – New Guinea
Eniclases serratus Bocak & Bocakova 1991: 217 – New Guinea
Eniclases similis Bocak & Bocakova 1991: 210 – New Guinea
Eniclases slipinskii Bocak & Bocakova 1991: 213 – New Guinea
Eniclases subelectus Bocak & Bocakova 1991: 215 – New Guinea
Eniclases tikapurensis Bocek & Bocak, 2016: 24 – New Guinea
Eniclases variabilis Bocek & Bocak, 2016: 27 – New Guinea
Eniclases versicolor Kleine, 1926a: 182 – New Guinea
Eniclases wauensis Bocak & Bocakova 1991: 214 – New Guinea

***Falsolucidota* Pic, 1921**

Falsolucidota Pic, 1921: 9, hors texte, *F. testaceicollis* Pic, 1921: 9 (by monotypy).

Hemiconderis Kleine, 1926a: 162, *H. explicatus* Kleine, 1926a: 162 (by monotypy); Kazantsev (2009: 278).

Falsolucidota anthracina (Bocak, 1999a), **comb. nov.** – New Guinea

Hemiconderis anthracinus Bocak, 1999a: 168.

Falsolucidota anthracina Bocak, 1999a: 168; hereby proposed.

Falsolucidota bipustulata (Bocak & Bocakova, 1990b), **comb. nov.** – New Guinea

Hemiconderis bipustulatus Bocak & Bocakova, 1990b: 219.

Falsolucidota bipustulata (Bocak & Bocakova, 1990b); hereby proposed.

Falsolucidota bistriata (Bocak & Bocakova, 1990b), **comb. nov.** – New Guinea

Hemiconderis bistriatus Bocak & Bocakova, 1990b: 214.

Falsolucidota bistriata (Bocak & Bocakova, 1990b); hereby proposed.

Falsolucidota curticollis Kazantsev, 2016c: 357 – New Guinea
Falsolucidota explicata (Kleine, 1926a) – New Guinea

Hemiconderis explicatus Kleine, 1926a: 162.

Falsolucidota explicata (Kleine, 1926a); Kazantsev (2010: 278).

Falsolucidota niger (Bocak & Bocakova, 1990b), **comb. nov.** – New Guinea

Hemiconderis niger Bocak & Bocakova, 1990b: 213.

Falsolucidota niger (Bocak & Bocakova, 1990b); hereby proposed.

Falsolucidota pallidihumeralis (Bocak & Bocakova, 1990b), **comb. nov.** – New Guinea

Hemiconderis pallidihumeralis Bocak & Bocakova, 1990b: 215.

Falsolucidota pallidihumeralis (Bocak & Bocakova, 1990b); hereby proposed.

Falsolucidota paula Kazantsev, 2016c: 356 – New Guinea
Falsolucidota plannipennis Kazantsev, 2016c: 357 – New Guinea
Falsolucidota rajampatensis Kazantsev, 2016c: 359 – New Guinea
Falsolucidota samuelsoni (Bocak & Bocakova, 1990b), **comb. nov.** – New Guinea

Hemiconderis samuelsoni Bocak & Bocakova, 1990b: 217.

Falsolucidota samuelsoni (Bocak & Bocakova, 1990b); hereby proposed.

Falsolucidota sedlaceki (Bocak & Bocakova, 1990b), **comb. nov.** – New Guinea

Hemiconderis sedlaceki Bocak & Bocakova, 1990b: 215.

Falsolucidota sedlaceki (Bocak & Bocakova, 1990b); hereby proposed.

Falsolucidota suturalis (Bocak & Bocakova, 1990b), **comb. nov.** – New Guinea

Hemiconderis suturalis Bocak & Bocakova, 1990b: 217.

Falsolucidota suturalis (Bocak & Bocakova, 1990b); hereby proposed.

Falsolucidota telnovi Kazantsev, 2016c: 357 – New Guinea

Falsolucidota testaceicollis Pic, 1921: 9 – New Guinea: Roon

Flabellotrichalus Pic, 1921

Flabellotrichalus Pic, 1921: 9, as a subgenus of *Trichalus* Waterhouse, 1879 (Type species. *Flabellotrichalus* Pic, 1921: 9, by subsequent designation).

=*Stereotrichalus* Kleine, 1926a: 184 (Type species. *Stereotrichalus evidens* Kleine, 1926a: 184, by subsequent designation).

= *Villosotrichalu*s Pic, 1921: 9 (Type species. *Villosotrichalu*s *reductus* Pic, 1921: 9; by monotypy); Bocak (1998a: 184).

subgen. *Maibrius* Bocek et Bocak, 2017: 22 (Type species. *Flabellotrichalus (M.) horaki* Bocek et Bocak, 2017; by original designation).

Subgenus *Flabellotrichalus s. str.*

Flabellotrichalus (s. str.) bilineatus Pic, 1921: 9 – Moluccas: Bacan

Flabellotrichalus (s. str.) cheesmanni Kleine, 1935a: 317 – New Guinea

Flabellotrichalus (s. str.) crinitus (Kleine, 1926a), **comb. nov.** – New Guinea

Villosotrichalus crinitus Kleine, 1926a: 184.

Flabellotrichalus crinitus (Kleine, 1926a): hereby proposed.

Flabellotrichalus (s. str.) duplicatus Kleine, 1930b: 330 – Australia: West – Australia

Flabellotrichalus (s. str.) evidens Kleine, 1926a: 184 – New Guinea

Flabellotrichalus (s. str.) flabellatus (Bourgeois, 1900) – New Guinea

Trichalus flabellatus Bourgeois, 1900: 427.

Flabellotrichalus flabellatus (Bourgeois, 1900); Kleine (1926a: 184).

Flabellotrichalus (s. str.) flabellicornis Lea, 1909: 97 – Australia: Queensland

Trichalus flabellicornis Lea, 1909: 97.

Flabellotrichalus flabellicornis Lea, 1909a: 97; Kleine (1933a: 75).

Flabellotrichalus (s. str.) fuliginosus Kleine, 1926a: 185 – New Guinea

Flabellotrichalus (s. str.) incertus Kleine, 1935a: 318 – New Guinea

Flabellotrichalus (s. str.) notatithorax Pic, 1921: 9 – Moluccas: Bacan

Flabellotrichalus (s. str.) olivieri (Bourgeois, 1885) – Moluccas: Halmahera

Trichalus olivieri Bourgeois, 1885: 83.

Flabellotrichalus (s. str.) olivieri Bourgeois, 1885: 83; Kleine (1933a: 75).

Flabellotrichalus (s. str.) pilosus Kleine, 1926a: 185 – New Guinea

Flabellotrichalus (s. str.) reductus (Pic, 1921) – New Guinea

Villosotrichalus (s. str.) reductus Pic, 1921: 9.

Flabellotrichalus (s. str.) reductus (Pic, 1921); Bocak (1998a: 184).

Flabellotrichalus (s. str.) salomonensis Kleine, 1933b: 13 – Solomon Isl.
Flabellotrichalus (s. str.) semiatratus (Lea, 1909a) – Australia: Queensland

Trichalus semiatratus Lea, 1909a: 96.

Flabellotrichalus. (s. str.) semiatratus (Lea, 1909a: 96); Kleine (1933a: 79).

Flabellotrichalus (s. str.) talagaranus Kazantsev 2010a: 96 – Moluccas: Halmahera
Flabellotrichalus (s. str.) vicarius Kleine, 1935b: 179 – Solomon Isl.
Flabellotrichalus (s. str.) novaeguineensis Bocak, 1998b: 249 – New Guinea

=*Cladophorus dimidiatus* Bourgeois, 1892: 510 (a primary junior homonym of *Cladophorus dimidiatus* Guérin- Méneville, 1830: table 2, fig. 9).

Subgenus *Maibrius* Bocek & Bocak, 2017: 22
Flabellotrichalus (*Maibrius*) *horaki* Bocek & Bocak, 2017: 22 – New Guinea

***Kassemia* Bocak, 1998a**

Kassemia Bocak, 1998a: 195 (Type species. *Kassemia oculata* Bocak, 1998; by original designation).

Kassemia oculata Bocak, 1998a: 196 – New Guinea
Kassemia rufithorax Bocak, 1998a: 196 – New Guinea
Kassemia sedlacekiae Bocak, 1998a: 198 – New Guinea
Kassemia wauensis Bocak, 1998a: 197 – New Guinea

***Leptotrichalus* Kleine, 1925**

Leptotrichalus Kleine, 1925a: 296 (Type species. *Metriorrhynchus cyaniventris* Kirsch, 1875; by original designation).

Leptotrichalus acclinus Kleine, 1925: 300 – Philippines
Leptotrichalus accomodatus Kleine, 1925: 304 – Sulawesi
Leptotrichalus accuratus Kleine, 1925: 304 – Philippines
Leptotrichalus acerbus Kleine, 1925: 303 – Philippines
Leptotrichalus acidus Kleine, 1925: 302 – Philippines
Leptotrichalus acutus Kleine, 1925: 302 – Philippines
Leptotrichalus adjunctus Kleine, 1925: 307 – Philippines
Leptotrichalus adolescens Kleine, 1925: 305 – Philippines
Leptotrichalus admirabilis Kleine, 1925: 308 – Philippines
Leptotrichalus adversarius Kleine, 1925: 301 – Sumatra
Leptotrichalus angustatus (Pic, 1925a) – Philippines

Trichalus angustatus Pic, 1925a: 10.

Trichalus angustatus (Pic, 1925a); Kleine (1933a: 75).

Leptotrichalus apicalis Pic, 1927: 41 – Borneo: Banggi Isl.
Leptotrichalus atricollis (Pic, 1921) – Java

Trichalus atricollis Pic, 1921: 10.

Leptotrichalus atricollis Pic, 1921: 10; Kleine (1933a: 75).

Leptotrichalus bistriatus Bocak, 2000b: 181 – Sumatra

Leptotrichalus celebensis (Pic, 1921: 9) – Sulawesi

Trichalus celebensis Pic, 1921: 9.

Leptotrichalus celebensis Pic, 1921: 9; Kleine (1933a: 75).

Leptotrichalus chapaensis (Pic, 1923c), **comb. nov.** – Vietnam

Trichalus chapaensis Pic, 1923d: 54; Kleine (1933a: 70).

Leptotrichalus chapaensis (Pic, 1923); hereby proposed.

Leptotrichalus celsus Kleine, 1925: 301 – Philippines

Leptotrichalus cereus Kleine, 1925: 301 – Philippines

Leptotrichalus cicatricosus Kleine, 1925: 307 – Philippines

Leptotrichalus circumscriptus Kleine, 1925: 307 – Philippines

Leptotrichalus completus Kleine, 1925: 305 – Bali

Leptotrichalus compressipes (Pic, 1925) – Philippines

Trichalus compressipes Pic, 1925a: 10.

Leptotrichalus compressipes Pic, 1925a: 10; Kleine (1933a: 76).

Leptotrichalus conciliatus Kleine, 1925a: 299 – Java

Leptotrichalus concinnus Kleine, 1925: 306 – Philippines

Leptotrichalus cyaneiventris (Waterhouse, 1879) – Philippines

Trichalus cyaneiventris Waterhouse, 1879: 73.

Leptotrichalus cyaneiventris (Waterhouse, 1879): Kleine (1925: 300).

=*Trichalus subcyanescens* Pic, 1926b: 4, a junior objective synonym of *L. cyaneiventris* Waterhouse, 1879; Kleine (1933a: 76).

Note. The records of *L. cyaneiventris* from the Great Sundas and Malay Peninsula (Kleine, 1933a: 76) have not been confirmed and possibly referred to *L. cyaniventris* (Kirsch).

Leptotrichalus cyaniventris (Kirsch, 1875) – Great Sundas, Malaya

Metriorrhynchus cyaniventris Kirsch, 1875: 35.

Leptotrichalus cyaniventris (Kirsch, 1875); Kleine (1933a: 76).

Leptotrichalus cyaniventris cyaniventris (Kirsch, 1875); Bocak (2000b: 175).

=*Trichalus hypocrita* Bourgeois, 1892: 40; Kleine (1933a: 76).

=*Leptotrichalus basalis* Pic, 1929: 1; Bocak (2000b: 174).

=*Leptotrichalus pervicax* Kleine, 1939c: 17; Bocak (2000b: 175).

=*Leptotrichalus cyaniventris atrimembris* Pic, 1928: 32; Bocak (2000b: 175).

Leptotrichalus densereticulatus Kleine, 1939a: 17 – Sulawesi

Leptotrichalus dispar Kleine, 1926b: 104 – Philippines

Leptotrichalus dohertyi Pic, 1927: 41 – Borneo

Leptotrichalus effeminatus Kleine, 1925: 306 – Philippines

Leptotrichalus femoralis Kleine, 1925: 309 – Philippines

Leptotrichalus inapicalis Pic, 1927: 41 – Java

Leptotrichalus incertus Kleine, 1932a: 577 – Philippines: Luzon

Leptotrichalus insulanus Kleine, 1930b: 335 – Sulawesi: Great Sangir

Leptotrichalus javanus Kleine, 1927a: 313 – Java

Leptotrichalus linearis Kleine, 1928a: 325 – Borneo

Leptotrichalus longecostatus Pic, 1925a: 7 – Philippines: Luzon

Trichalus longicollis (Bourgeois, 1883), **comb. nov.** – Philippines

Trichalus longicollis Bourgeois, 1883: 649.

Leptotrichalus longicollis (Bourgeois, 1883); hereby proposed.

Leptotrichalus mindorosus (Pic, 1925) – Philippines

Trichalus mindorosus Pic, 1925a: 10.

Leptotrichalus mindorosus (Pic, 1925a); Kleine (1933a: 76).

Leptotrichalus nigricauda Bourgeois, 1886: 181 – Philippines: Luzon

Leptotrichalus pallidus (Dalman, 1818) – Sulawesi: Solo Isl.

Lycus pallidus Dalman in Schoenherr, 1818: 31.

Metriorrhynchus pallidus (Dalman, 1818); Kleine (1933a: 82).

Leptotrichalus pallidus (Dalman, 1818); Bocak et al. (2006: 125).

Leptotrichalus parvicostatus Kleine, 1930b: 336 – Lesser Sundas: Sumbawa

Leptotrichalus perversus Kleine, 1932: 575 – Philippines: Luzon

Leptotrichalus placitus Kleine, 1932: 576 – Philippines: Luzon

Leptotrichalus pubithorax (Pic, 1925) – Sumatra

Trichalus pubithorax Pic, 1925a: 8.

Leptotrichalus pubithorax (Pic, 1925a); Kleine (1933a: 76).

Leptotrichalus pullus Kleine, 1927: 313 – Java

Leptotrichalus reliquus Kleine, 1932: 576 Luzon

Leptotrichalus rubripennis (Pic, 1921) – Sumatra

Trichalus rubripennis Pic, 1921: 10.

Leptotrichalus rubripennis (Pica, 1921); Kleine (1933a: 77).

=*Trichalus pubithorax* Pic, 1925a: 8; Bocak (2000b: 178).

=*Leptotrichalus pubithorax* Pic, 1925a: 8; Kleine (1933a: 76).

=*Trichalus longecostatus* Pic, 1925a: 7; Bocak (2000b: 178).

=*Leptotrichalus longecostatus* Pic, 1925a: 7; Kleine (1933a: 76).

Leptotrichalus rufobasalis Pic, 1927: 41 – Java

Leptotrichalus setosus Kleine, 1927b: 2 – Philippines

Leptotrichalus shelfordi (Bourgeois, 1906) – Borneo

Trichalus shelfordi Bourgeois, 1906: 190.

Leptotrichalus shelfordi (Bourgeois, 1906); Kleine (1933a: 77).

=*Leptotrichalus rubrosignatus* Kleine, 1930c: 170; Bocak (2000b: 181).

=*Leptotrichalus reticulatus* Kleine, 1930b: 333; Bocak (2000b: 181).

Leptotrichalus similis Bocak, 2000b: 176 – Sumatra

Leptotrichalus subater (Pic, 1925a) – Philippines

Trichalus subater Pic, 1925a: 10.

Leptotrichalus subater (Pic, 1925a); Kleine (1933a: 77).

Leptotrichalus submarginatus Pic, 1921: 10 – Java

Leptotrichalus timorensis Kleine, 1930b: 333 – Timor

Leptotrichalus tolianus (Pic, 1925a) – Sulawesi

Trichalus tolianus Pic, 1925a: 10.

Leptotrichalus tolianus (Pic, 1925a); Kleine (1933a: 77).

Leptotrichalus tosarianus Kleine, 1931: 269 – Java

Lobatang Bocak, 1998a

Lobatang Bocak, 1998a: 190 (Type species. *L. papuensis* Bocak, 1998a: 190; by original designation).

Spinotrichalus Kazantsev 2010a: 92 (Type species. *S. telnovi* Kazantsev 2010a; by original designation).

subgenus *Spinotrichalus* Kazantsev 2010a: 92: Bocek & Bocak (2017: 20).

Lobatang s. str.

Lobatang (s. str.) *acuticornis* Bocak, 1998a: 192 – New Guinea

Lobatang (s. str.) *miserandus* (Kleine, 1939c) – Sulawesi

Trichalus miserandus Kleine, 1939c: 17.

Lobatang (s. str.) *miserandus* (Kleine, 1939c); Bocak (1998a: 192).

Lobatang (s. str.) *papuensis* Bocak, 1998a: 191 – New Guinea

Lobatang (s. str.) *sordescens* Bourgeois, 1900: 426 – New Guinea

Trichalus sordescens Bourgeois, 1900: 426.

Lobatang (s. str.) *sordescens* (Bourgeois, 1900); Bocak (1998a: 192).

Lobatang (s. str.) *flavohumeralis* Bocak & Bocakova 1999: 50 – New Guinea

Lobatang (s. str.) *dubitabilis* Kleine, 1925: 32 – Philippines

Leptotrichalus dubitabilis Kleine, 1925: 32.

Lobatang (s. str.) *dubitabilis* Kleine, 1925: 32; Bocak & Bocakova (1999: 51).

Spinotrichalus Kazantsev 2010a

Lobatang (*Spinotrichalus*) *telnovi* (Kazantsev 2010a: 93) – Moluccas: Halmahera

Malacolycus Kleine, 1943

Malacolycus Kleine, 1943: 151 (Type species. *Malacolycus paululus* Kleine, 1943: 151; by monotypy).

Malacolycus paululus Kleine, 1943a: 151 – New Guinea

Malacolycus lacius Kazantsev, 2015a: 113 – New Guinea

Mangkutanus Kubecek et al., 2011

Mangkutanus Kubecek et al., 2011: 648 (Type species. *Mangkutanus tenggahensis* Dvorak & Bocak, 2007; by original designation).

Mangkutanus tenggahensis Dvorak & Bocak, 2007 – Sulawesi

Sulabanus tenggahensis Dvorak & Bocak, 2007: 21.

Mangkutanus tenggahensis (Dvorak & Bocak, 2007); Kubecek et al. (2011: 648).

Sulabanus utarensis Dvorak & Bocak, 2007: 19 – Sulawesi

Sulabanus utarensis Dvorak & Bocak, 2007: 21.

Mangkutanus utarensis (Dvorak & Bocak, 2007); Kubecek et al. (2011: 650).

Marena Kazantsev, 2007

Marena Kazantsev, 2007: 297 (Type species. *Marena tristis* Kazantsev, 2007; by original designation).

Marena madangensis Kazantsev, 2007: 299 – New Guinea
Marena missai Kazantsev, 2007: 299 – New Guinea
Marena tristis Kazantsev, 2007: 299 – New Guinea

Metriorrhynchus Gemminger et Harold, 1869

Metriorrhynchus Gemminger et Harold 1869: 1629, unjustified emendation of *Metriorrhynchus* Guérin-Méneville, 1838 used as an available junior objective synonym by Bocak (1998b) (Type species. *Lycus parallelus* Guérin-Méneville, 1835: 114; by subsequent designation).
=*Metriorhynchus* Guérin-Méneville, 1838: 72, a junior homonym of *Metriorhynchus* Meyer, 1830 (Crocodylia) (Type species. *Lycus parallelus* Guérin-Méneville, 1835; by subsequent designation); Bocak, 1998b: 246.
=*Dilolycus* Kleine, 1926a: 186 (Type species. *Dilolycus lamellatus* Kleine, 1926a: 186; by original designation); Bocak (2002: 340).
=*Flabelloporrostoma* Pic, 1923a: 35, hors-texte (Type species. *Porrostoma mirabilis* Pic, 1923a: 35; by monotypy); Bocak (2002: 340).
Subgenus of *Porrostoma*: *Flabelloporrostoma* (Pic 1923a: 35); Calder (1998: 151).

Metriorrhynchus abdominalis (Waterhouse, 1877), **comb. nov.** – Australia: Queensland
Porrostoma abdominale Waterhouse, 1877: 7; Calder (1998: 152).
Metriorrhynchus abdominalis (Waterhouse, 1877); Lea, 1900: 64; hereby returned to *Metriorrhynchus* Gemminger & Harold, 1869.
Metriorrhynchus acutus Kleine, 1926a: 111 – New Guinea
Metriorrhynchus amplikefalus Kazantsev, 2015b: 213 – New Guinea
Metriorrhynchus angustatus Pic, 1923a: 39 – New Guinea
Metriorrhynchus angustulus Waterhouse, 1879: 57 – New Guinea
Metriorrhynchus angustus Lea, 1922, **comb. nov.** – Australia: Queensland
Metriorrhynchus angustus Lea, 1922: 3; hereby returned to *Metriorrhynchus* Gemminger & Harold, 1869.
Porrostoma angustus (Lea, 1922): Calder (1998: 152).
Metriorrhynchus antennatus Kleine, 1926a: 111 – New Britain
Metriorrhynchus apicivarius Lea, 1921a, **comb. nov.** – Australia
Metriorrhynchus apicivarius Lea, 1921a: 196; hereby returned to *Metriorrhynchus* Gemminger & Harold, 1869.
Porrostoma apicivarius (Lea, 1921a); Calder (1998: 152).
Metriorrhynchus apterus Lea, 1909, **comb. nov.** – Australia: Queensland
Metriorrhynchus apterus Lea, 1909: 72; hereby returned to *Metriorrhynchus* Gemminger & Harold, 1869.
Porrostoma apterus (Lea, 1909); Calder (1998: 152).
Metriorrhynchus ater Waterhouse, 1879: 57 – New Guinea, Aru Isl.
Metriorrhynchus atratus (Fabricius, 1801), **comb. nov.** – Australia: Tasmania
Lycus atratus Fabricius, 1801: 113.

Metriorrhynchus atratus (Fabricius, 1801); Kleine (1933a: 78; hereby returned to *Metriorrhynchus* Gemminger & Harold, 1869.

Porrostoma atratus (*Fabricius*, 1801); Calder (1998: 152).

Metriorrhynchus atroflavus Kleine, 1928b: 125 – Moluccas: Bacan

Metriorrhynchus basalis Bourgeois, 1911: 44 – New Britain

Metriorrhynchus batesi Lea, 1909, **comb. nov.** – Australia: New South Wales

Metriorrhynchus batesi Lea, 1909: 81; hereby returned to *Metriorrhynchus* Gemminger & Harold, 1869.

Porrostoma batesi (Lea, 1909): Calder (1998: 153).

Metriorrhynchus batjanensis Pic, 1921: 11 – Moluccas: Bacan

Metriorrhynchus boettcheri Kleine, 1926b: 80 – Philippines

Metriorrhynchus breveapicalis Pic, 1923b, **comb. nov.** – Australia: New South Wales

Metriorrhynchus breveapicalis Pic, 1923b: 3; hereby returned to *Metriorrhynchus* Gemminger & Harold, 1869.

Porrostoma breveapicalis (Pic, 1923b); Calder (1998: 153).

Metriorrhynchus brisbanensis Lea, 1909, **comb. nov.** – Australia: Queensland

Metriorrhynchus brisbanensis Lea, 1909: 75; hereby returned to *Metriorrhynchus* Gemminger & Harold, 1869.

Porrostoma brisbanensis (Lea, 1909); Calder (1998: 153).

Metriorrhynchus brunneoflavus Kazantsev, 2015b: 224 – New Guinea

Metriorrhynchus buergersi Kleine, 1926a: 103 – New Guinea

Metriorrhynchus caesareus Kleine, 1935b: 182 – Solomon Isl.

Metriorrhynchus centralis Macleay, 1887, **comb. nov.** – Australia: Queensland

Metriorrhynchus centralis Macleay, 1887: 230; hereby returned to *Metriorrhynchus* Gemminger & Harold, 1869.

Porrostoma centralis (Macleay, 1887); Calder (1998: 153).

Metriorrhynchus chamaeleon Kazantsev, 2015b: 219 – New Guinea

Metriorrhynchus chimaera Kazantsev, 2015b: 220 – New Guinea

Metriorrhynchus cliens Blackburn, 1900, **comb. nov.** – Australia: New South Wales

Metriorrhynchus cliens Blackburn, 1900: 53; hereby returned to *Metriorrhynchus* Gemminger & Harold, 1869.

Porrostoma cliens (Blackburn, 1900); Calder (1998: 153).

Metriorrhynchus compositus Lea, 1921b: 59, **comb. nov.** – Australia: New South Wales

Metriorrhynchus compositus Lea, 1921b: 59; hereby returned to *Metriorrhynchus* Gemminger & Harold, 1869.

Porrostoma compositus (Lea, 1921); Calder (1998: 153).

Metriorrhynchus connexus Lea, 1929, **comb. nov.** – Australia: Queensland

Metriorrhynchus connexus Lea, 1929: 335; hereby returned to *Metriorrhynchus* Gemminger & Harold, 1869.

Porrostoma connexus (Lea, 1929); Calder (1998: 153).

Metriorrhynchus costicollis Lea, 1921b, **comb. nov.** – Australia: Queensland

Metriorrhynchus costicollis Lea, 1921b: 84; hereby returned to *Metriorrhynchus* Gemminger & Harold, 1869.

Porrostoma costicollis (Lea, 1921b); Calder (1998: 153).

Metriorrhynchus crassipes Lea, 1921b, **comb. nov.** – Australia: Western, North Territory

Metriorrhynchus crassipes Lea, 1921b: 60; hereby returned to *Metriorrhynchus* Gemminger & Harold, 1869.

Porrostoma crassipes (Lea, 1921b); Calder (1998: 154).

Metriorrhynchus cribripennis Waterhouse, 1879: 52 – Moluccas

Note. Australia: New South Wales cited by error by Kleine (1933a: 81).

Metriorrhynchus croceus Kleine, 1926b: 79 – Philippines

Metriorrhynchus cryptoleucus Lea, 1921a, **comb. nov.** – Australia: Queensland

Metriorrhynchus cryptoleucus Lea, 1921a: 193; hereby returned to *Metriorrhynchus* Gemminger & Harold, 1869.

Porrostoma cryptoleucus (Lea, 1921a); Calder (1998: 153).

Metriorrhynchus decipiens Lea, 1929, **comb. nov.** – Australia: Queensland

Metriorrhynchus decipiens Lea, 1929: 336; hereby returned to *Metriorrhynchus* Gemminger & Harold, 1869.

Porrostoma decipiens (Lea, 1929); Calder (1998: 153).

Metriorrhynchus dentipes Lea, 1921a, **comb. nov.** – Australia: Queensland

Metriorrhynchus dentipes Lea, 1921a: 194; hereby returned to *Metriorrhynchus* Gemminger & Harold, 1869.

Porrostoma dentipes (Lea, 1921a); Calder (1998: 153).

Metriorrhynchus diffusimaculatus Kleine, 1928b, **comb. nov.** – Australia: Queensland

Metriorrhynchus diffusimaculatus Kleine, 1928b: 122; hereby returned to *Metriorrhynchus* Gemminger & Harold, 1869.

Porrostoma diffusimaculatus (Kleine, 1928b); Calder (1998: 153).

Metriorrhynchus dilutus Waterhouse, 1879: 58 – New Guinea: Mysol

Metriorrhynchus discolor Kleine, 1926a: 112 – New Guinea

Metriorrhynchus disconiger Lea, 1909, **comb. nov.** – Australia: Victoria

Metriorrhynchus disconiger Lea, 1909: 80; hereby returned to *Metriorrhynchus* Gemminger & Harold, 1869.

Porrostoma disconiger (Lea, 1909); Calder (1998: 153).

Metriorrhynchus dohertyi Pic, 1921: 11 – Moluccas: Bacan

Metriorrhynchus doleschali Redtenbacher, 1867: 101 – Moluccas

Note. There were not found any specimens of *M. doleschali* from Aru Isl. And Sulawesi as reported by Kleine, 1933a: 79.

Metriorrhynchus elongaticollis Pic, 1925a: 9 – Philippines: Mindanao

Metriorrhynchus elongatus Macleay, 1887, **comb. nov.** – Australia: Queensland

Metriorrhynchus elongatus Macleay, 1887: 229; hereby returned to *Metriorrhynchus* Gemminger & Harold, 1869.

Porrostoma elongatus (Macleay, 1887); Calder (1998: 153).

Metriorrhynchus elutus Kleine, 1926a: 100 – New Guinea, Aru Isl.

Metriorrhynchus ephippiger Guérin-Méneville, 1838: 73 – New Guinea

Metriorrhynchus eremitus Blackburn, 1900, **comb. nov.** – Australia: ACT, Victoria

Metriorrhynchus eremitus Blackburn, 1900: 55; hereby returned to *Metriorrhynchus* Gemminger & Harold, 1869.

Porrostoma eremita (Blackburn, 1900); Calder (1998: 153).

Metriorrhynchus eucerus Lea, 1921b, **comb. nov.** – Australia: Northern Territory

Metriorrhynchus eucerus Lea, 1921b: 60; hereby returned to *Metriorrhynchus* Gemminger & Harold, 1869.

Porrostoma eucerus (Lea, 1921b); Calder (1998: 154).

Metriorrhynchus excellens Kleine, 1926a: 98 – New Guinea

Metriorrhynchus exornatus Kleine, 1926a: 105 – New Guinea

Metriorrhynchus explanatus Kleine, 1926a: 105 – New Guinea

Metriorrhynchus faustus Kleine, 1926a: 113 – New Guinea

Metriorrhynchus femoralis Macleay, 1872, **comb. nov.** – Australia: Queensland

Metriorrhynchus femoralis Macleay, 1872: 262; hereby returned to *Metriorrhynchus* Gemminger & Harold, 1869.

Porrostoma femoralis (Macleay, 1872); 79; Calder (1998: 155).

Metriorrhynchus fessus Kleine, 1926a: 101 – New Guinea

Metriorrhynchus filirostris Lea, 1929, **comb. nov.** – Australia: Queensland

Metriorrhynchus filirostris Lea, 1929: 335; hereby returned to *Metriorrhynchus* Gemminger & Harold, 1869.

Porrostoma filirostris (Lea, 1929); Calder (1998: 155).

Metriorrhynchus flavicollis Waterhouse, 1879: 50 – Moluccas: Bacan

Metriorrhynchus flagellatus Blackburn, 1900, **comb. nov.** – Australian: New South Wales

Metriorrhynchus flagellatus Blackburn, 1900: 53; hereby returned to *Metriorrhynchus* Gemminger & Harold, 1869.

Porrostoma flagellatus (Blackburn, 1900); Calder (1998: 155).

Metriorrhynchus flavicostatus Kleine, 1926a: 103 – New Guinea

Metriorrhynchus flavipennis Lea, 1921a, **comb. nov.** – Australia

Metriorrhynchus flavipennis Lea, 1921a: 195; hereby returned to *Metriorrhynchus* Gemminger & Harold, 1869.

Porrostoma flavipennis (Lea, 1921a); Calder (1998: 155).

Metriorrhynchus flavoabdominalis Kleine, 1926a: 126 – Moluccas: Bacan

Metriorrhynchus flavofemoralis Kleine, 1926a: 186 – Solomon Isl.: Bougainville

Metriorrhynchus flavolateralis Waterhouse, 1879: 56 – New Guinea: Aru

Metriorrhynchus flavolimbatus Lea, 1921a, **comb. nov.** – Australia

Metriorrhynchus flavolimbatus Lea, 1921a: 191; hereby returned to *Metriorrhynchus* Gemminger & Harold, 1869.

Porrostoma flavolimbatus (Lea, 1921a); Calder (1998: 155).

Metriorrhynchus foliatus Macleay, 1887, **comb. nov.** – Australia: Queensland

Metriorrhynchus foliatus Macleay, 1887: 230; hereby returned to *Metriorrhynchus* Gemminger & Harold, 1869.

Porrostoma foliatus (Macleay, 1887); Calder (1998: 155).

Metriorrhynchus forcipatus Kleine, 1926b: 79 – Philippines

Metriorrhynchus formosus Kleine, 1926a: 113 – Solomon Isl.

Metriorrhynchus franklinmuelleri Kleine, 1928b, **comb. nov.** – Australia: Queensland

Metriorrhynchus franklinmuelleri Kleine, 1928b: 121.

Porrostoma franklinmuelleri (Kleine, 1928b); Calder (1998: 155).

Metriorrhynchus frater Lea, 1921a, **comb. nov.** – Australia

Metriorrhynchus frater Lea, 1921a: 192; hereby returned to *Metriorrhynchus* Gemminger & Harold, 1869.

Porrostoma frater (Lea, 1921a); Calder (1998: 155.)

Metriorrhynchus fuliginosus Kleine, 1930d: 409 – New Guinea

Metriorrhynchus fuligineus Lea, 1921b, **comb. nov.** – Australia

Metriorrhynchus fuligineus Lea, 1921b: 55; hereby returned to *Metriorrhynchus* Gemminger & Harold, 1869.

Porrostoma fuligineus (Lea, 1921b); Calder (1998: 156).

Metriorrhynchus pseudofunestus Bocak et al., 2020, **nom. nov**. – Australia

Metriorrhynchus funestus Lea, 1921b: 58 (a junior homonym of *Metriorrhynchus funestus* (Guérin-Méneville, 1838); hereby returned to *Metriorrhynchus* Gemminger & Harold, 1869.

Porrostoma funestus (Lea, 1921b); Calder (1998: 156).

Metriorhynchus funestus Guérin-Méneville, 1838: 73).

Metriorrhynchus funestus (Guérin-Méneville, 1838); Gemminger & Harold (1869: 1629).

Metriorrhynchus pseudofunestus Bocak et al., 2020 (a new replacement name) for *Metriorrhynchus funestus* (Guérin-Méneville, 1838).

Metriorrhynchus gloriosus Kleine, 1926a: 109 – Moluccas: Morotai

Metriorrhynchus gracilis Lea, 1909, **comb. nov.** – Australia: Queensland

Metriorrhynchus gracilis Lea, 1909: 83; hereby returned to *Metriorrhynchus* Gemminger & Harold, 1869.

Porrostoma gracilis (Lea, 1909); Calder (1998: 156).

Metriorrhynchus gratus Kleine, 1926a: 102 – New Guinea

Metriorrhynchus guttatus Kleine, 1926a: 103 – New Guinea

Metriorrhynchus hackeri Kleine, 1928b, **comb. nov.** – Australia: Queensland

Metriorrhynchus hackeri Kleine, 1928b: 121; hereby returned to *Metriorrhynchus* Gemminger & Harold, 1869.

Porrostoma hackeri (Kleine, 1928b); Calder (1998: 156).

Metriorrhynchus hexastichus Lea, 1921c, **comb. nov.** – Australia: New South Wales

Metriorrhynchus hexastichus Lea, 1921c: 351; hereby returned to *Metriorrhynchus* Gemminger & Harold, 1869.

Porrostoma hexastichus (Lea, 1921c); Calder (1998: 156).

Metriorrhynchus hirtipes Macleay, 1887, **comb. nov.** – Australia: Queensland

Metriorrhynchus hirtipes Macleay, 1887: 231; hereby returned to *Metriorrhynchus* Gemminger & Harold, 1869.

Porrostoma hirtipes (Macleay, 1887); Calder (1998: 156).

Metriorrhynchus immersus Waterhouse, 1879: 53 – New Guinea

Metriorrhynchus impudens Kleine, 1926a: 106 – New Guinea

Metriorrhynchus inaequalis (Fabricius, 1801) – Sumatra, Borneo, Malaya

Lycus inaequalis Fabricius, 1801: 112.

Metriorrhynchus inaequalis (Fabricius, 1801: 112); Waterhouse (1879: 51).

=*Metriorrhynchus notaticollis* Pic, 1921: 11; Kleine (1933a: 81).

Metriorrhynchus infuscatus Macleay, 1886 – New Guinea

Metriorrhynchus insignicornis Lea, 1921b, **comb. nov.** – Australia

Metriorrhynchus insignicornis Lea, 1921b: 56; hereby returned to *Metriorrhynchus* Gemminger & Harold, 1869.

Porrostoma insignicornis (Lea, 1921b); Calder (1998: 157).

Metriorrhynchus insignipes Lea, 1929: 337, **comb. nov.** – Australia

Metriorrhynchus insignipes Lea, 1929: 337; hereby returned to *Metriorrhynchus* Gemminger & Harold, 1869.

Porrostoma insignipes (Lea, 1929); Calder (1998: 157).

Metriorrhynchus intricatus Kleine, 1928b: 126 – Moluccas: Bacan

Metriorrhynchus isarogensis Bocak, Matsuda, Yagi, 2006: 120 – Philippines

Metriorrhynchus kingensis Lea, 1908, **comb. nov.** – Australia: Tasmania: King Isl.

Metriorrhynchus kingensis Lea, 1908: 160; hereby returned to *Metriorrhynchus* Gemminger & Harold, 1869.

Porrostoma kingensis (Lea, 1908); Calder (1998: 157).

Metriorrhynchus lamellatus (Kleine, 1926a) – New Guinea

Dilolycus lamellatus Kleine, 1926a: 186.

Metriorrhynchus lamellatus (Kleine, 1926a); Bocak (2002: 340).

Metriorrhynchus laosensis Bocak, 2007 – Laos

Metriorrhynchus lateanticus Kazantsev, 2015b: 221 – New Guinea

Metriorrhynchus lateratius Lea, 1921c, **comb. nov.** – Australia: Queensland

Metriorrhynchus lateratius Lea, 1921c: 353; hereby returned to *Metriorrhynchus* Gemminger & Harold, 1869.

Porrostoma lateratius (Lea, 1921c); Calder (1998: 157).

Metriorrhynchus lineatus (Kirsch, 1875) – Sumatra, Borneo, Malaya

Dictyopterus lineatus Kirsch, 1875: 35.

Metriorrhynchus lineatus Kirsch, 1875; Kleine (1933a: 81).

=*Metriorrhynchus enganoensis* Pic, 1921: 11; Kleine (1933a: 81) as a synonym of *M. inaequalis* (F.); Bocak (2007: 256).

=*Metriorrhynchus kirschii* Waterhouse, 1879: 50; Bocak (2007: 256).

=*Metriorrhynchus enganoensis* Pic, 1921: 11; Kleine, 1933a: 81; as a synonym of *M. inaequalis* (F.); Bocak (2007: 256).

=*Metriorrhynchus enganicus* Kleine, 1939c: 5; Bocak (2007: 256).

=*Metriorrhynchus regularis* Pic, 1921: 11; Bocak (2007: 256).

=*Metriorrhynchus dives* Kleine, 1927a: 310; Bocak (2007: 256).

Metriorrhynchus leucomelas Kleine, 1935c: 152 – New Guinea

Metriorrhynchus lobatus Bocak & Matsuda, 1998: 412 – Sulawesi

Metriorrhynchus longepilosus Kleine, 1928b, **comb. nov.** – Australia: Queensland

Metriorrhynchus longepilosus Kleine, 1928b: 122; hereby returned to *Metriorrhynchus* Gemminger & Harold, 1869.

Porrostoma longepilosus (Kleine, 1928b); Lea (1921c: 353).

Metriorrhynchus longicollis Lea, 1929, **comb. nov.** – Australia

Metriorrhynchus longicollis Lea, 1929: 338; hereby returned to *Metriorrhynchus* Gemminger & Harold, 1869.

Porrostoma longicollis (Lea, 1929); Calder (1998: 157).

Metriorrhynchus longicornis Macleay, 1887: 232 – Australia: Queensland

Metriorrhynchus longissimus (Pic, 1922) – Sulawesi

Xylobanus longissimus Pic, 1922: 16.

Metriorrhynchus longissimus (Pic, 1922); Bocak et al. (2006: 125).

Metriorrhynchus longulus Kleine, 1926a: 101 – New Guinea

Metriorrhynchus lorentzi Kleine, 1926a: 109 – New Guinea, New Britain, Moluccas: Halmahera

Metriorrhynchus loricatus Kleine, 1926a: 99 – New Guinea

Metriorrhynchus lunatus Kleine, 1926a: 107 – New Guinea

Metriorrhynchus luteobrunneus Bourgeois, 1889: 236 – Moluccas: Amboina

Metriorrhynchus macphersonensis Calder, 1998, **comb. nov.** – Australia: Queensland

Porrostoma macphersonensis Calder, 1998: 158, a replacement name.

Metriorrhynchus macphersonensis (Calder (1998); hereby proposed.

=*Metriorrhynchus hackeri* Lea, 1929: 337 (a junior homonym of *Metriorrhynchus hackeri* Kleine, 1928).

Metriorrhynchus maculithorax Kleine, 1926a: 127 – Moluccas: Bacan
Metriorrhynchus marginatus Kleine, 1926a: 102 – Sulawesi: Talaud Isl.
Metriorrhynchus marginipennis Lea, 1899, **comb. nov.** – Australia: New South Wales

Metriorrhynchus marginipennis Lea, 1899: 556; hereby returned to *Metriorrhynchus* Gemminger & Harold, 1869.

Porrostoma marginipennis (Lea, 1899); Calder (1998: 158).

Metriorrhynchus martini (Pic, 1925a) – Moluccas: Buru (2)

Odontocerus martini Pic, 1925a: 18.

Metriorrhynchus martini (Pic, 1925a); Kleine (1933a: 81).

Metriorrhynchus menieri Bocak, Matsuda, Yagi, 2006: 121 –Philippines
Metriorrhynchus mentior Blackburn, 1900, **comb. nov.** – Australia: Victoria

Metriorrhynchus mentior Blackburn, 1900: 54; hereby returned to *Metriorrhynchus* Gemminger & Harold, 1869.

Porrostoma mentior (Blackburn, 1900); Calder (1998: 158).

Metriorrhynchus militaris Lea, 1909, **comb. nov.** – Australia: New South Wales

Metriorrhynchus militaris Lea, 1909: 85; hereby returned to *Metriorrhynchus* Gemminger & Harold, 1869.

Porrostoma militaris (Lea, 1909); Calder (1998: 158).

Metriorrhynchus mindanaoensis Bocak, Matsuda, Yagi, 2006: 122 – Philippines
Metriorrhynchus minor Lea, 1921b, **comb. nov.** – Australia: Queensland, New South Wales

Metriorrhynchus minor Lea, 1921b: 54; hereby returned to *Metriorrhynchus* Gemminger & Harold, 1869.

Porrostoma minor (Lea, 1921b); Calder (1998: 158).

Metriorrhynchus minutus Lea, 1921b, **comb. nov.** – Australia

Metriorrhynchus minutus Lea, 1921b: 54; hereby returned to *Metriorrhynchus* Gemminger & Harold, 1869.

Porrostoma minutus (Lea, 1921b); Calder (1998: 158).

Metriorrhynchus mirabilis (Pic, 1923a), **comb. nov.** – Australia: New South Wales

Flabelloporrostoma mirabilis Pic, 1923a: 35; Calder (1998: 151).

Porrostoma (*Flabelloporrostoma*) *mirabilis* (Pic, 1923a); Calder (1998: 151).

Metriorrhynchus mirabilis (Pic, 1923a); Bocak, 2002: 340).

Metriorrhynchus moerens Lea, 1909, **comb. nov.** – Australia: Tasmania

Metriorrhynchus moerens Lea, 1909: 79; hereby returned to *Metriorrhynchus* Gemminger & Harold, 1869.

Porrostoma moerens (Lea, 1909); Calder (1998: 159).

Metriorrhynchus modicus Lea, 1921b, **comb. nov.** – Australia

Metriorrhynchus modicus Lea, 1921b: 59; hereby returned to *Metriorrhynchus* Gemminger & Harold, 1869.

Porrostoma modicus (Lea, 1921b); Calder (1998: 158).

Metriorrhynchus mollicollis Lea, 1929, **comb. nov.** – Australia: Queensland, New South Wales

Metriorrhynchus mollicollis Lea, 1929: 338; hereby returned to *Metriorrhynchus* Gemminger & Harold, 1869.

Porrostoma mollicollis (Lea, 1929); Calder (1998: 159).

Metriorrhynchus moluccanus Kleine, 1926a: 127 – Moluccas: Bacan
Metriorrhynchus monticola Blackburn, 1892, **comb. nov.** – Australia: Victoria

Metriorrhynchus monticola Blackburn, 1892: 526; hereby returned to *Metriorrhynchus* Gemminger & Harold, 1869.

Porrostoma monticola (Blackburn, 1892); Calder (1998: 159).

Metriorrhynchus montiphilus Kleine, 1926a: 111 – New Guinea

Metriorrhynchus newbataanensis Bocak, Matsuda, Yagi, 2006: 123 – Philippines

Metriorrhynchus nigricauda Kleine, 1928b, **comb. nov.** – Australia: Queensland

Metriorrhynchus nigricauda Kleine, 1928b: 123; hereby returned to *Metriorrhynchus* Gemminger & Harold, 1869.

Porrostoma nigricauda (Kleine, 1928b); Calder (1998: 159).

Metriorrhynchus nigripes Macleay, 1872, **comb. nov.** – Australia

Metriorrhynchus nigripes Macleay, 1872: 262; hereby returned to *Metriorrhynchus* Gemminger & Harold, 1869.

Porrostoma nigripes (Macleay, 1872); Calder (1998: 159).

=*Porrostoma elegans* Waterhouse, 1877: 75; Kleine (1933a: 82).

Metriorrhynchus nobilis Waterhouse, 1879: 54 – New Guinea

Metriorrhynchus occidentalis Blackburn, 1892, **comb. nov.** – Australia: West Australia

Metriorrhynchus occidentalis Blackburn, 1892: 526; hereby returned to *Metriorrhynchus* Gemminger & Harold, 1869.

Porrostoma occidentalis (Blackburn, 1892); Calder (1998: 159).

Metriorrhynchus ochropterus Kleine, 1935c: 153 – New Guinea

Metriorrhynchus ochii Bocak, Matsuda, Yagi, 2006: 123 – Philippines

Metriorrhynchus olivieri Kazantsev, 2015b: 213 – New Guinea

Metriorrhynchus opacus Lea, 1909, **comb. nov.** – Australia: Queensland

Metriorrhynchus opacus Lea, 1909: 89; hereby returned to *Metriorrhynchus* Gemminger & Harold, 1869.

Porrostoma opacus (Lea, 1909); Calder (1998: 159).

Metriorrhynchus ordinarius Lea, 1909, **comb. nov.** – Australia: Victoria

Metriorrhynchus ordinarius Lea, 1909: 81; hereby returned to *Metriorrhynchus* Gemminger & Harold, 1869.

Porrostoma ordinarius (Lea, 1909); Calder (1998: 159).

Metriorrhynchus pagdeni Kleine, 1935b: 182 – Solomon Isl.

Metriorrhynchus palawanensis Bocak, Matsuda, Yagi, 2006: 124 – Philippines

Metriorrhynchus pallidominor Lea, 1921b, **comb. nov.** – Australia: Northern Territory

Metriorrhynchus pallidominor (Lea, 1921b); hereby returned to *Metriorrhynchus* Gemminger & Harold, 1869.

Porrostoma pallidominor Lea, 1921b: 57; Calder (1998: 158).

Metriorrhynchus pallidipes Kazantsev, 2015b: 222 – New Guinea

Metriorrhynchus papuensis Macleay, 1884: 704 – New Guinea

Metriorrhynchus parallelus Guérin-Méneville, 1835 – New Guinea

Lycus parallelus Guérin-Méneville, 1835: 114 (in Boisduval, 1835).

Lycus (Metriorrhynchus) parallelus (Guérin-Méneville, 1835); Guérin-Méneville (1838: 72).

Metriorrhynchus parallelus (Guérin-Méneville, 1835); Waterhouse (1879: 47).

Cladophorus parallelus (Guérin-Méneville, 1835); Kleine (1933: 50).

Metriorrhynchus parvoniger Lea, 1921b, **comb. nov.** – Australia

Metriorrhynchus parvoniger (Lea, 1921b: 53; hereby returned to *Metriorrhynchus* Gemminger & Harold, 1869.

Porrostoma parvoniger (Lea, 1921b); Calder (1998: 158).

Metriorrhynchus paradoxa Blackburn, 1900, **comb. nov.** – Australia: Victoria

Metriorrhynchus paradoxa Blackburn, 1900: 55; hereby returned to *Metriorrhynchus* Gemminger & Harold, 1869.

Porrostoma paradoxa (Blackburn, 1900); Calder (1998: 160).

Metriorrhynchus pertenuis Lea, 1929, **comb. nov.** – Australia: Queensland

Metriorrhynchus pertenuis Lea, 1929: 336; hereby returned to *Metriorrhynchus* Gemminger & Harold, 1869.

Porrostoma pertenuis (Lea, 1929); Calder (1998: 160).

Metriorrhynchus philippinensis Waterhouse, 1879: 52 – Philippines

Metriorrhynchus pirlingi Kleine, 1926a: 114 – New Guinea

Metriorrhynchus platypus Kazantsev, 2015b: 222 – New Guinea

Metriorrhynchus posticalis Macleay, 1887, **comb. nov.** – Australia: Queensland

Metriorrhynchus posticalis Macleay, 1887: 234; hereby returned to *Metriorrhynchus* Gemminger & Harold, 1869.

Porrostoma posticalis (Macleay, 1887); Calder (1998: 160).

Metriorrhynchus puncticollis Waterhouse, 1879: 57 – New Guinea

Metriorrhynchus pusillus Kleine, 1928b, **comb. nov.** – Australia: Queensland

Metriorrhynchus pusillus Kleine, 1928b: 124; hereby returned to *Metriorrhynchus* Gemminger & Harold, 1869.

Porrostoma pusillus (Kleine, 1928b); Calder (1998: 160).

Metriorrhynchus putridus Kleine, 1935b: 181 – Solomon Isl.

Metriorrhynchus queenslandicus Kleine, 1928b, **comb. nov.** – Australia: Queensland

Metriorrhynchus queenslandicus Kleine, 1928b: 123; hereby returned to *Metriorrhynchus* Gemminger & Harold, 1869.

Porrostoma queenslandicus (Kleine, 1928b); Calder (1998: 160).

Metriorrhynchus quinquecavus Lea, 1921a, **comb. nov.** – Australia: Queensland

Metriorrhynchus quinquecavus Lea, 1921a: 192; hereby returned to *Metriorrhynchus* Gemminger & Harold, 1869.

Porrostoma quinquecavus (Lea, 1921a); Calder (1998: 160).

Metriorrhynchus ramicornis Lea, 1922, **comb. nov.** – Australia: Queensland

Metriorrhynchus ramicornis Lea, 1922: 2; hereby returned to *Metriorrhynchus* Gemminger & Harold, 1869.

Porrostoma ramicornis (Lea, 1922); Calder (1998: 160).

Metriorrhynchus reductus Pic, 1925 – New Guinea

Metriorrhynchus reductus Pic, 1925: 96.

=*Metriorrhynchus breveapicalis* Pic, 1923a: 39; Kleine (1933a: 82) (a junior primary homonym of *M. breveapicalis* Pic, 1923b: 3, hereby proposed).

Metriorrhynchus rigidus Kleine, 1943: 153 – New Guinea

Metriorrhynchus ruficollis Lea, 1921c, **comb. nov.** – Australia: Queensland

Metriorrhynchus ruficollis Lea, 1921c: 353; hereby returned to *Metriorrhynchus* Gemminger & Harold, 1869.

Porrostoma ruficollis (Lea, 1921c); Calder (1998: 161).

Metriorrhynchus rufirostris Lea, 1909, **comb. nov.** – Australia: Queensland

Metriorrhynchus rufirostris Lea, 1909: 88; hereby returned to *Metriorrhynchus* Gemminger & Harold, 1869.

Porrostoma rufirostris (Lea, 1909); Calder (1998: 162).

Metriorrhynchus rufomarginatus Lea, 1921b, **comb. nov.** – Australia: Victoria

Metriorrhynchus rufomarginatus Lea, 1921b: 58; hereby returned to *Metriorrhynchus* Gemminger & Harold, 1869.

Porrostoma rufomarginatus (Lea, 1921b); Calder (1998: 162).

Metriorrhynchus rusticus Waterhouse, 1879: 51 – New Guinea: Mysol

Metriorrhynchus sculpticollis Lea, 1921c, **comb. nov.** – Australia: Queensland

Metriorrhynchus sculpticollis Lea, 1921c: 352; hereby returned to *Metriorrhynchus* Gemminger & Harold, 1869.

Porrostoma sculpticollis (Lea, 1921c); Calder (1998: 162).

Metriorrhynchus semicyaneus Pic, 1923a: 38 – New Guinea: Yule Isl.

Metriorrhynchus semiflavus Lea, 1929, **comb. nov.** – Australia: Queensland

Metriorrhynchus semiflavus Lea, 1929: 340; hereby returned to *Metriorrhynchus* Gemminger & Harold, 1869.

Porrostoma sculpticollis (Lea, 1929); Calder (1998: 162).

Metriorrhynchus semiochraceus Pic, 1923b, **comb. nov.** – Australia

Metriorrhynchus semiochraceus Pic, 1923b: 3; hereby returned to *Metriorrhynchus* Gemminger & Harold, 1869.

Porrostoma semiochraceus (Pic, 1923b); Calder (1998: 162).

Metriorrhynchus sericans Waterhouse, 1879: 53 – Indo-Burma

Metriorrhynchus sericeicollis Schaufuss, 1887: 130 – Oceania: Vanuatu (Atchin Isl.)

Metriorrhynchus sericeus Waterhouse, 1879: 52 – Java

Note. *M. sericeus* is endemic to Java, other records are based on incorrect identification.

Metriorrhynchus serraticornis Macleay, 1887 – Australia: Queensland

Metriorrhynchus serraticornis Macleay, 1887: 230; hereby returned to *Metriorrhynchus* Gemminger & Harold, 1869.

Porrostoma serraticornis (Macleay, 1887); Calder (1998: 162).

Metriorrhynchus serricornis Macleay, 1887: 230 – New Guinea

Metriorrhynchus simsoni Lea, 1909 – Australia: Tasmania

Metriorrhynchus simsoni Lea, 1909: 82; hereby returned to *Metriorrhynchus* Gemminger & Harold, 1869.

Porrostoma simsoni (Lea, 1909); Calder (1998: 162).

Metriorrhynchus sinuaticollis Pic, 1923a, **comb. nov.** – Australia: New South Wales

Metriorrhynchus sinuaticollis Pic, 1923a: 35; Kleine, 1933a: 84; hereby returned to *Metriorrhynchus* Gemminger & Harold, 1869.

Porrostoma sinuaticollis (Pic, 1923a); Calder (1998: 163).

Metriorrhynchus smaragdinus Kleine, 1935c: 153 – New Guinea

Metriorrhynchus stenothorax Kazantsev, 2015b: 217 – New Guinea

Metriorrhynchus stenus Kazantsev, 2015b: 218 – New Guinea

Metriorrhynchus suaviter Kleine, 1926a: 107 – New Guinea

Metriorrhynchus takedai Bocak, Matsuda, Yagi, 2006: 124 – Philippines

Metriorrhynchus taoi Bocak & Matsuda, 1997: 412 – Sulawesi

Metriorrhynchus telnovi Kazantsev, 2015b: 223 – New Guinea

Metriorrhynchus tenebrans Kleine, 1926a: 98 – New Guinea

Metriorrhynchus tenebricosus Kleine, 1928b, **comb. nov.** – Australia: Queensland

Metriorrhynchus tenebricosus Kleine, 1928b: 125; hereby returned to *Metriorrhynchus* Gemminger & Harold, 1869.

Porrostoma tenebricosus (Kleine, 1928b); Calder (1998: 163).

Metriorrhynchus tenuis Macleay, 1886: 150 – New Guinea

Metriorrhynchus thoracicus (Fabricius, 1801) – Sulawesi, Moluccas,

Lycus thoracicus Fabricius, 1801: 117

Metriorrhynchus thoracicus (Fabricius, 1801); Gemminger & Harold (1869: 1630).

=*Metriorrhynchus cyanopterus* Redtenbacher, 1867: 412; Kleine (1933a: 84).

Note. No species found in collections from Aru Isl. and New Guinea which were reported earlier (Kleine, 1933)

Metriorrhynchus tibialis Lea, 1909, **comb. nov.** – Australia: Queensland

Metriorrhynchus tibialis Lea, 1909: 89; hereby returned to *Metriorrhynchus* Gemminger & Harold, 1869.

Porrostoma tibialis (Lea, 1909); Calder (1998: 163).

Metriorrhynchus tricavicollis Lea, 1921a, **comb. nov.** – Australia

Metriorrhynchus tricavicollis Lea, 1921a: 191; hereby returned to *Metriorrhynchus* Gemminger & Harold, 1869.

Porrostoma tricavicollis (Lea, 1921a); Calder (1998: 163).

Metriorrhynchus trichocerus Lea, 1921b, **comb. nov.** – Australia

Metriorrhynchus trichocerus Lea, 1921b: 61; hereby returned to *Metriorrhynchus* Gemminger & Harold, 1869.

Porrostoma trichocerus (Lea, 1921b); Calder (1998: 163).

Metriorrhynchus tricolor Kleine, 1935c: 153 – New Guinea

Metriorrhynchus turbinatus Kazantsev, 2015b: 218 – New Guinea

Metriorrhynchus tuzovi Kazantsev, 2015b: 214 – New Guinea

Metriorrhynchus unicolor Kleine, 1926a: 98 – New Guinea

Metriorrhynchus uniformis (Waterhouse, 1877), **comb. nov.** – Australia: Queensland

Porrostoma uniforme Waterhouse, 1877: 76.

Metriorrhynchus uniformis (Waterhouse, 1877); Kleine, 1933a: 84; hereby returned to *Metriorrhynchus* Gemminger & Harold, 1869.

Porrostoma uniforme (Waterhouse, 1877); Calder (1998: 163).

Metriorrhynchus variipennis Lea, 1909, **comb. nov.** – Australia: New South Wales

Metriorrhynchus variipennis Lea, 1909: 86; hereby returned to *Metriorrhynchus* Gemminger & Harold, 1869.

Porrostoma variipennis (Lea, 1909); Calder (1998: 163).

Metriorrhynchus versicolor Kleine, 1926a: 108 – New Guinea

Metriorrhynchus vittatus Blackburn, 1888, **comb. nov.** – Australia

Metriorrhynchus vittatus Blackburn, 1888: 258; hereby returned to *Metriorrhynchus* Gemminger & Harold, 1869.

Porrostoma vittatus (Blackburn, 1888); Calder (1998: 163).

Metriorrhynchus wallacei Kleine, 1933b: 13 – New Guinea: Mysol

Metriorrhynchus weigeli Kazantsev, 2015b: 215 – New Guinea

Metriorrhynchus yoshioi Bocak, Matsuda, Yagi, 2006: 125 – Sulawesi

Microtrichalus **Pic, 1921**

Microtrichalus Pic, 1921: 9 (Type species. *Microtrichalus singularis* Pic, 1921: 9; by monotypy).

=*Falsoenylus* Pic, 1926b: 29 (Type species. *Falsoenylus basipennis* Pic, 1926b: 30; by monotypy); Bocak, 1998a: 182.

Microtrichalus acutangulus (Waterhouse, 1878) – New Guinea

Trichalus acutangulus Waterhouse, 1878: 114.

Microtrichalus acutangulus (Waterhouse, 1878); Bocak (2000c: 12).

=*Trichalus blanchardi*, 1892: 423; Kleine (1933a: 69).

=*Trichalus imitator* Bourgeois, 1900: 424; Kleine (1933a: 69).

=*Trichalus incrassatus* 1900: 425; Kleine (1933a: 69).

Microtrichalus amoenus (Kleine, 1929) – Australia: Queensland

Trichalus amoenus Kleine, 1929: 479.

Microtrichalus amoenus (Kleine, 1929); Bocak (2000c: 12).

Microtrichalus bakeri (Kleine, 1929) – Philippines

Trichalus bakeri Kleine, 1929: 479.

Microtrichalus bakeri (Kleine, 1929); Bocak (1998c: 419).

Microtrichalus basipennis (Pic, 1926b) – Philippines

Falsoenylus basipennis Pic, 1926b: 30.

Microtrichalus basipennis Pic, 1926b: 31; Bocak (1998a: 182).

Microtrichalus basipes (Pic, 1923a) – New Guinea

Trichalus basipes Pic, 1923a: 36.

Microtrichalus basipes (Pic, 1923a); Bocak (2000c: 12).

=*Trichalus bicoloripes* Pic, 1930: 92; Bocak (2000c: 12).

=*Trichalus basicornis* Pic, 1930: 92; Bocak (2000c: 12).

Microtrichalus castigatus (Kleine, 1931: 287) – Java

Trichalus castigatus Kleine, 1931: 287.

Microtrichalus castigatus (Kleine, 1931); Bocak (2000c: 12).

Microtrichalus conditus (Kleine, 1927a) – Java

Trichalus conditus Kleine, 1927a: 312.

Microtrichalus conditus (Kleine, 1927a); Bocak (2000c: 12)

Microtrichalus costilis (Kleine, 1926b) – Philippines

Trichalus costilis Kleine, 1926b: 108.

Microtrichalus costilis (Kleine, 1926b); Bocak (1998c: 425).

Microtrichalus detractus (Waterhouse, 1879) – New Guinea, Moluccas: Key Isl.

Trichalus detractus Waterhouse, 1879: 70.

Microtrichalus detractus (Waterhouse, 1879); Bocak (2000c: 13).

Microtrichalus faustus (Kleine, 1935a) – New Guinea

Trichalus faustus Kleine, 1935a: 317.

Microtrichalus faustus (Kleine, 1935a); Bocak (2000c: 13).

Microtrichalus favorabilis (Kleine, 1926a: 177) – New Guinea – New Britain

Trichalus favorabilis Kleine, 1926a: 177.

Microtrichalus favorabilis (Kleine, 1926a); Bocak (2000c: 13).

Microtrichalus flavidus (Bourgeois, 1892: 41) – Borneo, Sumatra

Trichalus flavidus Bourgeois, 1892: 41.

Leptotrichalus flavidus (Bourgeois, 1892: 41); Kleine, 1930b: 335.

Microtrichalus flavidus (Bourgeois, 1892: 41); Bocak, 2000b: 183.

Microtrichalus fraterculus (Kleine, 1925b) – Moluccas: Buru

Trichalus fraterculus Kleine, 1925b: 36.

Microtrichalus fraterculus (Kleine, 1925b); Bocak (2000c: 13).

Microtrichalus fuliginosus (Bourgeois, 1883) – Borneo

Trichalus fuliginosus Bourgeois, 1883: 646.

Microtrichalus fuliginosus (Bourgeois, 1883); Bocak (2000c: 6).

=*Trichalus rianganus* Pic, 1927: 41; Bocak (2000c: 6).

Microtrichalus gorhami (Pic, 1926) – New Guinea

Trichalus gorhami Pic, 1926: 454.

Microtrichalus gorhami (Pic, 1926); hereby proposed.

Microtrichalus grandjeani (Pic, 1930) – New Guinea

Trichalus grandjeani Pic, 1930: 92.

Microtrichalus grandjeani (Pic, 1930); Bocak (2000c: 13).

Microtrichalus griseus (Waterhouse, 1879) – New Guinea

Trichalus griseus Waterhouse, 1879: 69.

Microtrichalus griseus (Waterhouse, 1879); Bocak (2000c: 13).

Microtrichalus halmaherensis Kazantsev 2010a: 95 – Moluccas: Halmahera

Microtrichalus ignotus (Kleine, 1936a) – Java

Trichalus ignotus Kleine, 1936: 133.

Microtrichalus ignotus (Kleine, 1936a); Bocak (2000c: 13).

Microtrichalus latesuturalis (Pic, 1926b: 30) – New Guinea

Trichalus latesuturalis Pic, 1926b: 30.

Microtrichalus latesuturalis (Pic, 1926b); Bocak (2000c: 13).

Microtrichalus leveri (Kleine, 1935b: 181) – Solomon Isl.

Leptotrichalus leveri Kleine, 1935b: 181.

Microtrichalus leveri (Kleine, 1935b); Bocak (2000c: 13).

Microtrichalus lineolatus (Kleine, 1939d) – Java

Trichalus lineolatus Kleine, 1939d: 132.

Microtrichalus lineolatus (Kleine, 1939d); Bocak (2000c: 14).

Microtrichalus milnei (Pic, 1923b) – New Guinea

Trichalus milnei Pic, 1923a: 36.

Microtrichalus milnei (Pic, 1923b); Bocak (2000c: 14).

Microtrichalus multicostatus (Pic, 1921) – Moluccas: Key Isl.

Trichalus multicostatus Pic, 1921: 10.

Microtrichalus multicostatus (Pic, 1921); Bocak (2000c: 14).

Microtrichalus pallescens (Pic, 1921) – Sulawesi

Trichalus pallescens Pic, 1921: 10.

Microtrichalus pallescens (Pic, 1921); Bocak (2000c: 14).

Microtrichalus patricius (Kleine, 1935b) – New Britain

Trichalus patricius Kleine, 1935b: 178.

Microtrichalus patricius (Kleine, 1935b); Bocak (2000c: 14).

Microtrichalus pectoralis (Pic, 1921) – New Guinea

Trichalus pectoralis Pic, 1921: 10.

Microtrichalus pectoralis (Pic, 1921); Bocak (2000c: 14).

Microtrichalus perturbatus (Waterhouse, 1879: 70) – New Guinea

Trichalus perturbatus Waterhouse, 1879: 70.

Microtrichalus perturbatus (Waterhouse, 1879); Bocak (2000c: 14).

Microtrichalus pomeraniensis (Pic, 1926b) – New Britain

Leptotrichalus pomeraniensis Pic, 1926b: 30.

Microtrichalus pomeraniensis (Pic, 1926b); Bocak, 2000c: 14.

Microtrichalus pubens (Kleine, 1935b) – Solomon Isl.

Trichalus pubens Kleine, 1935b: 179.

Microtrichalus pubens (Kleine, 1935b); Bocak (2000c: 14).

Microtrichalus pygoxanthus (Bourgeois, 1885) – Moluccas

Trichalus pygoxanthus Bourgeois, 1885: 84.

Microtrichalus pygoxanthus (Bourgeois, 1885); Bocak (2000c: 14).

Microtrichalus residuus (Kleine, 1939b) – Solomon Isl.

Trichalus residuus Kleine, 1939b: 590.

Microtrichalus residuus (Kleine, 1939b); Bocak (2000c: 15).

Microtrichalus retractus Bocak, 1998c: 423 – Philippines

Microtrichalus rouyeri (Pic, 1921) – Borneo

Trichalus rouyeri Pic, 1921: 11.

Microtrichalus rouyeri (Pic, 1921); Bocak (2000c: 8).

=*Trichalus diversus* Pic, 1927: 41; Bocak (2000c: 8).

=*Trichalus inconditus* Kleine, 1927a: 312; Bocak (2000c: 8).

=*Trichalus reductus* Pic, 1927: 42; Bocak (2000c: 8).

Microtrichalus rubricollis Bocak, 2000c: 10 – Sumatra

Microtrichalus salvani Bocak, 1998c: 423 – Philippines

Microtrichalus singularis (Pic, 1921: 9) – Java

Microtrichalus sulcaticeps (Pic, 1921) – Java

Trichalus sulcaticeps Pic, 1921: 10.

Microtrichalus sulcaticeps (Pic, 1921); Bocak (2000c: 15).

Microtrichalus suturalis Bocak & Bocakova 2000c: 51 – New Guinea

Microtrichalus tenimberensis (Kleine, 1933b) – Moluccas: Tanimbar

Trichalus tenimberensis Kleine, 1933b: 11.

Microtrichalus tenimberensis (Kleine, 1933b); Bocak (2000c: 15).

Microtrichalus testaceicollis (Pic, 1927) – Sumatra

Trichalus testaceicollis Pic, 1927: 42.

Microtrichalus testaceicollis (Pic, 1927); Bocak (2000c: 11).

Microtrichalus testaceicoxis (Pic, 1921) – Java

Trichalus testaceicoxis Pic, 1921: 10.

Microtrichalus testaceicoxis (Pic, 1921); Bocak (2000c: 15).

Microtrichalus villosipes Kazantsev, 2010: 95 – Moluccas: Halmahera

Mimoxylobanus Pic, 1921

Mimoxylobanus Pic, 1921: 11, hors texte (Type species. *Mimoxylobanus angustatus* Pic, 1921: 11 (by monotypy).

Mimoxylobanus angustatus Pic, 1921: 11 – Lesser Sundas: Sumbawa

Mimoxylobanus nubilus Kazantsev, 2015a: 115 – New Guinea

Porrostoma Laporte, 1838

Porrostoma Laporte, 1838: 26 (Type species. *Lycus rufipennis* Fabricius, 1801; by monotypy).

Porrostoma angustifasciatum Kazantsev, 2015b: 225 – New Guinea

Porrostoma apicale Waterhouse, 1877: 77 – Australia: Queensland

Porrostoma apicale Waterhouse, 1877: 77.

Metriorrhynchus apicalis Waterhouse, 1877: 77; Kleine (1933a: 78).

Porrostoma atriventris Pic, 1923a: 35 – Australia

Porrostoma atriventris Pic, 1923a: 35.

Metriorrhynchus atriventris Pic, 1923a: 35: Kleine (1933a: 78).

Porrostoma brevirostre Waterhouse, 1877: 74 – Australia: Victoria

Porrostoma brevirostre Waterhouse, 1877: 74; Calder (1998: 153).

Metriorrhynchus brevirostre Waterhouse, 1877); Kleine (1933a: 79).

Porrostoma echidna Kazantsev, 2015b: 225 – New Guinea

Porrostoma fallax Waterhouse, 1877: 79 – Australia: Queensland

Porrostoma fallax Waterhouse, 1877: 79; Calder (1998: 155).

Metriorrhynchus fallax (Waterhouse, 1877); Kleine (1933a: 80).

Porrostoma fasciapiculatum Kazantsev, 2015b: 231 – New Guinea

Porrostoma flava Kleine, 1926a: 99 – New Guinea

Porrostoma forticostatum (Pic, 1925a), **comb. nov.** – Australia

Cladophorus forticostatus Pic, 1925a: 8.

Porrostoma forticostatum (Pic, 1925a); hereby proposed.

Porrostoma fuscolineatus (Waterhouse, 1879) – Australia: Northwest Australia, New South Wales, Tasmania

Porrostoma fuscolineatus Waterhouse, 1879: 48.

Metriorrhynchus fuscolineatus (Waterhouse, 1879); Kleine (1933a: 80).

=*Porrostoma lineatum* Waterhouse, 1877: 78 a junior secondary homonym of *Metriorrhynchus lineatus* (Kirsch, 1875).

=*Metriorrhynchus laetus* Blackburn, 1891: 527; Kleine (1933a: 80).

Porrostoma gorhami Pic, 1923a: 35 – Australia

Porrostoma gorhami Pic, 1923a: 35; Calder (1998: 156).

Metriorrhynchus gorhami Pic, 1923a: 35; Kleine (1933a: 80).

Porrostoma halmaheraensis Kazantsev, 2015b: 230 – New Guinea

Porrostoma inflabellata Pic, 1923a: 35 – Australia

Porrostoma inflabellata Pic, 1923a: 35; Calder (1998: 157).

Metriorrhynchus inflabellatus (Pic, 1923a); Kleine (1933a: 81).

Porrostoma irregulare Waterhouse, 1877: 76 – Australia: Queensland

Porrostoma irregulare Waterhouse, 1877: 76; Calder (1998: 157).

Metriorrhynchus irregularis (Waterhouse, 1877); Kleine (1933a: 81).

Porrostoma laterale Redtenbacher, 1867: 100 – Australia: Queensland, New South Wales

Porrostoma laterale Redtenbacher, 1867: 100; Calder (1998: 157.

Metriorrhynchus lateralis Redtenbacher, 1867: 100; Kleine (1933a: 81

=*Metriorrhynchus marginicollis* Macleay, 1872: 263: Kleine (1933a: 81).

Porrostoma lucida (Kleine, 1926a); – New Guinea

Cladophorus lucidus Kleine, 1926aa: 132.

Porrostoma lucida (Kleine, 1926); Kazantsev (2015b: 225).

Porrostoma melaspis Bourgeois, 1889: 235 – Australia

Porrostoma melaspis Bourgeois, 1889: 236; Calder (1998: 158

Metriorrhynchus melaspis Bourgeois, 1889: 236; Kleine (1933a: 81).

Porrostoma rhipidium (Macleay, 1826) – Australia: except Northern Territory

Lycus rhipidium Macleay, 1826: 442.

Metriorrhynchus rhipidius Macleay, 1826: 442; Gemminger & Harold (1869: 1630).

Porrostoma rhipidium Macleay, 1826: 442; Calder (1998: 160).

=*Lycus septemcavus* Macleay, 1826: 442; Kleine (1933a: 82).

=*Porrostoma erythropterum* Erichson, 1842: 144; Kleine (1933a: 82).

=*Metriorrhynchus gigas* Blackburn, 1892: 524; Kleine (1933a: 82).

=*Metriorrhynchus flagellatus* Blackburn, 1900: 53; Kleine (1933a: 82).

Porrostoma rufipennis (Fabricius, 1801) – Australia, New Zealand

Lycus rufipennis Fabricius, 1801: 114.

Metriorrhynchus rufipennis (Fabricius, 1801); Kleine (1933a: 83).

Porrostoma rufipennis (Fabricius, 1801); Kuschel (1990: 53).

=*Porrostoma nigrirostris* Macleay in Dejean (1836: 111).

=*Porrostoma marginatus* Erichson, 1842: 145; Kleine (1933a: 83).

=*Lycus goryi* Le Guillou, 1844: 222; Kleine (1933a: 83).

=*Porrostoma haemorrhoidale* Waterhouse, 1877: 79; by Kleine (1933a: 83).

=*Porrostoma salebrosum* Waterhouse, 1877: 79; by Kleine (1933a: 83).

=*Porrostoma lugubre* Waterhouse, 1877: 80; Kleine (1933a: 83).

=*Porrostoma plagiatum* Waterhouse, 1877: 80; Kleine (1933a: 83).

=*Metriorrhynchus erraticus* Broun, 1893: 1146; Kuschel (1990: 53).

=*Metriorrhynchus miles* Blackburn, 1900: 54; Kleine (1933a: 83).

Porrostoma russatum Waterhouse, 1877: 77 – Australia: Queensland

Porrostoma russatum Waterhouse, 1877: 77; Calder (1998: 162.

Metriorrhynchus russatus (Waterhouse, 1877); Kleine (1933a: 83).

Porrostoma scalare Waterhouse, 1877: 79 – Australia: N. W. – Australia

Porrostoma scalare Waterhouse, 1877: 79; Calder (1998: 162).

Metriorrhynchus scalaris (Waterhouse, 1877); Kleine (1933a: 83).

Porrostoma textile Waterhouse, 1877: 77 – Australia: Queensland

Porrostoma textile Waterhouse, 1877: 77; Calder (1998: 163).

Metriorrhynchus textilis Waterhouse, 1877: 77; Kleine (1933a: 84).

Porrostoma tigroides Kazantsev, 2015b: 232 – New Guinea
Porrostoma togatum (Waterhouse, 1877) – Australia: Western – Australia

Metriorrhynchus togatus Waterhouse, 1877: 78; Kleine (1933a: 84).

Porrostoma togatum (Waterhouse, 1877): Calder (1998: 163).

Porrostoma viridum Kazantsev, 2015b: 227 – New Guinea
Porrostoma wilhelmi Kazantsev, 2015b: 228 – New Guinea

Oriomum Bocak, 1999b

Oriomum Bocak, 1999b: 111 (Type species. *Oriomum femoralis* Bocak, 1999b: 111, by original designation).

Oriomum femoralis Bocak, 1999b: 112 – New Guinea

Stadenus Waterhouse, 1879

Stadenus Waterhouse, 1879: 61 (Type species. *Porrostoma dichroum* Waterhouse, 1877: 86; by original designation).

Stadenus appositus Kleine, 1933b: 4 – Australia: Queensland
Stadenus atricornis (Lea, 1909) – Australia: Queensland

Stadenus atricornis Lea, 1909: 85.

Metriorrhynchus atricornis Lea, 1909: 85; Kleine (1922: 5).

Stadenus dichroum (Waterhouse, 1877) – Australia: West Australia

Porrostoma dichroum Waterhouse, 1877: 86.

Stadenus dichrous Waterhouse, 1877; Kleine (1933a: 43).

Metriorrhynchus dichrous Waterhouse, 1877: 86; Lea (1909: 67).

Stadenus inquilinus (Waterhouse, 1877) – Australia

Porrostoma inquilinum Waterhouse, 1877: 81.

Stadenus inquilinus Waterhouse, 1877: 81; Kleine (1933a: 43).

Metriorrhynchus inquilinus Waterhouse, 1877: 81 (Lea, 1909: 67).

Stadenus nigrovittatus (Lea, 1909) – Australia: New South Wales

Metriorrhynchus nigrovittatus Lea, 1909: 78.

Stadenus nigrovittatus Lea, 1909: 78; Kleine (1933a: 43).

Stadenus obscuripennis (Lea, 1909) – Australia: Tasmania

Metriorrhynchus obscuripennis Lea, 1909: 77.

Stadenus obscuripennis Lea, 1909: 77; Kleine (1933a: 43).

Stadenus puncticollis Kleine, 1933b: 3 – Australia: West Australia
Stadenus triareolatus (Lea, 1909) – Australia: West – Australia

Metriorrhynchus triareolatus Lea, 1909: 78.

Stadenus triareolatus Lea, 1909: 78; Kleine (1933a: 43).

Metriorrhynchoides Kleine, 1926a

Metriorrhynchoides Kleine, 1926a: 118 (Type species. *Metriorrhynchoides helleri* Kleine, 1926a: 119; by original designation).

Metriorrhynchoides eminens Kleine, 1926a: 120 – New Guinea
Metriorrhynchoides flavofasciatus Kleine, 1926a: 120 – New Guinea
Metriorrhynchoides helleri Kleine, 1926a: 119 – New Guinea
Metriorrhynchoides pulcher Kleine, 1926a: 119 – New Guinea

***Procautires* Kleine, 1925** b

Procautires Kleine, 1925b: 32 (Type species. *Procautires toxopei* Kleine, 1925b: 32; by original designation).

Procautires aflabellatus Kazantsev, 2016b: 44 – New Guinea
Procautires angustiformis Kazantsev, 2016b: 35 – New Guinea
Procautires angustisuturalis Kazantsev, 2016b: 36 – New Guinea
Procautires bicoloripectus Kazantsev, 2016b: 38 – New Guinea
Procautires cladophoroides Kazantsev, 2016b: 38 – New Guinea
Procautires bryanti Kleine, 1933b: 10 – Australia: New South Wales
Procautires cygnus Kazantsev, 2016b: 42 – New Guinea
Procautires divisus Kleine, 1935a: 320 – New Guinea
Procautires efferatus Kleine, 1926a: 158 – New Guinea
Procautires excitatus Kleine, 1926a: 160 – New Guinea
Procautires flavicolor Kleine, 1926a: 157 – New Guinea
Procautires forficefer Kazantsev, 2016b: 39 – New Guinea
Procautires honestus Kleine, 1926a: 157 – New Guinea
Procautires maculosus Kleine, 1926a: 159 – New Guinea
Procautires maklaii Kazantsev, 2016b: 45 – New Guinea
Procautires miklukhai Kazantsev, 2016b: 46 – New Guinea
Procautires miniatus Kleine, 1926a: 158 – New Guinea
Procautires misoolensis Kazantsev, 2016b: 40 – New Guinea
Procautires mixtus (Waterhouse, 1879) – New Guinea
Xylobanus mixtus Waterhouse, 1879: 43.
Procautires mixtus Waterhouse, 1879: 43, Kleine (1933a: 59).
=*Xylobanus acheronticus* Bourgeois, 1892: 504.
=*Procautires acheronticus* (Bourgeois, 1892); Kleine (1933a: 59).
Procautires multipilosus Kazantsev, 2016b: 41 – New Guinea
Procautires nabirensis Kazantsev, 2016b: 41 – New Guinea
Procautires nigropumilio Kazantsev, 2016b: 45 – New Guinea
Procautires parallelus Bourgeois, 1892: 505 – New Guinea
Cladophorus parallelus Bourgeois, 1892: 505.
Procautires parallelus Bourgeois, 1892: 505, Kleine (1933a: 59).
Procautires sibelaensis Kazantsev, 2016b: 41 – New Guinea
Procautires subparallelus Bocak et al. 2020 – New Guinea

Xylobanus parallelus Pic, 1922: 16.

Procautires parallelus Pic, 1922: 16; Pic, 1926a: 454 [a junior secondary homonym of *Procautires parallelus* (Bourgeois, 1883)].

Procautires subparallelus nom. nov.; hereby designated.

Procautires suturalis Kleine, 1926a: 159 – New Guinea

Procautires toxopei Kleine, 1925b: 32 – Moluccas: Buru

Procautires wauvagus Kazantsev, 2016b: 43 – New Guinea

***Pseudodontocerus* Pic, 1921**

Pseudodontocerus Pic, 1921: 12 (Type species. *Pseudodontocerus pulcher* Pic, 1921: 12; by monotypy).

=*Carathrix* Kleine, 1926a: 149 (Type species. *Carathrix pilosus* Kleine, 1926a: 149; by original designation), Bocak (2002: 344).

Pseudodontocerus bicoloripes Pic, 1923a: 38 – New Guinea: Roon Isl., – Moluccas: Bacan

Pseudodontocerus pilosus (Kleine, 1926a) – New Guinea

Carathrix pilosus Kleine, 1926a: 149.

Pseudodontocerus pilosus (Kleine, 1926a: 149); Bocak (2002: 344).

Pseudodontocerus pulcher Pic, 1921: 12 – New Guinea

Pseudodontocerus ruficollis (Guérin-Méneville, 1830), **comb. nov.** – New Guinea

Cladophorus ruficollis Guérin-Méneville, 1830: plate 2, fig. 8.

Pseudodontocerus ruficollis (Guérin-Méneville, 1830); hereby designated.

=*Cladophorus crocicollis* Boisduval, 1835: 116; Gemminger & Harold, 1869: 1633.

***Schizotrichalus* Kleine, 1926**

Schizotrichalus Kleine, 1926a: 183. (Type species. *Trichalus nigrescens* Waterhouse, 1879: 70; by original designation).

S. nigrescens (Waterhouse, 1879: 70 – New Guinea: Raja Ampat

Trichalus nigrescens (Waterhouse, 1879: 70.

Schizotrichalus nigrescens (Waterhouse, 1879); Kleine (1926a: 183).

S. halmaherensis Bocek & Adamkova, 2019: 344 – Moluccas: Halmahera

***Sulabanus* Dvorak & Bocak, 2007.**

Sulabanus Dvorak & Bocak, 2007: 3 (Type species. *S. mamasensis* Dvorak & Bocak, 2007: 7; by original designation).

Sulabanus ambangensis Dvorak & Bocak, 2007: 15 – Sulawesi

Sulabanus amporiwensis Dvorak & Bocak, 2007: 17 – Sulawesi
Sulabanus barclayi Dvorak & Bocak, 2007: 10 – Sulawesi
Sulabanus brancuccii Dvorak & Bocak, 2007: 18 – Sulawesi
Sulabanus brunneus Dvorak & Bocak, 2007: 12 – Sulawesi
Sulabanus cordatus Dvorak & Bocak, 2007: 9 – Sulawesi
Sulabanus dumongabonensis Dvorak & Bocak, 2007: 13 – Sulawesi
Sulabanus gracilis Dvorak & Bocak, 2007: 15 – Sulawesi
Sulabanus katarinae Dvorak & Bocak, 2007: 14 – Sulawesi
Sulabanus lalui Dvorak & Bocak, 2007: 11 – Sulawesi
Sulabanus lineatus Dvorak & Bocak, 2007: 14 – Sulawesi
Sulabanus mamasensis Dvorak & Bocak, 2007: 7 – Sulawesi
Sulabanus major Dvorak & Bocak, 2007: 16 – Sulawesi
Sulabanus minor Dvorak & Bocak, 2007: 9 – Sulawesi
Sulabanus nigricolor (Pic, 1922) – Sulawesi

Xylobanus nigricolor Pic, 1922: 4.

Sulabanus nigricolor (Pic, 1922); Dvorak & Bocak (2007: 21).

Sulabanus nigricordatus Dvorak & Bocak, 2007: 10 – Sulawesi
Sulabanus niger Dvorak & Bocak, 2007: 16 – Sulawesi
Sulabanus ocularis Dvorak & Bocak, 2007: 18 – Sulawesi
Sulabanus pendolensis Dvorak & Bocak, 2007: 11 – Sulawesi
Sulabanus robustus Dvorak & Bocak, 2007: 17 – Sulawesi
Sulabanus rufomarginatus Dvorak & Bocak, 2007: 12 – Sulawesi
Sulabanus similis Dvorak & Bocak, 2007: 19 – Sulawesi
Sulabanus ulci Dvorak & Bocak, 2007: 7 – Sulawesi

***Synchonnus* Waterhouse, 1879**

Synchonnus Waterhouse, 1879: 59, *S. clientulus* Waterhouse, 1879: 59; by monotypy).

=*Achras* Waterhouse, 1879: 61 (Type species. *A. limbatus* Waterhouse, 1879: 62; by monotypy); Kusy et al. (2018: 29).

=*Enylus* Waterhouse, 1879: 72 (Type species. *E. segregatus* Waterhouse, 1879; by monotypy); Kusy et al. (2018: 29).

=*Strophicus* Waterhouse, 1879: 73 (Type species. *S. nigellus* Waterhouse, 1879: 73; by monotypy); Bocak (2002: 335).

Synchonnus ailaketoae Kusy et al., 2018: 32 – Australia: Queensland
Synchonnus amplus (Kleine, 1930) – Australia: New South Wales

Enylus amplus Kleine, 1930b: 329.

Synchonnus amplus (Kleine, 1930b); Kusy et al. (2018: 29).

Synchonnus campestris Kusy et al., 2018: 35 – Australia: Queensland
Synchonnus chilvertonensis Kusy et al., 2018: 33 – Australia: Queensland
Synchonnus clientulus (Waterhouse, 1877) – Australia: Queensland

Porrostoma clientulum Waterhouse, 1877: 81.

Synchonnus clientulus (Waterhouse, 1877); Waterhouse (1879: 59).

Synchonnus corrugatus Pic, 1923a: 35 – Australia

Synchonnus crypticum Kusy et al., 2018: 36 – Australia: Queensland

Synchonnus dubenovae Kusy et al., 2018: 32 – Australia: Queensland

Synchonnus etheringtoni Kusy, 2017: 156 – New Guinea

Synchonnus eungellensis Kusy et al., 2018: 34 – Australia: Queensland

Synchonnus flavonotatum Kusy et al., 2018: 33 – Australia: Queensland

Synchonnus humeralis Pic, 1923a: 37 – Moluccas: Obi Isl.

Synchonnus imperfectus (Kleine, 1935a), **comb. nov.** – New Guinea

Strophicus imperfectus Kleine, 1935a: 320.

Synchonnus imperfectus (Kleine, 1935a); hereby proposed.

Synchonnus leucomelas (Kleine, 1926a), **comb. nov.** – New Guinea

Strophicus leucomelas Kleine, 1926a: 152.

Synchonnus leucomelas Kleine, 1926a: 152; hereby proposed.

Synchonnus limbatum (Waterhouse, 1877) – Australia: New South Wales

Porrostoma limbatum Waterhouse, 1877: 81.

Achras limbatum Waterhouse, 1877: 81; Waterhouse (1879: 61).

Synchonnus limbatum (Waterhouse, 1877); Kusy et al. (2018: 29).

Synchonnus maseki Kusy et al., 2018: 32 – Australia: Queensland

Synchonnus monteithi Kusy et al., 2018: 35 – Australia: Queensland

Synchonnus nigellus (Waterhouse, 1879) – New Guinea: Mysol

Strophicus nigellus Waterhouse, 1879: 73.

Synchonnus nigellus (Waterhouse, 1879: 73); Bocak (2002: 335).

Synchonnus segregatus (Waterhouse, 1879) – Australia: Queensland

Enylus segregatus Waterhouse, 1879: 72.

Synchonnus segregatus (Waterhouse, 1879); Kusy et al. (2018: 29).

Synchonnus slipinskii Kusy et al., 2018: 34 – Australia: Queensland

Synchonnus testaceithorax Pic, 1923a: 35 – New Guinea

Synchonnus variabilis Kusy et al., 2018: 36 – Australia: Queensland

Trichalus **Waterhouse, 1877**

Trichalus Waterhouse, 1877: 82 (Type species. *Trichalus flavopictus* Waterhouse, 1877: 82; by subsequent designation).

=*Xantheros* Fairmaire 1877: 167 (Type species. *Xantheros ochreatus* Fairmaire 1877: 167; by subsequent designation).

Trichalus aemulus Waterhouse, 1878: 114 – Moluccas: Bacan

Trichalus albertisi Bourgeois, 1900: 428 – New Guinea

Trichalus amicus Kleine, 1929: 482 – Australia: Queensland

Trichalus ampliatus Waterhouse, 1877 – Australia: New South Wales, Victoria

Trichalus ampliatus Waterhouse, 1877: 83.

=*Trichalus auritus* Lea, 1895: 600; Kleine (1933a: 69).

=*Trichalus distinctus* Lea, 1895: 602; Kleine (1933a: 69).

Trichalus anceps Waterhouse, 1878 – Moluccas: Bacan Ternate

Trichalus anceps Waterhouse, 1878: 114.

=*Trichalus ternatensis* Bourgeois, 1900: 427 (a senior secondary homonym of *Trichalus ternatensis* Kleine, 1930); Kleine (1933a: 69).

Trichalus angularis Kleine, 1929: 482 – Australia: Queensland

Trichalus angulicollis (Fairmaire 1877) – Australia: Queensland

Xantheros angulicollis Fairmaire 1877: 167.

Trichalus angulicollis Fairmaire 1877: 167; Lea (1909: 94).

Trichalus angustulus McLeay 1887: 235 – Australia: Queensland

Trichalus angustus Kleine, 1929: 480 – Australia: Queensland

Trichalus animosus Kleine, 1929: 477 – Australia

Trichalus antiquus Kleine, 1939c: 12 – Java

Trichalus apicalis McLeay 1886: 152 – New Guinea

Trichalus apiciflavus Lea, 1909: 99 – Australia: New South Wales

Trichalus apparatus Kleine, 1929: 481 – Australia: Queensland

Trichalus applicatus Kleine, 1929: 484 – Australia: Queensland

Trichalus argutus Kleine, 1929: 478 – Australia: Queensland

Trichalus ater McLeay 1887: 233 – Australia: Queensland

Trichalus atripennis McLeay 1887: 234 – Australia: New South Wales Queensland

Trichalus basipennis Lea, 1922: 6 – Australia: Queensland

Trichalus bifurcatus Lea, 1909: 96 – Australia: Tasmania

Trichalus communis Waterhouse, 1879 – Sundas, Malaya

Trichalus communis Waterhouse, 1879: 71; Bocek et al. (2017: 7).

Microtrichalus communis (Waterhouse, 1879); Bocak (1998c: 426).

=*Trichalus barbieri* Pic, 1949: 14; Bocak (2000c: 5).

=*Trichalus javanus* Pic, 1927: 42; Bocak (2000c: 5).

=*Trichalus laticornis* Pic, 1927: 42; Bocak (2000c: 5).

=*Trichalus nothus* Kleine, 1933b: 12; Bocak (2000c: 5).

=*Trichalus undulatithoraxis* Pic, 1927: 42; Bocak (2000c: 5).

Trichalus curticollis Pic, 1927: 42; Bocak, 2000c: 5 – Java

Trichalus discoideus (Erichson 1842) – Australia: Tasmania

Porrostoma discoideus Erichson 1842: 145.

Metriorrhynchus discoideus (Erichson 1842); Gemminger & Harold (1869: 1629).

Trichalus discoideus (Erichson 1842); Kleine (1933a: 71).

Trichalus discretus Bourgeois, 1884: 67 – Java

Trichalus faustus Kleine, 1935a: 317 – New Guinea

Trichalus flavicans Blanchard 1856: 76 – New: Guinea: Aru Isl.

Trichalus flavopictus Waterhouse, 1877: 82 – Australia: Queensland

Trichalus foveolatus Pic, 1921: 10 – Moluccas: Obi

Trichalus froggatti (Macleay 1887: 233) – Australia: Queensland

Xylobanus froggatti McLeay 1887: 233.

Trichalus froggatti (Macleay 1887); Kleine (1933a: 71).

Trichalus gorhami Pic, 1926a: 454 – New Guinea
Trichalus griffithi Lea, 1909: 98 – Australia: Queensland
Trichalus hirsutus Kleine, 1926a: 173 – New Guinea
Trichalus honoratus Kleine, 1929: 484 – Wetar Isl.
Trichalus immanus Kleine, 1929: 478 – New Guinea
Trichalus infaustus Lea, 1921b: 62 – Australia
Trichalus insignis Lea, 1895: 601 – Australia: New South Wales
Trichalus keiensis Pic, 1921: 10 – Moluccas: Key Isl.
Trichalus pseudoternatensis **nom. nov.** – Moluccas: Ternate

Diatrichalus ternatensis Kleine, 1930b: 329.

Trichalus ternatensis (Kleine, 1930b), a junior secondary homonym of *Trichalus ternatensis* Bourgeois, 1900; Bocak (2001a: 30).

Trichalus pseudoternatensis **nom. nov.**

Trichalus lanatus Kleine, 1926a: 172 – New Guinea
Trichalus languidus Bourgeois, 1900: 423 – New Guinea
Trichalus lateapicalis Pic, 1930: 92 – Moluccas: Bacan
Trichalus leopoldi Pic, 1932: 87 – New Guinea: Aru Isl.
Trichalus lineatus Pic, 1931: 26 – Malaya
Trichalus manifestus Kleine, 1926a: 177 – New Guinea
Trichalus marcidus Kleine, 1926a: 175 – New Guinea
Trichalus metasternalis Lea, 1929: 340 – Australia: Northern Territory
Trichalus nigroscutellatus Kleine, 1926: 176 – New Guinea
Trichalus nubicollis (Fairmaire, 1877) – Australia: Queensland

Xantheros nubicollis Fairmaire, 1877: 167.

Trichalus nubicollis (Fairmaire, 1877); Fairmaire (1879: 99).

Trichalus ochreatus (Fairmaire 1877) – Australia: New South Wales

Xantheros ochreatus Fairmaire, 1877: 167.

Trichalus ochreatus (Fairmaire, 1877); Fairmaire (1879: 99).

Trichalus pallidipennis McLeay 1886: 152 – New Guinea
Trichalus patricius Kleine, 1935b: 178 – New Britain
Trichalus perplexus Bourgeois, 1900: 430 – Moluccas: Ternate
Trichalus placidus Lea, 1922: 5 – Australia: Queensland
Trichalus praecipuus Kleine, 1933b: 12 – Moluccas: Bacan
Trichalus pubens Kleine, 1935b: 179 – New Britain
Trichalus pulcherrimus Kleine, 1926a: 174 – New Guinea
Trichalus quadricavus Lea, 1921b: 62 – Australia: Queensland
Trichalus repandus Kleine, 1929: 479 – Moluccas
Trichalus residuus Kleine, 1939b: 590 – Solomon Isl.
Trichalus secretus Kleine, 1935a: 317 – New Guinea
Trichalus serraticornis (Fabricius, 1775) – Australia

*Pyrochroa serr*aticornis Fabricius 1775: 203.

Trichalus serraticornis (Fabricius 1775); Waterhouse (1877: 84).

Trichalus signatus Kleine, 1939c: 19 – Moluccas: Buru

Trichalus subarcuatithorax Pic, 1926a: 454 – New Guinea
Trichalus subtilis Kleine, 1933b: 12 – Moluccas: Bacan
Trichalus sulcatiformis Kleine, 1934: 116 – Australia: Northern Territory
Trichalus sulcatus Waterhouse, 1877: 83 – Australia: Queensland
Trichalus umbrosus Kleine, 1929: 485 – New Guinea
Trichalus versicolor Kleine, 1929: 486 – Moluccas: Bacan

Wakarumbia Bocak, 1999a

Wakarumbia Bocak, 1999a: 166 (Type species. *W. gracilis* Bocak, 1999a: 167; by original designation).

Wakarumbia amporivensis Bocak, 2001b: 261– Sulawesi
Wakarumbia angustior (Pic, 1922) – Sulawesi
Xylobanus angustior Pic, 1922: 17.
Wakarumbia angustior (Pic, 1922); Bocak & Bocakova (2000d: 44).
Wakarumbia aurea Dvorak & Bocak, 2009: 53 – Sulawesi
Wakarumbia brendelli Bocak, 2000d: 273 – Sulawesi
Wakarumbia brunnescens Bocak, 2000d: 274 – Sulawesi
Wakarumbia celebensis (Kleine, 1933) – Sulawesi
Protaphes celebensis Kleine, 1933b: 2.
Wakarumbia celebensis Kleine, 1933b: 2: Bocak (2000d: 274).
Wakarumbia decepta Dvorak & Bocak, 2009: 53 – Sulawesi
Wakarumbia equalis Bocak, 2001b: 262 – Sulawesi
Wakarumbia fasciata Bocak, 2001b: 263 – Sulawesi
Wakarumbia flavohumeralis Bocak, 2000d: 275 – Sulawesi
Wakarumbia gracilis Bocak, 1999a: 167 – Sulawesi: Buton Isl.
Wakarumbia grandis Bocak, 2000d: 276 – Sulawesi
Wakarumbia grisea Bocak, 2001b: 264 – Sulawesi
Wakarumbia hanae Dvorak & Bocak, 2009: 54 – Sulawesi
Wakarumbia hirsuta Dvorak & Bocak, 2009: 54 – Sulawesi
Wakarumbia kalamensis Bocak, 2001b: 264 – Sulawesi
Wakarumbia kundratai Dvorak & Bocak, 2009: 56 – Sulawesi
Wakarumbia lateniger (Pic, 1922) – Sulawesi
Xylobanus angustior lateniger Pic, 1922: 16.
Wakarumbia lateniger (Pic, 1922); Bocak & Bocakova (2000: 44).
Wakarumbia linearis Dvorak & Bocak, 2009: 56 – Sulawesi
Wakarumbia mamasensis Bocak, 2001b: 265 – Sulawesi
Wakarumbia montana Bocak, 2001b: 266 – Sulawesi
Wakarumbia nigra Bocak, 2000d: 276 – Sulawesi
Wakarumbia obscura Dvorak & Bocak, 2009: 57 – Sulawesi
Wakarumbia orobuensis Bocak, 2001b: 266 – Sulawesi
Wakarumbia obstinata Dvorak & Bocak, 2009: 58 – Sulawesi
Wakarumbia pendolensis Dvorak & Bocak, 2009: 58 – Sulawesi

Wakarumbia petri Bocak, 2001b: 266 – Sulawesi
Wakarumbia pseudofasciata Dvorak & Bocak, 2009: 59 – Sulawesi
Wakarumbia oculata Bocak, 2000d: 276 – Sulawesi
Wakarumbia pallescens Bocak, 2000d: 276 – Sulawesi
Wakarumbia similis Bocak, 2000d: 277 – Sulawesi

Xylobanomimus Kleine, 1926

Xylobanomimus Kleine, 1926a: 166 (Type species. *Xylobanomimus papuensis* Kleine, 1926a: 166; by original designation).

Xylobanomimus papuensis Kleine, 1926a: 166 – New Guinea

Xylobanomorphus Kleine, 1935a

Xylobanomorphus Kleine, 1935a: 316 (Type species. *Xylobanomorphus transformis* Kleine, 1935a: 316; by monotypy).

Xylobanomorphus balkei Kazantsev, 2010: 97 – New Guinea
Xylobanomorphus elongatissimus Kazantsev, 2010: 97 – New Guinea
Xylobanomorphus transformis Kleine, 1935a: 316. – New Guinea
Xylobanomorphus triton Kazantsev, 2015a: 113 – New Guinea
Dubious placement:
Xylobanomorphus villosus (Kazantsev, 2006) – Sri Lanka

Xylobanus villosus Kazantsev, 2006: 99.

Xylobanomorphus villosus (Kazantsev, 2006); Kazantsev 2010: 99.

Note. The placement of this species needs further revision, see the comments in Results and Discussion above.

Xylothrix Kazantsev, 2015a

Xylothrix Kazantsev, 2015a: 115 (Type species. *Xylothrix athrix* Kazantsev, 2015a: 115; by original designation).

Xylothrix athrix Kazantsev, 2015a: 115 – New Guinea
Xylothrix avicollis Kazantsev, 2015a: 116 – New Guinea

Incertae sedis in Metriorrhynchina

(these species have been placed in *Xylobanus* Waterhouse, 1879 if not stated otherwise)

(Xylobanus) ampliatus Macleay, 1887: 233 – Australia: Queensland
(Xylobanus) australianus Kleine, 1927c: 70 – Australia: New South Wales
(Xylobanus) baitetaensis Kazantsev, 2015a: 108 – New Guinea

(Xylobanus) pseudobasalis Bocak et al., 2020, **nom. nov.** – Australia: Queensland

Metriorrhynchus basalis Lea, 1921a: 190 (a junior primary homonym of *Metriorrhynchus basalis* Bourgeois, 1911).

Porrostoma basalis (Lea, 1921); Calder (1998: 153).

Porrostoma pseudobasalis Bocak et al., 2020; a new replacement name for *Metriorrhynchus basalis* Lea, 1921.

(Xylobanus) pseudobasalis (Bocak et al., 2020); hereby proposed.

(Xylobanus) basiflavus Lea, 1909: 76 – Australia: Queensland

(Xylobanus) cancellatus Lea, 1909: 73 – Australia: Queensland, New South Wales

(Xylobanus) canus Kleine, 1927c: 54 – Australia

(Xylobanus) confluens Bourgeois, 1900: 420 – New Guinea

Cautires confluens Bourgeois, 1900: 420.

Xylobanus confluens Bourgeois, 1900: 420;165 Kleine (1926: 165).

(Xylobanus) conquisitus Kleine, 1927c: 46 – Australia: Tasmania

(Xylobanus) coenosus Lea, 1899: 557 – Australia: New South Wales

(Xylobanus) constricticollis Lea, 1909: 76 – Australia Western – Australia

(Xylobanus) corvus Kleine, 1935a: 315 – New Guinea

(Xylobanus) culex Kazantsev, 2015a: 111 – New Guinea

(Xylobanus) densereticulatus Kleine, 1927c: 47 – New Guinea

(Xylobanus) diminutivus Lea, 1909: 84 – Australia: Queensland

(Xylobanus) fakfakensis Kazantsev, 2015a: 112 – New Guinea

(Xylobanus) flavomarginatus Kleine, 1933b: 11 – Australia

(Xylobanus) fumosus Macleay, 1887: 231 – Australia: Queensland

(Xylobanus) hackeri Kleine, 1933b: 11 – Australia: Queensland

(Xylobanus) heterodoxus Lea, 1909: 87 – Australia: New South Wales

(Xylobanus) insignipennis Blackburne, 1900: 52 – Australia: Tasmania

(Xylobanus) longicornis Macleay, 1887: 232 – Australia: Queensland

(Xylobanus) meyricki Blackburne, 1886: 258 – Australia: Western – Australia

(Xylobanus) milnei Pic, 1923a: 36 – New Guinea

(Xylobanus) mimicus Lea, 1921, **comb. nov.** – Australia: Victoria

Metriorrhynchus mimicus Lea, 1921b: 54.

Porrostoma mimicus (Lea, 1921); Calder (1998: 158).

(Xylobanus) mimicus Lea, 1921, hereby proposed.

(Xylobanus) miniaticollis Macleay, 1887: 232 – Australia: Queensland

(Xylobanus) mirabilis Kleine, 1926a: 165 – New Guinea

(Xylobanus) mobilis Kleine, 1926a: 164 – New Guinea

(Xylobanus) modestus Kleine, 1926a: 163 – New Guinea

(Xylobanus) neglectus Kleine, 1926a: 164 – New Guinea

(Xylobanus) nigronotatus Pic, 1923a: 36 – New Guinea

(Xylobanus) obscurus Macleay, 1886: 151 – New Guinea

(Xylobanus) parvulus Pic, 1922: 14 – New Guinea

(Xylobanus) pecten Kazantsev, 2015a: 109 – New Guinea

(Xylobanus) piceoscutus Pic, 1922: 14 – New Guinea

(Xylobanus) ramosus Lea, 1909: 74 – Australia: New South Wales

(Xylobanus) robustithorax Kleine, 1927c: 71 – Moluccas: Halmahera
(Xylobanus) rotundeareolatus Kazantsev, 2015a: 109 – New Guinea
(Xylobanus) simplicicornis Lea, 1909: 74 – Australia: Queensland
(Xylobanus) sodalis Kleine, 1935a: 315 – New Guinea
(Xylobanus) tamborinensis Calder, 1998, **comb. nov.** – Australia: Queensland

Porrostoma tamborinensis Calder (1998: 163 (a replacement name for *Metriorrhynchus marginicollis* Lea, 1921).

=*Metriorrhynchus marginicollis* Lea, 1921b: 55 (a junior primary homonym of *Metriorrhynchus marginicollis* Macleay, 1872).

=*Porrostoma marginicollis* (Lea, 1921); Calder (1998: 163).

(Xylobanus) tamborinensis (Calder, 1998), hereby proposed.

(Xylobanus) telefominensis Kazantsev, 2015a: 110 – New Guinea
(Xylobanus) testaceicollis Macleay, 1887: 234 – Australia: Queensland
(Xylobanus) testaceoapicalis Pic, 1923c: 2 – Australia
(Xylobanus) testaceohumeralis Pic, 1923c: 2 – Australia
(Xylobanus) testaceoscutus Pic, 1922: 15 – New Guinea
(Xylobanus) uniseriatus Lea, 1909: 72 – Australia: New South Wales
(Xylobanus) venustus Kleine, 1935a: 316 – New Guinea
(Xylobanus) versicolor Kleine, 1927c: 70 – Australia: South – Australia
(Xylobanus) waigeoensis Kazantsev, 2015a: 110 – New Guinea

References

Blackburn, T. **1888** Further notes on Australian Coleoptera, with descriptions of new species. *Trans. R. Soc. S. Austral.* 10, 177–287.

Blackburn, T. **1892** Notes on Australian Coleoptera, with descriptions of new species. Part X. *Proc. Linn. Soc. N. S. W.* (2)6, 479–550.

Blackburn, T. **1900** Further notes on Australian Coleoptera, with descriptions of new genera and species. Part XXVI. *Trans. R. Soc. S. Austral.* 24, 35–68.

Blanchard, E. **1856** *Voyage au pole sud et dans l'Océanie sur les corvettes l'Astrolabe et la Zélée: pendant les années 1837–1840 by Dumont d'Urville, Astrolabe expedition, 1837–1840.* Vol. iv., Zoologie Insectes. Didot: Paris, 422 pp.

Bocak, L. **1998a** A generic revision and phylogenetic analysis of the subtribe Trichalinina (Coleoptera: Lycidae: Metriorrhynchini). *Acta Soc. Zool. Boh.*, 62, 167–200.

Bocak, L. **1998b**.Nomenclatural notes on taxa of the family Lycidae described by Guérin-Méneville (Insecta: Coleoptera). *Ann. Zool.*, 48, 245–251.

Bocak, L. **1998c** A revision of the genus *Microtrichalus* Wat. from the Philippines (Coleoptera: Lycidae). *Eur. J. Entomol.*, 95, 417–428.

Bocak, L. **1998d** A new species of the genus *Eniclases* Wat. (Coleoptera, Lycidae). *Acta Univ. Palack. Olom.* 37, 35(1997), 13–16.

Bocak, L. **1999a** New taxa from the subtribe Hemiconderinina. *Entomologische Blaetter* 95, 166–170.

Bocak, L. **1999b** A new genus *Oriomum* from New Guinea (Coleoptera, Lycidae, Metriorrhynchini). *Entomologia Basilensia* (1998), 21, 111–114.

Bocak, L. **2000a** A revision of the genus *Diatrichalus* Kleine from the Philippines (Coleoptera: Lycidae). *Raffl. Bull. Zool.* 48, 11–16.

Bocak, L. **2000b** To the knowledge of the genus *Leptotrichalus* Kleine from Sumatra, Borneo and Continental Asia (Coleoptera: Lycidae) *Ann. Soc. entomol. Fr.* **2000**, 36, 71–184.

Bocak, L. A review of the genus *Microtrichalus* Pic from Sumatra, with notes on Oriental and Australian species (Coleoptera: Lycidae). *Acta Soc. Zool. Bohem.* **2000c**, 64, 3–16.

Bocak, L. **2000d** Revision of the genus *Wakarumbia* (Coleoptera: Lycidae). *Eur. J. Entomol.* 97, 271–278.

Bocak, L. **2001a** A revision of the genus *Diatrichalus* Kleine from New Guinea (Coleoptera: Lycidae). *Stuttg. Beitr. Naturk.*Ser. A. 622, 1–32.

Bocak, L. **2001b** New species of the genus *Wakarumbia* from Sulawesi (Coleoptera: Lycidae). *Raffl. Bull. Zool.* 49, 259–267.

Bocak, L. **2002** Revision and phylogenetic analysis of Metriorrhynchinae. *Eur. J. Entomol.* 99, 315–351.

Bocak, L. **2007** A Revision of *Metriorrhynchus* (Coleoptera: Lycidae) from the Greater Sunda Islands and Continental Asia. *Eur. J. Entomol.* 55, 253–260.

Bocak, L. & Bocakova, M. **1990a** Revision of the supergeneric classification of the family Lycidae (Coleoptera). *Pol. Pismo Entomol.* 59, 623–676.

Bocak, L. & Bocakova, M. **1990b**, Revision of the genus *Hemiconderis* (Coleoptera, Lycidae). *Acta Entomol. Bohemoslov.* 89, 209–220.

Bocak, L. & Bocakova, M. **1991** Revision of the genus *Eniclases* Waterhouse, 1879 (Coleoptera, Lycidae, Metriorrhynchinae) *Mitt. Münch. Entomol. Gess.* **1991**, 81, 203–226.

Bocak, L. & Bocakova, M. **1999** New taxa of Lycidae from Indonesia, China and Nepal. *Acta Univ. Palack. Olom.* 37, 47–56.

Bocak L. & Bocakova M. **2000** Nomenclatural note on Lycidae with a description of a new species (Coleoptera). Acta Univ. Palack. Fac. Rer. Nat.. Biol. (2000) (38, 41–46.

Bocak, L. & Jass, R. **2004** Revision of the genus *Broxylus* Waterhouse (Coleoptera, Lycidae). *D. Entomol. Zeit.* 51, 65–75.

Bocak, L., Matsuda, K., Yagi, T. **2006** A revision of *Metriorrhynchus* from the Philippines with molecular evidence of an Australian origin of the Oriental *Metriorrhynchus* fauna (Coleoptera, Lycidae) *Eur. J. Entomol.* 103, 115–126.

Bocek, M. **2017** New species of *Diatrichalus* (Coleoptera: Lycidae, from New Guinea and the Moluccas. *Zootaxa* 4247, 577–584.

Bocek, M. & Adamkova, K. **2019** New species of Moluccan trichaline net-winged beetles, with remarks on the phylogenetic position and distribution of *Schizotrichalus* (Coleoptera: Lycidae: Metriorrhynchinae). *Zootaxa* 4623, 341–350.

Bocek, M. & Bocak, L. **2016** Where are species limits in polymorphic mimetic beetles from New Guinean mountains: a case of *Eniclases* net-winged beetles (Lycidae: Metriorrhynchini). *ZooKeys* 593, 15–35.

Bocek, M. & Bocak, L. **2017** The molecular phylogeny and classification of trichaline net-winged beetles (Lycidae: Metriorrhynchini) *PeerJ* 5 e3963.

Boisduval, J.B.A.D. **1835** *Voyage de Decouvertes de l'Astrolabe execute par ordre du Roi, pendant les annees 1826-1827-1828-1829, sous le commandement de M.J. Dumont d'Urville. Faune entomologique de*

l'Ocean Pacifique, avec l'illustration des insectes nouveaux recueillis pendant le voyage. 2' Partie. Coléoptères et autres Ordres. J. Tastu: Paris, Vol. 2, vii+716 pp.

Bourgeois, J.**1883** Lycides nouveaux ou peu connus du Musee civique de Genes. *Ann. Mus Civ. Stor. Natur. Gen.* 18, 621–660

Bourgeois, J. **1884** Diagnoses de Lycides nouveaux ou peu connus. 4e partie. *Ann. Soc. entomol. Fr.* (6)4, 61–68.

Bourgeois, J. **1885** Diagnoses de Lycides nouveaux ou peu connus. 5e partie. *Ann. Soc. entomol. Fr.* (6)5, 71–84.

Bourgeois, J. **1886** Lycidae. In: Baer, G.A. Catalogue de Coléoptères des Iles Philippines. *Ann. Soc. entomol. Fr.* (6)6, 98–193.

Bourgeois, J. **1889** Diagnoses de Lycides nouveaux ou peu connus. 6e partie. *Ann. Soc. entomol. Fr.* (6)9, 225–236.

Bourgeois, J. **1892** Lycides nouveaux ou peu connus du Musée civique de Genes. *Ann. Mus Civ. Stor. Natur. Gen.* (2)12, 495–516.

Bourgeois, J. **1897** Description de Malacodermes nouveaux recueillis en Océanie par M. le Dr Ph. François [Col.]. *Bull. Soc. entomol. Fr.* 1897, 72–74.

Bourgeois, J. **1900** Lycides nouveaux ou peu connus de Musee de Genes. Deuxieme memoire. *Ann. Mus. Civ. Stor. Natur. Gen.* 21, 420–432.

Bourgeois, J. **1906** Des Lycides du Sarawak Museum (Bornéo). *Ann. Soc. entomol. Fr.* 75, 184–194.

Bourgeois, J. **1911** Description d'un Lycide nouveaux de Nouvelle-Poméranie (ancienne Nouvelle-Bretagne) [Col.]. *Bull. Soc. entomol. Fr.* 1911, 44–45.

Broun, T. **1893** Manual of the New Zealand Coleoptera. *Parts V—VII.* Wellington: New Zealand Institute. i–xvii+975–1504 pp.

Calder, A.A. **1998.** Coleoptera: Elateroidea. *In:* Wells, A. (Ed.), *Zoological Catalogue of Australia. Vol. 29.6.* CSIRO Publishing: Melbourne. i–xiii + 1–248 pp.

Laporte [de Castelnau], F.L. **1838** Études entomologiques ou descriptiones des insectes nouveaux et observations sur leur synonymie. *Rev. entomol.*, 4[1836], 5–60.

Dalman, J.W. **1818** *In* Schonherr, C.J. *Appendix ad C.J. Schonherr Synonymiam Insectorum sistens descriptiones novarum specierum.* Scarfs: Lewerentz, Vol. 1(3) 266 pp.

Dejean, P.F.M.A. **1836** *Catalogue des coléoptères de la collection de M. le comte Dejean. Troisième édition, revue, corrigée et augmentée.* Méquignon-Marvis Père et Fils: Paris, Livr. 1, xiv + 503 pp.

Dudkova P. & Bocak L. **2010** A review of the Cautires obsoletus species group from Indo–Burma (Coleoptera: Lycidae). *Zootaxa* 2527, 28–48.

Dvorak, M. & Bocak, L. **2007** *Sulabanus* gen. nov., a new genus of Lycidae (Coleoptera) from Sulawesi. *Zootaxa* 1611, 1–24.

Dvorak, M. & Bocak, L. **2009** Ten new species of *Wakarumbia* Bocak, 1999 from Sulawesi (Coleoptera: Lycidae), with a key to males of the genus. *Zootaxa* 2282, 51–61.

Erichson, W.F. **1842** Beitrag zur Fauna von Vandiemensland, mit besonderer Ruecksich auf die geographische Verbreitung der Insecten. *Archiv fuer Naturgeschichte* 8, 83–287.

Fabricius, J.C. **1801** *Systema Eleutheratorum secundum ordines, genera, species: adiectis synonymis, locis, observationibus, descriptionibus*. Bibliopolii Academici: Kiliae, Vol. 2 687 pp.

Fairmaire, L. **1877** Diagnoses de Coléoptères australiens et melanesiens. *Petit. Nouv. Entomol.* 2(180), 166–167.

Fairmaire, L. **1879** Descriptions de Coléoptèresnouveaux ou peu connus du Musée Godeffroy. *Journ. Mus. Godeffroy* 14, 80–114.

Fairmaire, L. **1883** Essai sur les Coléoptères de l'archipel de la Nouvelle-Bretagne. *Ann. Soc. Entomol. Belg.* 27, 1–58.

Gemminger, M. & von Harold, E. **1869** Catalogus coleopterorum hucusque descriptorum synonymicus et systematicus. Band 6. Rhipiceridae–Cionidae. Pp. 1609–1800.

Guérin-Méneville, F.E. **1830** Crustacés, arachnides et insectes in Duperrey, L. I. (Ed.), *Voyage autour du monde ... sur la Corvette de Sa Majesté, La Coquille, pendant les annés 1822, 1823, 1824 et 1825*. Zoologie. Bertrand: Paris. 22 color plates.

Guérin-Méneville, F.E. **1838** Crustacés, arachnides et insectes in Duperrey, L. I. (Ed.), *Voyage autour du monde sur la Corvette de Sa Majesté, La Coquille, pendant les annés 1822, 1823, 1824 et 1825*. Zoologie, vol. 2, part 2.

Kalousova, R. & Bocak, L. **2017** Species delimitation of colour polymorphic *Cladophorus* (Coleoptera: Lycidae) from New Guinea. *Zootaxa* 4320, 505–522.

Kazantsev, S.V. **2006** New taxa of net-winged beetles (Coleoptera, Lycidae) from Sri Lanka. *Eurasian Entomol. J.* 5, 127–131.

Kazantsev, S.V. **2007** Marena gen.n., new Metriorrhynchini genus from New Guinea (Coleoptera: Lycidae). Russ. Entomol. J. 16, 297–300.

Kazantsev, S.V. **2009** On the status and systematic position of the genera *Falsolucidota* Pic, 1921 and *Macrolycinella* Pic, 1922, with notes on *Lopheros* LeConte, 1881 (Coleoptera: Lycidae). *Russ. Entomol. J.* 18, 277–283

Kazantsev, S.V. **2010**. New Taxa of Papuan net-winged beetles (Lycidae, Coleoptera). *Latv. Entomol.* 48, 92–100.

Kazantsev, S.V. **2015a** New *Xylobanus* and related taxa of net-winged beetles from New Guinea (Coleoptera: Lycidae). *Russ. Entomol. J.* **2015**, 24, 107–118.

Kazantsev, S.V. **2015b** New species of *Metriorrhynchus* Gemminger et Harold, 1869 and Porrostoma Laporte, 1838 from New Guinea (Coleoptera: Lycidae). *Russ. Entomol. J.* **2015**, 24, 211–233.

Kazantsev, S.V. **2016a** A review of *Ditua* Waterhouse, 1879 with description of seven new species from New Guinea (Coleoptera: Lycidae). *Russ. Entomol. J.* 25, 239–248.

Kazantsev, S.V. **2016b** New species of *Procautires* Kleine, 1925 from New Guinea (Coleoptera: Lycidae). *Russ. Entomol. J.* **2016**, 25, 35–47.

Kazantsev, S.V. **2016c** New and little-known species of *Falsolucidota* Pic, 1921from New Guinea (Coleoptera: Lycidae). *Russ. Entomol. J.* **2016**, 25, 355–360.

Kirsch T. **1875** Neue Käfer aus Malacca". *Mitt. Koenigl. Zool. Mus. Dresden* 1, 3–34.

Kirsch T. **1877** Beithrag zur Kenntniss der Coleopteren-Fauna von Neu Guinea. *Mitt. Koenigl. Zool. Mus. Dresden* 3, 135–161.

Kleine, R. **1925a**, Dritter Beitrag zur kenntnis der Lycidae. Die neue Gattung *Leptotrichalus*. *Philipp. J. Sci.* 28, 295–311.

Kleine, R. **1925b** Fauna Buruana. Coleoptera, Fam. Lycidae. (2. Beitrag zur Kenntnis der Lycidae.). *Treubia*, 7, 31–37.

Kleine, R. **1926a** Coleoptera Lycidae. *Nova Guinea* 13, 91–195.

Kleine, R. **1926b** Die Lyciden der Philippinen-Inseln. *Philipp. J. Sci.* 31, 33–114.

Kleine, R. **1927a** Neue Lycidae von den Sunda-Inseln. *Treubia* 9, 293–316.

Kleine, R. **1927b** *Libnetomimus* eine neue Lycidengattung von den Philippinen-Inseln. *Soc. Ent*. **1**, 42, 1–2, 4 ff.

Kleine, R. **1927c**, Bestimmungstabelle der Gattung *Xylobanus* C. O. Waterhouse. *Tijdschr. Ent*. 70, 43–72.

Kleine, R. **1928a** Dr. Mjoebergs Lycidenausbeute aus dem ostliehen Borneo. *Stett. Ent. Zeit.* 89, 313–331.

Kleine, R. **1928b** Neue *Metriorrhynchus* von Australien and Batjan (Col. Lycid.). *Ent. Mitt*. 17, 120–128.

Kleine, R. **1928c** Neue Indische Lycidae nebst faunistischen Bermerkungen. *Ind. Forest Rec. Ent.* 13, 221–268.

Kleine, R. **1929** Bestimmungstabelle der Gattung *Trichalus*. *Treubia* 10, 471–493.

Kleine, R. **1930a** Zur Lycidenfauna der kleinen Sunda-Inseln. *Treubia* 11, 341–347.

Kleine, R. **1930b** Bestimmungstabelle der Trichalusverwandtschaft. *Treubia* 9, 325–340.

Kleine, R. **1930c** Neue Lyciden der Malaiischen subregion. *J. Fed. Malay Mus.* 16, 169–174.

Kleine, R. **1930d** Nova Guinea

Kleine, R. **1931** Zur kenntnis der Lycidenfauna Javas. *Treubia* 13, 245–290.

Kleine, R. **1932** Neue Brenthidae und Lycidae von den Philippinen. *Philipp. J. Sci*. 49, 571–579.

Kleine, R. **1933a** Lycidae. Part 128. In Schenkling S. (ed.) *Coleopterorum Catalogue auspiciis et auxilio*. W. Junk: Berlin, 145 pp.

Kleine, R. **1933b** Neue Lyciden und Bemerkungen zum Cat. Col. Junk-Schenkling Lycidae. *Stett. Ent. Zeit*. 94, 1–20.

Kleine, R. **1934** Prof. Dr E. Handschin, Studienreise auf den Sundainseln und in Nordaustralien, 1930–32. 5. Brenthidae und Lycidae. *Mitt. Schweiz. Entomol. Ges.* 16, 115–117.

Kleine, R. **1935a** Bericht über die von Miss Cheesman in Britisch Neu-Guinea gesammelten Brenthiden und Lyciden. *Nova Guinea* 17, 303–322.

Kleine, R. **1935b** Neue Brenthiden und Lyciden (Col.). von den Salomonen. *Arb. Morph.- Tax. Ent.* 2, 174–182.

Kleine, R, **1935c** Fünf neue Lycidae vom Edie Creek, Britisch Neu Guinea. Nova Guinea 17, 151–154.

Kleine, R. **1936** Neue Lycidae aus Java (Coleoptera). *Arb. Morph. Taxon. Ent*. 3, 132–140.

Kleine, R. **1939a** Neue Brenthiden und Lyciden die Miss Cheesman 1936 in Hollandisch Neu-Guinea gesammelt hat. *Nova Guinea* (n. s.) 3, 106–112.

Kleine, R. **1939b** Neue Brenthiden und Lyciden von den Salomon-Inseln. *Ann. Mag. Nat. Hist*. (11)4, 587–591.

Kleine, R. **1939c** Neue Lyciden von den Orientalischen Inseln und Molukken. *Treubia* 17, 5 –19.

Kleine, R. **1939d** Neue Brenthiden und Lyciden aus den Hollandischen Kolonien. *Treubia* 17, 131–135.

Kleine, R. **1943** Neue Lyciden aus dem Ungarischen National-Museum (Coleopt.). *Ann. Hist.-Nat. Mus. Nation. Hung*. 36, 145–156.

Kubecek, V., Bray, T. C. & Bocak, L. **2015** Molecular phylogeny of Metanoeina net-winged beetles identifies *Ochinoeus*, a new genus from China and Laos (Coleoptera: Lycidae). *Zootaxa*, 3955, 113–122.

Kubecek, V., Dvorak, M. & Bocak, L. **2011** The phylogenetic structure of Metriorrhynchini fauna of Sulawesi, (Coleoptera: Lycidae) with description of a new genus. *Zool. Stud.* 50, 645–656.

Kusy, D. **2017** A new species of *Synchonnus* (Coleoptera: Lycidae) from New Guinea, with an identification key to the Papuan species. *Acta entomol. mus. nat. Pragae* 57, 153–160

Kusy, D., Sklenarova, K. & Bocak L. **2018** The effectiveness of DNA-based delimitation in *Synchonnus* net-winged beetles (Coleoptera: Lycidae) assessed, and description of 11 new species. *Austral Entomol.* 57, 25–39.

Lea, A.M. **1895** Descriptions of new species of Australian Coleoptera. *Proc. Linn. Soc. N. S. W.* (2)9, 589–634.

Lea, A.M. **1899** Descriptions of new species of Australian Coleoptera. Part V. *Proc. Linn. Soc. N. S. W.* 23, 521–645.

Lea, A.M. **1908** The Coleoptera of King Island, Bass Strait. *Proc. R. Soc. Vic.* (n.s.) 20, 143–207.

Lea, A.M. **1909** Revision of the Australian and Tasmanian Malacodermidae. *Trans. Entomol. Soc. Lond*. 1909, 45–251.

Lea, A.M. **1921a** On Coleoptera, mostly from Queensland. *Mem. Qld Mus.* 7, 182–240.

Lea, A.M. **1921b** On Australian Coleoptera of the family Malacodermidae. *Trans. R. Soc. S. Aust.* 45, 50–135.

Lea, A.M. **1921c** Descriptions of new species of Australian Coleoptera. Part XVII. *Proc. Linn. Soc. N. S. W.* 46, 351–369.

Lea, A.M. **1922** Results of Dr E. Mjoberg's Swedish Scientific Expeditions to Australia 1910–1913. 26. Cryptophagidae, Cucujidae, Malacodermidae, Melandryidae, Mordellidae, Rhipidophoridae and Oedemeridae. *Ark. Zool*. 14(11), 1–21.

Lea, A.M. **1929** On Coleoptera, mostly from Queensland. *Mem. Qld Mus.* 9, 335–363.

Le Guillou, M. Description de vingt insectes Coléoptères recueillis pendant le voyage autour du monde de *l'Astrolabe* et *la Zelee. Rev. Zool.* **1844**, 7, 220–225

Macleay, W.J. **1872** Notes on a collection of insects from Gayndah. Trans. *Entomol, Soc N. S. W.* 2(4), 239–318.

Macleay, W.J. **1886** The insects of the Fly River, New Guinea, "Coleoptera". *Proc. Linn. Soc. N. S. W.* (2)1, 136–157.

Macleay, W.J. **1887** The Insects of the Cairns District, Northern Queensland. *Proc. Linn. Soc. N. S. W.* (2)2, 211–238.

Montrouzier, P. **1857** Essai sur la faune de l'île de Woodlark ou Mouiou. *Ann. Soc. Agr. Lyon* 7, 1–114.

Pic, M. **1921** Contribution a l'étude des Lycides. *L'Echange, Rev. Linn.* 404–406, 1–12, hors texte.

Pic, M. **1922** Contribution a l'étude des Lycides. *L'Echange, Rev. Linn.* 407–410, 13–28, hors texte.

Pic, M. **1923a** Contribution a l'étude des Lycides. *L'Echange, Rev. Linn.* 411–413, 29–40, hors texte.

Pic, M. **1923b** Nouveautés diverses. *Mél. exot.-entomol.* 38, 1–32.

Pic, M. **1923c** Malacodermes exotiques. *L'Echange, Rev. Linn.* 418–419, 1–4, hors texte.

Pic, M. **1923d** Faune Entomologique de L'Indochine Francaise. Lycidae. Opuscules del L'Institut Scientifique De L'Indochine, Saigon 1 60 pp.

Pic, M. 1**925b** Noms nouveaux et correction concernant divers Lycides [COL.]. *Bull. Soc. Entomol. Fr.* 1925(5), 96.

Pic, M. **1926a** Remarques critiques, synonymies et diagnoses [Col.]. *Bull. Soc. Zool. Fr.* 51, 451–455.

Pic, M. **1926b** Malacodermes exotiques. *L'Echange, Rev. Linn.* 423–426, 21–36, hors texte.

Pic, M. **1927** Malacodermes exotiques. *L'Echange, Rev. Linn.* 427–430, 37–52, hors texte.

Pic, M. **1928** Nouveautés diverses. *Mél. exot.-entomol.* 52, 1–32.

Pic, M. **1929** Nouveautés diverses. *Mél. exot.-entomol.* 53, 1–32.

Pic, M. **1930** Malacodermes exotiques. *L'Echange, Rev. Linn.* 439–442, 77–92, hors texte.

Pic, M. **1931** Nouveautes diverses. *Bull. Soc. Agr. Lyon* 10, 25–26.

Pic, M. **1932** Lycidae. In: Résultats Scientifiques du Voyage aux Indes Orientales Neérlandaises de LL. AA. RR. le Prince et la Princesse Léopold de Belgique publiés par V. van Straelen. *Mém. Mus. Roy. Hist. Nat. Belgique, hors séries* Vol. 4(4), 100 pp.

Redtenbacher, L. **1867** *Reise der österreichischen Fregatte Novara um die Erde in den Jahren 1857, 1858, 1859 unter den Befehlen des Commodore B. von Wüllerstorf-Urbair.* Zoologischer Theil. Zweiter Band. Coleopteren. Kaiserlich-Königlichen Hof- und Staatsdruckerei: Wien. IV, 249 pp., 5 Tables.

Schaufuss, L.W. **1887** Beitrag zur Fauna der Niederländischen Besitzungen auf den Sunda-Inseln. *Hor. Soc. Entomol. Ross*. 21, 102–147.

Sklenarova, K. & Kubecek, V. Bocak L. **2014** Subtribal classification of Metriorrhynchini (Insecta: Coleoptera: Lycidae): an integrative approach using molecular phylogeny and morphology of adults and larvae. *Arthr. Syst. & Phyl.* 72, 37–54.

Strand, E. **1936** Miscellanea nomenclatorica zoologica et paleontological. *Fol. Zool. Hydrobiol*. 9, 167–170.

Waterhouse, C.O. **1877** A Monograph of the Australian species of the Coleopterous family Lycidae. *Trans. Entomol. Soc. Lond.* 1877, 73–86.

Waterhouse, C.O. **1878** On the different forms occurring in the Coleopterous family Lycidae, with descriptions of new genera and species. *Transactions of the Entomological Society of London*, 1878, 95–118.

Waterhouse, C.O. **1879** Illustration of the typical specimens of Coleoptera in the collection of the British Museum. Part I. – Lycidae. London: British Museum, 93 pp.

Waterhouse, C.O. **1884** Coleoptera collected during the expedition of H.M.S. 'Challenger'. *Ann. mag. nat. hist* (5)13, 73–78.
